# Supplementary material for: Impact of color-coded and warning nutrition labelling schemes: A systematic review and network meta-analysis
Source: PLoS Med. 2021 Oct 5;18(10):e1003765. doi: 10.1371/journal.pmed.1003765 (PMC8491916; doi:10.1371/journal.pmed.1003765)
Supplement: S1 Text — (DOCX) [file pmed.1003765.s001.docx]

**S1 Text. Supplementary Methods**

**Network meta-analysis**

In this systematic review, a range of front-of-package labelling (FOPL) systems were included as the competing intervention groups and we aimed to find out whether one FOPL system is more or less effective than other FOPL systems in modifying consumers’ behaviours. Additionally, very few intervention studies compared the effects of two FOPLs directly, which made it difficult to make multiple comparisons using the conventional meta-analysis method. For these reasons, we applied the frequentist network meta-analysis (NMA) method to synthesize studies, which allowed for the simultaneous analysis of more than two interventions.

Technically, NMA generates network estimates by pooling indirect and direct comparisons between interventions. Direct comparisons refer to the comparisons between two competing interventions, which often didn’t exist or were quite limited. Indirect comparisons evaluate the effect of interventions against the same control group which usually resulted from randomised controlled trials or quasi-experiment trials. With sufficient indirect comparisons, it is possible for NMA to estimate the direct comparison based on the network graph. A simple example is given below:


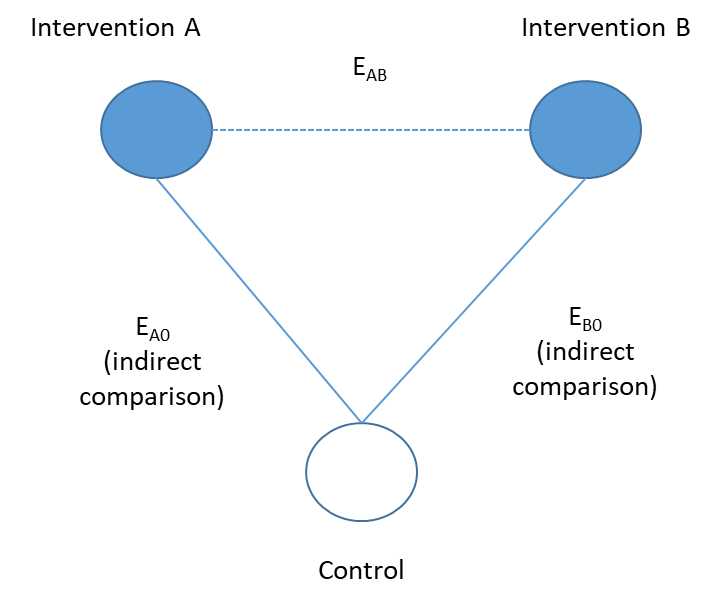


The direct effect estimated by NMA (EAB) can be calculated using the effects of indirect comparisons (EA0, EB0): EAB = EA0 - EB0. The estimated direct effect was then combined with the observed direct effect provided by previous studies (if available), which makes full use of all information available and draws a robust conclusion of what is the most (or least) beneficial intervention given all assumptions are met.

The key assumptions of NMA are “transitivity” and “consistency”. The “transitivity”, in principle, ensures that we can calculate the direct effect of intervention A versus B (A vs B) via A vs C and B vs C using the above-mentioned equation. In other words, the Group C is transitive. However, the “transitivity” assumption is more of a conceptual assumption, and a statistical assessment of the “consistency” assumption is needed to provide evidence on whether direct and indirect comparisons are consistent across interventions. The inconsistency is often evaluated in two levels: global and local. The global test provides an overall inconsistency based on all comparisons across different interventions, while the local test examines the inconsistency for each comparison individually. In our study, a node-splitting method is used to evaluate the consistency in NMA models, which splits our network estimates into direct and indirect evidence using a back-calculation method. The violation of “consistency” assumption may indicate that the distribution of covariates or effect modifiers is not balanced across original studies. Therefore, in our study we only used the direct evidence for interpretation when there was a significant inconsistency between direct and indirect estimates (p < 0.05). In addition, sensitivity analysis or subgroup analysis is usually conducted to explore the effect modifiers, and meta-regression is usually recommended to adjust for the inconsistency. In our study, we performed a sensitivity analysis including only randomised controlled trials to examine the possible effect modification led by study design, as well as a series of subgroup analysis to explore the influence of a range of covariates, such as age group, sex, study settings, etc. Due to the limited number of studies for most of the intervention groups and outcome measures, we did not perform the NMA meta-regression which would require a large number of studies to ensure a sufficient statistical power.

**Risk of bias assessment**

The ROB 2 tool is made up of 5 domains of bias: randomisation process, deviation from intended interventions, missing outcome data, measurement of outcome, and selection of reported results. For each domain, several questions were included to assess different aspects of each domain of bias, and for each question a response was provided by choosing an answer from "Yes", "Probably Yes", "No information", "Probably no" or "No". Then a domain-specific overall assessment of bias was classified as "Low risk", "High risk" or "Some concerns" using recommended algorithms. Finally, a summary of biases across domains was grouped into (1) "Low risk" if the study is judged to be at low risk of bias for all domains for this result; (2) "Some concerns" if study is judged to raise some concerns in at least one domain, but not to be at high risk of bias for any domain; or (3) "High risk" if the study is judged to be at high risk of bias in at least one domain. The full questionnaires and algorithms summarising the domain-specific risk of bias are available at https://www.riskofbias.info/welcome/rob-2-0-tool.

The ROBINS-I tool is made up of 7 domains of bias: bias due to confounding, bias in selection of participants into the study, bias in classification of interventions, bias due to deviations from intended interventions, bias due to missing data, bias in measurement of outcomes, and bias in selection of the reported results. For each domain, several questions were included to assess different aspects of each domain of bias, and for each question a response was provided by choosing an answer from "Yes", "Probably Yes", "No information", "Probably no" or "No". Then a domain-specific overall assessment of bias was classified as "Low risk", "Moderate risk", "Serious risk of bias" or "No information". Finally a summary of biases across domains was grouped into (1) "Low risk" if the study is judged to be at low risk of bias for all domains for this result; (2) "Moderate" if study is judged to be at low or moderate risk for all domains; (3) "Serious risk" if the study is judged to be at serious risk of bias in at least one domain, but not at critical risk of bias in any domain; (4) "Serious" if study is judged to be at critical risk of bias in at least one domain; or (5) "No information" if there is a lack of information in one or more key domains, and there is no clear indication that the study is at serious or critical risk of bias. To integrated with the risk of bias assessment results of randomised controlled studies using ROB 2, we categorised "Serious risk" and "Critical risk" into "High risk", as well as "No information" and "Moderate risk" into "Some concerns", when summarising risk across domains as "High risk". The full questionnaires and criteria used for summarising the domain-specific risk and overall risk are available at https://www.riskofbias.info/welcome/home/current-version-of-robins-i/robins-i-tool-2016.

The NHLBI study quality assessment tools is made up of 13 different aspects of bias in observational cohort and cross-sectional studies, including: research question, study population, groups recruited from the same population and uniform eligibility criteria, sample size justification, exposure assessed prior to outcome measurement, sufficient timeframe to see an effect, different levels of the exposure of interest, exposure measures and assessment, repeated exposure assessment, outcome measures, blinding of outcome assessors, follow-up rate and statistical analyses. For each question, a response was provided by choosing an answer from "Yes", "No information", or "Others (cannot determine, not applicable or not reported)". Then a domain-specific overall assessment of bias was classified as "Low risk", "Moderate risk", "Serious risk of bias" or "No information". The full questionnaires and criteria for each response are available at https://www.nhlbi.nih.gov/health-topics/study-quality-assessment-tools.

**Outcome measurements**

For categorical outcomes, odds ratios (ORs) and 95% confidence intervals (95%CIs) were collected from original studies as the summary estimates for categorical outcomes. When ORs and 95%CIs (or coefficient of label type in logistic regression model [β] and its standard error [SE]) were not directly provided for FOPL of interest in our study, two researchers (J.S and M.B) calculated the unadjusted ORs and 95%CIs independently, and double-checked by a third researcher (M.T) if there was any inconsistency. The formula is set up based on a 2 x 2 table:

$Odds ratio=ad/bc$ (1)

$SE\left( odds ratio \right)=\sqrt{\frac{1}{a}+\frac{1}{b}+\frac{1}{c}+\frac{1}{d}}$ (2)

In formula (1) and (2), a and b represent frequencies in the intervention group (or follow-up) with and without an outcome observed (e.g., selection of a healthier options in a food category), while c and d stand for frequencies in the control group (or baseline) with and without an outcome observed.

For continuous outcomes, relative mean difference and SE were estimated using the following formula:

$Relative mean difference=(X2-X1)/X1$ (3)

$SE\left( relative mean difference \right)=\frac{X2}{X1}\sqrt{\left[ \frac{{SE(X1)}^{2}}{{SE(X1)}^{2}}+\frac{{SE(X2)}^{2}}{{SE(X2)}^{2}}-2\rho\frac{SE(X1)\times SE(X2)}{X1\times X2} \right]}$ (4)

In formula (3) and (4), X1 and X2 represent estimate of continuous outcome in control (or baseline) and intervention (or follow-up) condition respectively; ρ is the correlation coefficient between sample in intervention condition and control condition, where ρ= 0 when participants in both intervention and control conditions were separately collected from different population, ρ= 0.9 for studies with participants remained the same in both intervention and control conditions. For intervention trials with pre-post design in both intervention and control group, relative mean difference and SE were calculated with alternative formula as follow:

$Relative mean difference=\frac{\left( X2-X20 \right)-\left( X1-X10 \right)}{X1-X10}$ (5)

$SE\left( relative mean difference \right)=\sqrt{\frac{{X2}^{2}}{{X20}^{2}}\left[ \frac{{SE\left( X20 \right)}^{2}}{{SE\left( X20 \right)}^{2}}+\frac{{SE\left( X2 \right)}^{2}}{{SE\left( X2 \right)}^{2}}-2\rho\frac{SE\left( X20 \right)\times SE\left( X2 \right)}{X20\times X2} \right]+\frac{{X1}^{2}}{{X10}^{2}}\left[ \frac{{SE\left( X10 \right)}^{2}}{{SE\left( X10 \right)}^{2}}+\frac{{SE\left( X1 \right)}^{2}}{{SE\left( X1 \right)}^{2}}-2\rho\frac{SE\left( X10 \right)\times SE\left( X1 \right)}{X10\times X1} \right]}$ (6)

where X1 and X10 represent estimate of continuous outcome of baseline and follow-up condition in control group, while X2 and X20 represent intervention group. ρ= 0.9 in (6).
