# Supplementary figures and images for: Impact of color-coded and warning nutrition labelling schemes: A systematic review and network meta-analysis
Source: PLoS Med. 2021 Oct 5;18(10):e1003765. doi: 10.1371/journal.pmed.1003765 (PMC8491916; doi:10.1371/journal.pmed.1003765)

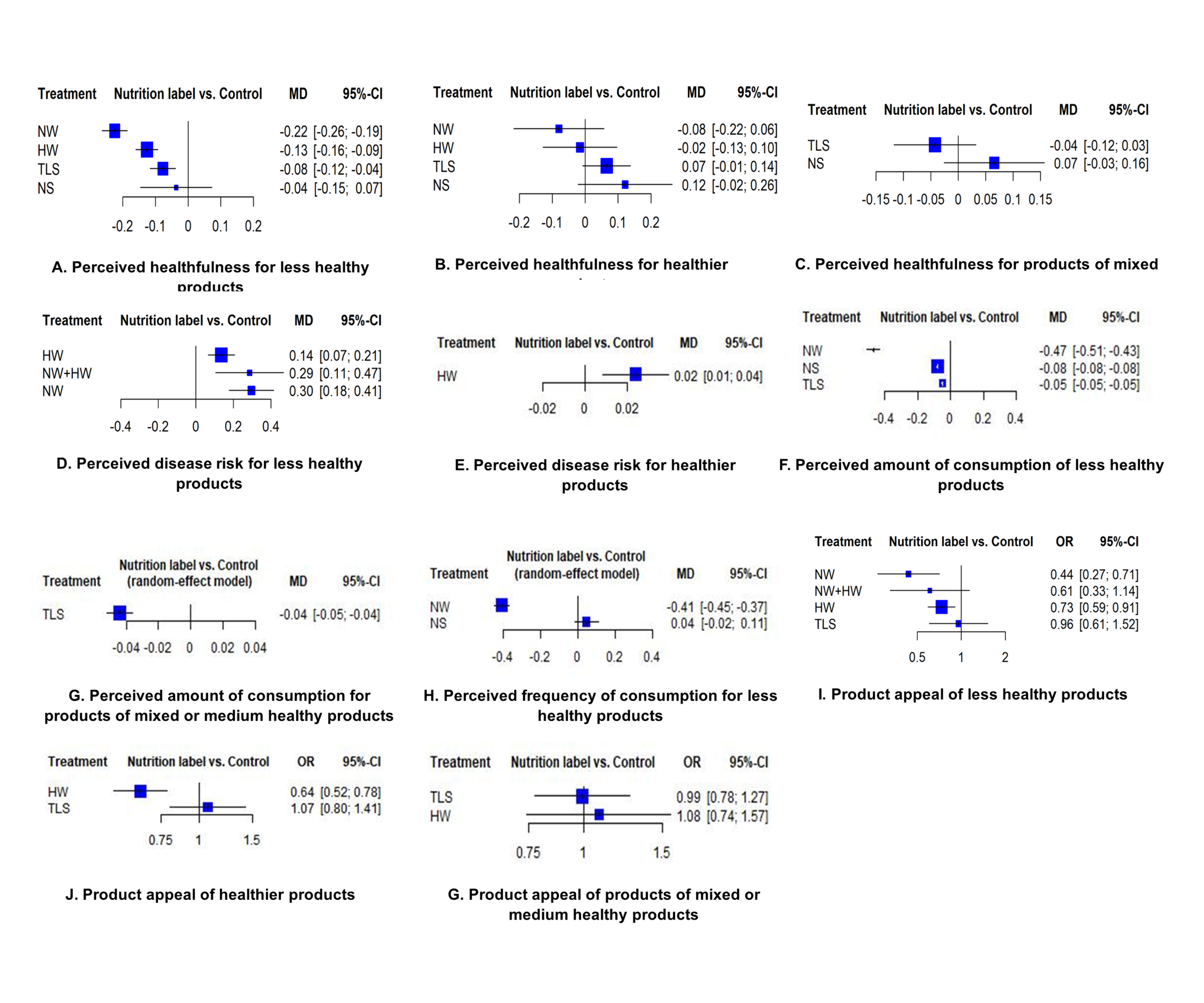

Supplement: S1 Fig — CI, confidence interval; HW, health warning; MD, mean difference; NS, Nutri-Score; NW, nutrient warning; OR, odds ratio; TLS, traffic light labelling system. (TIFF) [file pmed.1003765.s003.tiff]

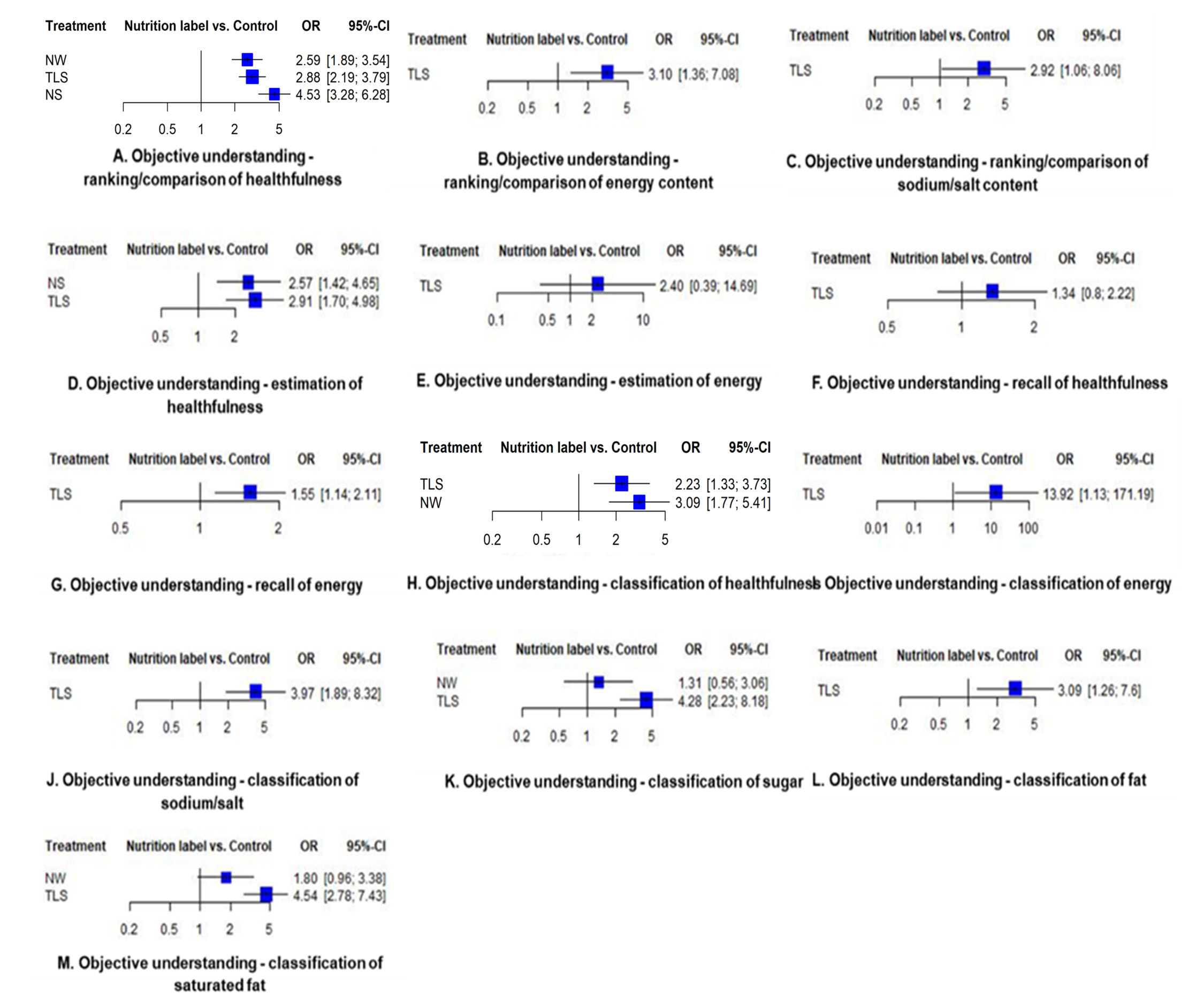

Supplement: S2 Fig — CI, confidence interval; NS, Nutri-Score; NW, nutrient warning; OR, odds ratio; TLS, traffic light labelling system. (TIFF) [file pmed.1003765.s004.tiff]

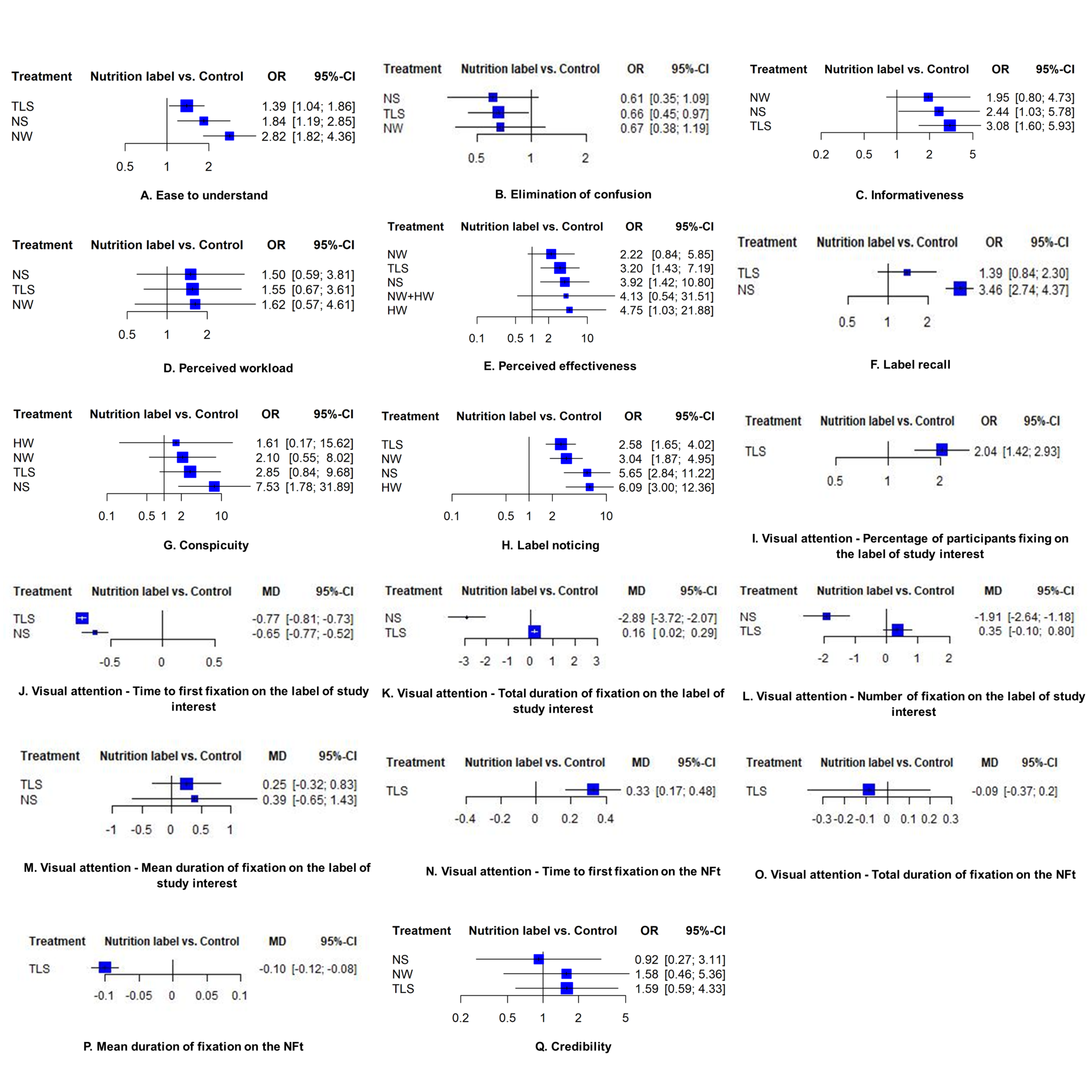

Supplement: S3 Fig — CI, confidence interval; HW, health warning; MD, mean difference; NFt, Nutrition Facts table; NS, Nutri-Score; NW, nutrient warning; OR, odds ratio; TLS, traffic light labelling system. (TIFF) [file pmed.1003765.s005.tiff]

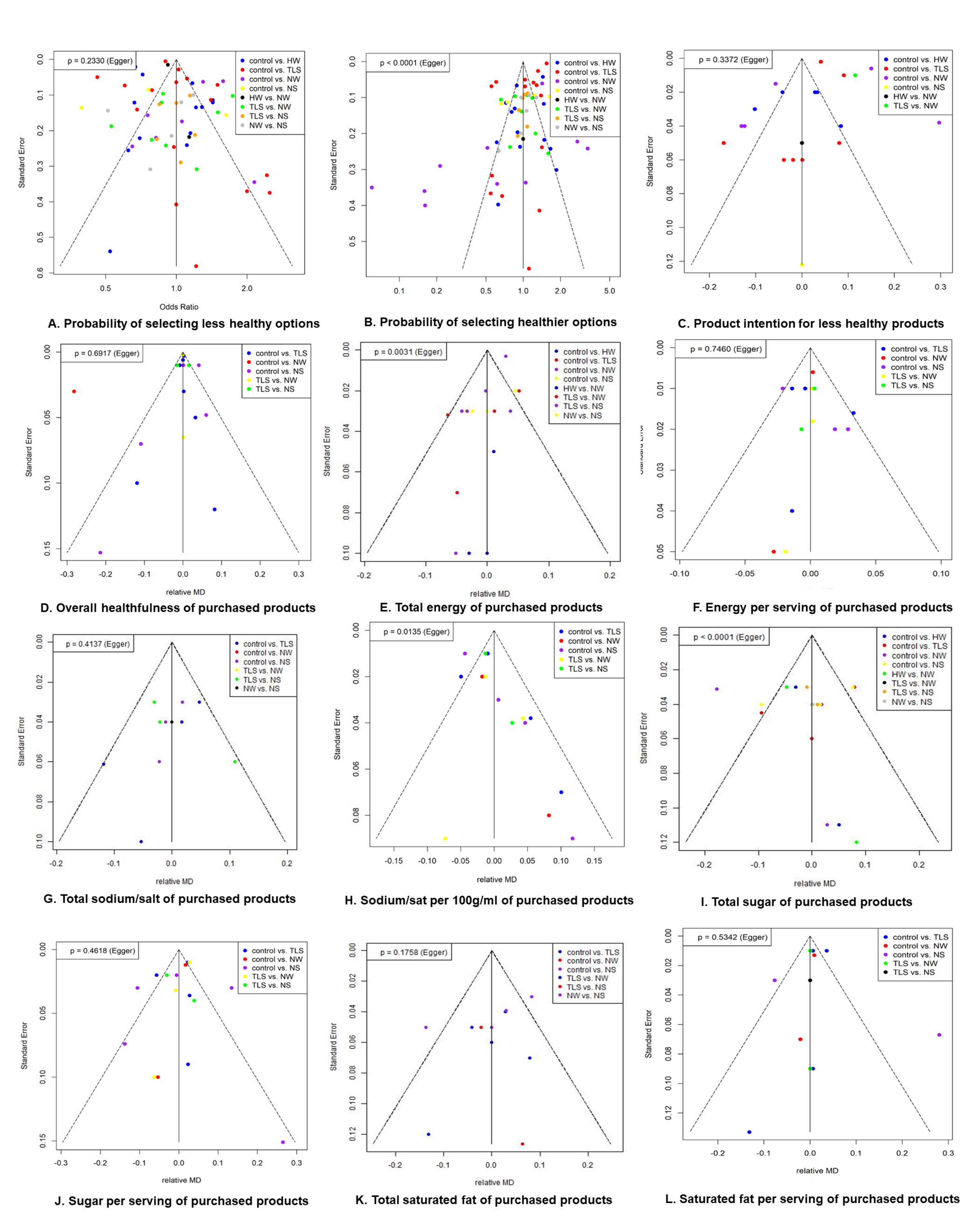

Supplement: S4 Fig — HW, health warning; MD, mean difference; NS, Nutri-Score; NW, nutrient warning; TLS, traffic light labelling system. (TIFF) [file pmed.1003765.s006.tiff]

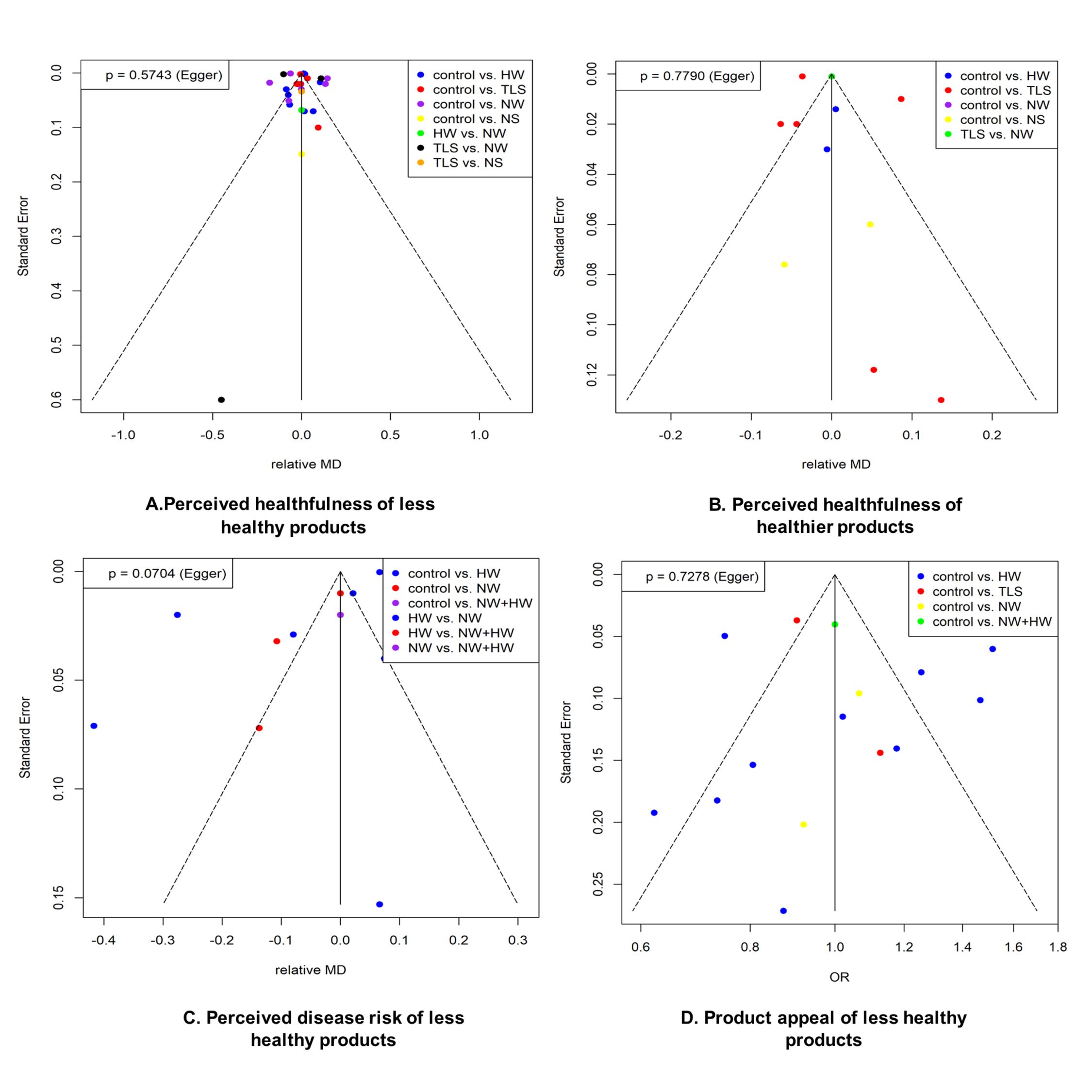

Supplement: S5 Fig — HW, health warning; MD, mean difference; NS, Nutri-Score; NW, nutrient warning; OR, odds ratio; TLS, traffic light labelling system. (TIFF) [file pmed.1003765.s007.tiff]

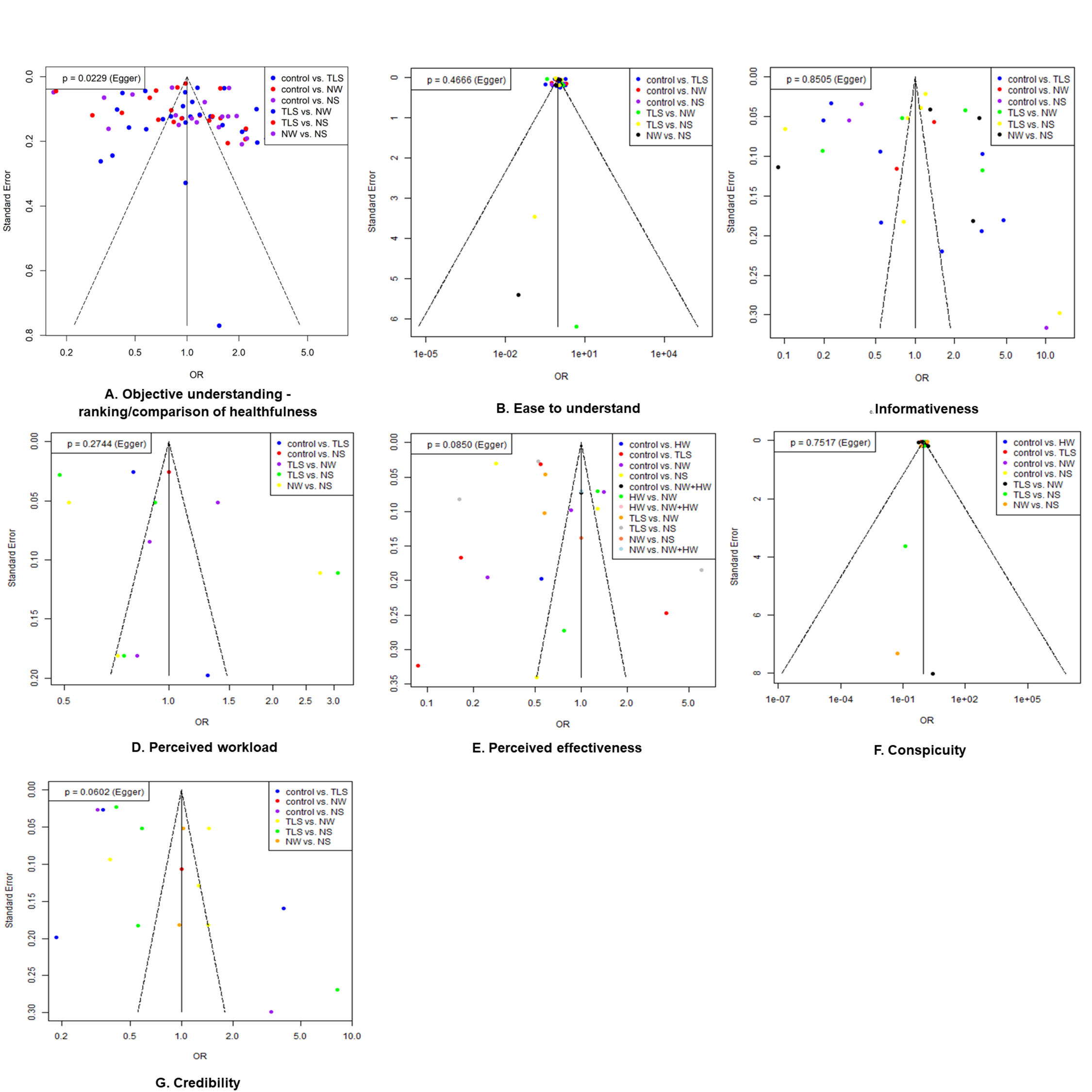

Supplement: S6 Fig — HW, health warning; NS, Nutri-Score; NW, nutrient warning; OR, odds ratio; TLS, traffic light labelling system. (TIFF) [file pmed.1003765.s008.tiff]
