# Supplementary material for: Impact of color-coded and warning nutrition labelling schemes: A systematic review and network meta-analysis
Source: PLoS Med. 2021 Oct 5;18(10):e1003765. doi: 10.1371/journal.pmed.1003765 (PMC8491916; doi:10.1371/journal.pmed.1003765)
Supplement: S2 Table — (PDF) [file pmed.1003765.s010.pdf]

S2 Table. Characteristics of studies included in the systematic review

article ID	Reference No.	study	country	language	total sample size	NFt present along with treatme	time pressure	comparator	study design category	country income	age (mean or range)	age group	proportion of female	sex	race	education (bachelor degree)	income_individiual/household	occupation (%major)	product	product type	real-world setting	field of real-world setting	label location	warning label	Chilean Warning Label system	other warning label design	nutrition disclosure	health effect	textual WL	textual+non-textual WL	positively framing	negatively framing	color-coded	TLS	summary TLS	detailed TLS	Nutri-score/5-CNL
1	1	Burton 1993	US	English	343	N	N	no label	nonequivalent control group design: posttest design	high	ns	ns	ns	mostly female	ns	ns	ns	ns	Cereal and cereal products, Dairy	multiple	N		package	Y	N	Y	N	Y	Y	N	N	Y	N	N	N	N	N
2	2	Bushman 1998	US	English	240	N	N	no label	randomized controlled trial	high	ns	ns	0.5	mixed	ns	ns	ns	ns	Dairy	single	N		package	Y	N	Y	N	Y	Y	N	N	Y	N	N	N	N	N
7	3	Jones 2007	UK	English	92	Y	N	no label	Interrupted Time-Series Design	high	31.5	adults or most adults	0.728	mostly female	ns	ns	ns	ns	NS	NS	N		package	N	N	N	N	N	N	N	N	N	Y	Y	N	Y (simple MTL)	N
10	4	Borgmeier 2009	Germany	English	252	N	N	no label	randomized controlled trial	high	36	adults or most adults	0.536	mixed	ns	high	ns	ns	general food and beverage products	multiple	N		package	N	N	N	N	N	N	N	N	N	Y	Y	N	Y (detailed MTL + interpretative texts, detailed	N
12	5	Drichoutis 2009	Greece	English	29	N	N	no label	choice conjoint task	high	ns	ns	ns	mostly female	ns	ns	ns	ns	Convenient foods	single	N		package	N	N	N	N	N	N	N	N	N	Y	Y	N	Y (detailed MTL)	N
13	6	Gorton 2009	New Zealand	English	1525	N	N	NFt	cross-sectional	high	41	adults or most adults	0.7	mostly female	ns	low	mix	ns	snack foods	single	N		package	N	N	N	N	N	N	N	N	N	Y	Y	Y	Y (simple MTL, detailed MTL)	N
15	7	Sacks 2009	UK	English	sales data	Y	N	no label	one-group design: pretest-posttest design	high									Convenient foods	single	Y	supermarkets and gocery stores	package	N	N	N	N	N	N	N	N	N	Y	Y	N	Y (detailed MTL)	N
17	8	Fatimah 2010	Malaysia	English	232	Y	N	no label	nonequivalent control group design: pretest-posttest design	upper-middle	20-59	adults or most adults	1	mostly female	mix	low	ns	ns	NS	NS	N		package	N	N	N	N	N	N	N	N	N	Y	Y	N	Y (detailed MTL+interpretive text, detailed MTL)	N
23	9	Sacks 2011	Australia	English	sales data	Y	N	no label	nonequivalent control group design: pretest-posttest design	high									Bread & dairy	multiple	Y	supermarkets and gocery stores (online)	package	N	N	N	N	N	N	N	N	N	Y	Y	N	Y (simple MTL + interpretative texts: fat, saturated fat, sugar	N
24	10	van Herpen 2011a	US	English	51	N	N	no label	Interrupted Time-Series Design	high	25.8	adults or most adults	0.55	mixed	mix	low	low	ns	restaurant meals	multiple	N		package	N	N	N	N	N	N	N	N	N	Y	Y	Y (base on calories, added sugar and fat)	N	N
24	10	van Herpen 2011b	US	English	41	N	N	no label	randomized controlled trial	high	27.8	adults or most adults	ns	mixed	mix	low	low	ns	restaurant meals	multiple	N		package	N	N	N	N	N	N	N	N	N	Y	Y	Y (base on calories, added sugar and fat)	N	N
25	11	van Herpen 2011c	Netherland	English	156	N	N	no label	randomized controlled trial	upper-middle	20.8	adults or most adults	0.65	mostly female	ns	ns	ns	ns	Cereal and cereal products	single	N		package	N	N	N	N	N	N	N	N	N	Y	Y	N	Y (detailed MTL)	N
25	11	van Herpen 2011d	Turky	English	131	N	Y	no label	randomized controlled trial	high	>19	adults or most adults	0.533	mixed	ns	ns	ns	ns	Cereal and cereal products	multiple	N		package	N	N	N	N	N	N	N	N	N	Y	Y	N	Y (detailed MTL)	N
27	12	Ares 2012	Uruguay	English	178	N	N	no label	choice conjoint task	high	>18	adults or most adults	0.58	mixed	ns	ns	ns	ns	Bread & bakery products, Dairy	multiple	N		package	N	N	N	N	N	N	N	N	N	Y	Y	N	Y (detailed MTL)	N
32	13	Koenigstorfer 2012	Germany	English	184	N	N	no label	nonequivalent control group design: posttest design	high	ns	ns	ns	ns	ns	ns	ns	ns	Cereal and cereal products	single	Y	supermarkets and gocery stores	package	N	N	N	N	N	N	N	N	N	Y	Y	Y	N	N
33	14	McLean 2012	New Zealand	English	691	Y	N	no label	choice conjoint task	high	50	adults or most adults	0.57	mixed	white	low	ns	ns	Fruit and vegetables	single	N		package	N	N	N	N	N	N	N	N	N	Y	Y	N	Y (simple MTL + interpretative text: fat, saturated fat, sugar	N
36	15	Roberto 2012	US	English	409	N	N	no label	randomized controlled trial	high	46.01	adults or most adults	0.526	mixed	white	low	mix	ns	Non-alcoholic beverages, Bread & bakery products, Cereal and cereal products, Sauces and spreads, snack foods	multiple	N		package	N	N	N	N	N	N	N	N	N	Y	Y	N	Y (simple MTL + interpretative texts: saturated fat, sugar, sodium, simple MTL + interpretative texts: saturated	N

37	16	Antúnez 2013	Uruguay	English	47	Y	N	no label	choice conjoint task	high	>18	adults or most adults	0.58	mixed	ns	ns	ns	ns	Bread & bakery products	single	N		package	N	N	N	N	N	N	N	N	N	Y	Y	N	Y (detailed MTL)	N
41	17	Goodman 2013	Canada	English	247	Y	N	no label	randomized controlled trial	high	>18	adults or most adults	0.535	mixed	white	high	mix	ns	snack foods	single	N		package	N	N	N	N	N	N	N	N	N	Y	Y	N	Y (simple TLL + interpretative texts: sodium only, detailed TLL + interpretative	N
44	18	Mejean 2013	France	English	39370	N	N	summary TLS	cross-sectional	high	adults	adults or most adults	0.765	mostly female	ns	high	ns	mix	Convenient foods	single	N		package	N	N	N	N	N	N	N	N	N	Y	Y	Y (based on ''SAIN LIM'' system)	Y (simple MTL: saturated fat, sugar	N
48	19	Savoie 2013	Canada	English	2200	N	N	no label	nonequivalent control group design: pretest-posttest design	high	>18	adults or most adults	0.65	mostly female	white	high	mix	ns	eggs, Cereal and cereal products, Bread & bakery products, Dairy, Non-alcoholic beverages, snack foods, Confectionery, Fruit and Vegetables, Meat and meat products, Fish and	multiple	N		package	N	N	N	N	N	N	N	N	N	Y	Y	N	Y (detailed MTL)	N
49	20	Sonnenberg 2013	US	English	389	N	N	no label	nonequivalent control group design: posttest design	high	most >40	adults or most adults	0.59	mixed	white	ns	ns	ns	campus/workplace cafeteria meal	multiple	Y	cafeteria	package	N	N	N	N	N	N	N	N	N	Y	Y	Y (base on 3 positive criteria and 2 negative criteria)	Y (detailed MTL)	N
50	21	Ares 2014	Uruguay	English	71	Y	N	no label	choice conjoint task	high	25.3	adults or most adults	0.68	mostly female	ns	ns	ns	ns	Dairy	single	N		package	N	N	N	N	N	N	N	N	N	Y	Y	N	Y (detailed MTL: sugar, fat, saturated	N
56	22	Emrich 2014	Canada	English	1188	Y	N	no label	randomized controlled trial	high	>20	adults or most adults	0.654	mostly female	ns	high	mix	ns	Convenient foods, Cereal and cereal products	multiple	N		package	N	N	N	N	N	N	N	N	N	Y	Y	N	Y (detailed MTL)	N
57	23	Koenigstorfer 2014	Germany	English	120	N	N	NFt	randomized controlled trial	high	37.8	adults or most adults	0.64	mostly female	ns	ns	ns	ns	Cereal and cereal products, Bread Bread & bakery products, snack foods, Confectionery, Fruit and Vegetabl	multiple	N		package	N	N	N	N	N	N	N	N	N	Y	Y	N	Y (detailed MTL)	N
58	24	Maubach 2014	New Zealand	English	768	Y	N	no label	choice conjoint task	high	49.6	adults or most adults	0.7	mostly female	white	low	ns	ns	Cereal and cereal products	single	N		package	N	N	N	N	N	N	N	N	N	Y	Y	N	Y (detailed MTL)	N
62	25	Van Herpen 2014a	Germany	English	333	N	N	NFt	randomized controlled trial	high	28	adults or most adults	0.52	mixed	ns	high	ns	students (54%, n = 288), faculty staff, and close dependents	Cereal and cereal products, snack foods, Bread & bakery products	multiple	N		package	N	N	N	N	N	N	N	N	N	Y	Y	N	Y (simple MTL)	N
62	25	Van Herpen 2014b	Germany	English	44	N	N	no label	randomized controlled trial	high	21	adults or most adults	0.77	mostly female	ns	high	ns	undergraduate students	Non-alcoholic beverages, Convenient foods, Sauces and spreads	multiple	N		package	N	N	N	N	N	N	N	N	N	Y	Y	N	Y (detailed MTL)	N

64	26	Watson 2014	Australia	English	2175	Y	N	no label	randomized controlled trial	high	⩾18	adults or most adults	0.65	mostly female	ns	low	mix	ns	Cereal and cereal products, Meat and meat products, snack foods, Dairy, Bread & bakery	multiple	N		package	N	N	N	N	N	N	N	N	N	Y	Y	N	Y (detailed MTL+interpretive text, detailed MTL+ oveall rating)	N
67	27	Ducrot 2015	France	English	14230	N	N	no label	cross-sectional	high	⩾18	adults or most adults	0.7814	mostly female	ns	low	mix	ns	Convenient foods	single	N		package	N	N	N	N	N	N	N	N	N	Y	Y	N	Y (simple MTL)	Y
69	28	Hamlin 2015	New Zealand	English	250	N	N	no label	randomized controlled trial	high	18-25	adults or most adults	0.62	mostly female	ns	high	ns	university students	Cereal and cereal products	single	N		package	N	N	N	N	N	N	N	N	N	Y	Y	N	Y (detailed MTL + interpretative texts: sugar, fat	N
72	29	Mawad 2015	Uruguay	English	133	Y	N	no label	choice conjoint task	high	23.3	adults or most adults	0.66	mostly female	ns	ns	ns	ns	Dairy	single	N		package	N	N	N	N	N	N	N	N	N	Y	Y	N	Y (detailed MTL: sugar, fat, salt)	N
74	30	Pauline 2015	France	English	13578	N	N	no label	cross-sectional	high	⩾18	adults or most adults	0.524	mixed	ns	low (<50% college in parents)		mix	Cereal and cereal products, Fish and fish products, snack foods, Dairy, Convenie	multiple	N		package	N	N	N	N	N	N	N	N	N	Y	Y	N	Y (simple MTL)	Y
77	31	Siegrist 2015	Switzerland	English	61	N	N	NFt	randomized controlled trial	high	35	adults or most adults	0.69	mostly female	ns	low (<50% college in parents)			Cereal and cereal products, snack foods, Dairy, Non-alcoholic beverage	multiple	N		package	N	N	N	N	N	N	N	N	N	Y	Y	N	Y (detailed MTL)	N
79	32	Acton 2016	Canada	English	670	Y	N	no label	randomized controlled trial	high	16–24 (50% adults)	mix	0.501	mixed	white	ns	ns	ns	NS	NS	N		package	N	N	N	N	N	N	N	N	N	Y	Y	N	Y (calorie only, calorie only + interpreta	N
81	33	Bollard 2016	New Zealand	English	604	N	N	no label	randomized controlled trial	high	18 (<50% adults)	mix	0.51	mixed	white	low (<50% college in parents)	mix	ns	Non-alcoholic beverages	single	N		package	Y	Y	Y	Y	Y	N	Y	N	Y	N	N	N	N	N
82	34	Crosetto 2016b	France	English	174	N	Y	simple TLS	choice conjoint task	high	ns	ns	ns	ns	ns	ns	ns	ns	restaurant meals meal	multiple	N		package	N	N	N	N	N	N	N	N	N	Y	Y	N	Y (simple MTL [saturated fat, sugar, salt], simple MTL [saturated fat, sugar, salt, vitamins, fiber and calcium], color-coded GDA [saturated fat, sugar, salt], color-coded GDA [saturate	N
84	35	Ducrot 2016	France	English	7103	N	N	no label	randomized controlled trial	high	>18	adults or most adults	0.81	mostly female	ns	low (<50% college in parents)	mix		general food and beverage products	multiple	N		package	N	N	N	N	N	N	N	N	N	Y	Y	N	Y (simple MTL)	Y
92	36	Talati 2016	Australia	English	1984	N	N	no label	choice conjoint task	high	>10 (74% adults)	adults or most adults	0.5	mixed	ns				snack foods, Convenient foods and	multiple	N		package	N	N	N	N	N	N	N	N	N	Y	Y	N	Y (detailed MTL)	N

97	37	VanEpps 2016b	US	English	1842	N	N	no label	randomized controlled trial	high	15	children or adolescent	0.503	mixed	white	low (100% no college, parents <50% college	ns	ns	Non-alcoholic beverages	single	N		package	Y	N	Y	N	Y	Y	N	N	Y	N	N	N	N	N
99	38	Arrua 2017a	Uruguay	English	442	N	N	no label	randomized controlled trial	high	8--13	children or adolescent	0.5	mixed	ns	low (primary school students)	ns	ns	Non-alcoholic beverages, snack foods	multiple	N		package	Y	Y	N	Y	N	N	Y	N	N	Y	Y	N	Y (detailed MTL)	N
100	39	Arrúa 2017b	Uruguay	English	32	N	N	TLS	choice conjoint task	high	>18	adults or most adults	ns	mostly female	ns	ns	ns	ns	Meat and meat products, Convenient foods	multiple	N		package	Y	Y	N	Y	N	N	Y	N	N	N	N	N	N	N
100	39	Arrúa 2017c	Uruguay	English	260	N	N	TLS	randomized controlled trial	high	>18	adults or most adults	0.67	mostly female	ns	low (primary school students)	ns	ns	Bread & bakery products, Cereal and cereal products, snack foods, Convenient foods	multiple	N		package	Y	Y	N	Y	N	N	Y	N	N	N	N	N	N	N
102	40	Boncinelli 2017	Italy	English	96	N	N	no label	nonequivalent control group design: posttest design	high	ns	ns	ns	mixed	ns	ns	ns	ns	Confectionery	single	N		package	Y	N	Y	Y	N	Y	N	N	N	N	N	N	N	N
113	41	Julia 2017	France	English	21702	N	N	no label	cross-sectional	high	>18	adults or most adults	0.7342	mostly female	ns	high (>50% college)	mix	ns	general food and beverage products	multiple	N		package	N	N	N	N	N	N	N	N	N	Y	Y	N	Y (detailed MTL)	Y
115	42	Neal 2017	Australia	English	944	N	N	NFt	randomized controlled trial	high	38	adults or most adults	0.84	mostly female	ns	high (>50% college)	mix	ns	general food and beverage products	multiple	N		package	Y	N	Y	Y	N	N	Y	N	N	Y	Y	N	Y (detailed MTL)	N
116	43	Ni Mhurchu 2017	New Zealand	English	942	N	N	NFt	randomized controlled trial	high	33	adults or most adults	0.89	mostly female	mix	high (>50% college)	mix	ns	general food and beverage products	multiple	N		package	N	N	N	N	N	N	N	N	N	Y	Y	N	Y (detailed MTL)	N
121	44	Talati 2017	Australia	English	2058	Y	N	no label	choice conjoint task	high	>10 (75% adults)	adults or most adults	0.5	mixed	ns				Dairy, snack foods, Convenient foods	multiple	N		package	N	N	N	N	N	N	N	N	N	Y	Y	N	Y (detailed MTL)	N
122	45	Yoo 2017	Ecuador	English	646	N	N	no label	choice conjoint task	upper-middle	7--12	children or adolescent	0.44	mixed	ns	ns	mix	ns	Dairy	single	N		package	N	N	N	N	N	N	N	N	N	Y	Y	N	Y (detailed MTL + interpretative texts: sugar	N
123	46	Acton 2018a	Canada	English	675	N	N	no label	randomized controlled trial	high	>16 (84.9% adults)	adults or most adults	0.539	mixed	white	ns	ns	ns	Non-alcoholic beverages	single	N		package	Y	N	Y	Y	Y	N	Y	N	Y	N	N	N	N	N
126	47	Acton 2018b	Canada	English	358	Y	N	no label	randomized controlled trial	high	>16 (84.9% adults)	adults or most adults	0.539	mixed	white	ns	ns	ns	Non-alcoholic beverages, Dairy	multiple	N		package	N	N	N	N	N	N	N	N	N	Y	Y	N	Y (sugar only)	N
127	48	Ares 2018a	Uruguay	English	306	N	N	no label	choice conjoint task	high	>18	adults or most adults	0.63	mostly female	ns				Bread & bakery products	multiple	N		package	Y	Y	N	Y	N	N	Y	N	N	N	N	N	N	N
128	49	Ares 2018b	Uruguay	English	395	N	N	no label	one-group design: pretest-posttest design	high	>18	adults or most adults	0.72	mostly female	ns				Fruit and vegetables, snack foods, Meat and meat products, Bread & bakery products, Non-alcoholic	multiple	N		package	Y	Y	N	Y	N	N	Y	N	N	N	N	N	N	N
130	50	Ares 2018c	Uruguay	English	112	N	N	NS	choice conjoint task	high	>18	adults or most adults	0.7	mostly female	ns				Diary, Bread & bakery products, Cereal and cereal products, Sauces and spreads, snack foods, Non-alcoholic beverage	multiple	N		package	Y	Y	N	Y	N	N	Y	N	N	Y	N	N	N	Y

130	50	Ares 2018d	Uruguay	English	681	N	N	no label	randomized controlled trial	high	>18	adults or most adults	0.67	mostly female	ns				Fruit and vegetables, Bread & bakery products, Cereal and cereal products, Dairy, Non-alcoholic beverages, snack foods, Sauces	multiple	N		package	Y	Y	N	Y	N	N	Y	N	N	Y	N	N	N	Y
132	51	Billich 2018	Australia	English	581	N	N	no label	randomized controlled trial	high	>18	adults or most adults	0.5	mixed	ns				Non-alcoholic beverages	single	N		package	Y	N	Y	N	Y	Y	Y	N	Y	N	N	N	N	N
136	52	David 2018	Brazil	English	98	N	N	no label	choice conjoint task	upper-middle	20.2	adults or most adults	0.53	mixed	ns	high (100% college)	ns	university students	Non-alcoholic beverages, Confectionery, snack foods	multiple	N		package	Y	N	Y	Y	Y	Y	Y	N	Y	N	N	N	N	N
137	53	Donnelly 2018b	US	English	sales data	N	N	no label	Interrupted Time-Series Design	high									Non-alcoholic beverages	single	Y	cafeteria	package	Y	N	Y	N	Y	Y	Y	N	Y	N	N	N	N	N
137	53	Donnelly 2018a	US	English	202	N	N	no label	randomized controlled trial	high	33.86	adults or most adults	0.488	mixed	white	high (82.6% attended at least some college)	mix	ns	Non-alcoholic beverages	single	N		package	Y	N	Y	N	Y	Y	Y	N	Y	N	N	N	N	N
138	54	Egnell 2018a	France	English	3751	N	N	no label	choice conjoint task	high	49.4	adults or most adults	0.814	mostly female	ns	high (>50% college)	high (>50% with highest family incomes)	ns	Fish and fish products, Bread & bakery products, Cereal and cereal products, Convenie	multiple	N		package	N	N	N	N	N	N	N	N	N	Y	Y	N	Y (detailed MTL)	Y
139	55	Egnell 2018b	France	English	25772	N	N	no label	choice conjoint task	high	56.05	adults or most adults	0.73	mostly female	ns	high (>50% post-secondary)	mix	mix	Bread & bakery products, Dairy, Sauces and spreads	multiple	N		package	N	N	N	N	N	N	N	N	N	Y	Y	N	Y (detailed MTL)	Y
144	56	Goodman 2018	Canada, US, Australia, and UK	English	11617	N	N	no label	randomized controlled trial	high	>18	adults or most adults	0.529	mixed	ns	low (<50% high-level educatio	ns	ns	Cereal and cereal products	single	N		package	Y	N	Y	Y	N	N	Y	N	N	N	N	N	N	N
145	57	Gorski Findling 2018	US	English	628	N	N	no label	randomized controlled trial	high	43.4	adults or most adults	0.54	mixed	white	low (<50% college)	mix	ns	Dairy, Cereal and cereal products, Convenient foods, Non-alcoholic beverages	multiple	N		package	N	N	N	N	N	N	N	N	N	Y	Y	N	Y (simple MTL: calorie only; simple MTL + interpretative texts: sugar, fat, salt)	N
149	58	Khandpur 2018	Brazil	English	1607	N	N	TLS	randomized controlled trial	upper-middle	39.2	adults or most adults	0.5246	mixed	ns				Bread & bakery products, Cereal and cereal products, Convenient foods, snack foods, Non-alcoholic beverages	multiple	N		package	Y	N	Y	Y	N	N	Y	N	N	Y	Y	N	Y (detailed MTL)	N

152	59	Lima 2018	Brazil	English	396	N	N	TLS	randomized controlled trial	upper-middle	>6 (46.8% adults)	mix	0.648	mostly female	ns				Cereal and cereal products, Bread & bakery products, Confectionery, snack foods, Dairy, Non-alcoholic beverage	multiple	N		package	Y	Y	N	Y	N	N	Y	N	N	Y	Y	N	Y (simple MTL + interpretative text: calories, sugar, saturated fat and sodium)	N
154	60	Machin 2018a	Uruguay	English	437	N	N	no label	randomized controlled trial	high	>18	adults or most adults	0.75	mostly female	ns	low (<50% college)	ns	ns	general food and beverage products	multiple	N		package	Y	Y	N	Y	N	N	Y	N	N	Y	Y	N	Y (detailed MTL + interpretative: sugar, fat, salt)	N
155	61	Machin 2018c	Uruguay	English	216	N	N	TLS	choice conjoint task	high	>18	adults or most adults	0.75	mostly female	ns	ns	ns	ns	NS	NS	N		package	Y	Y	N	Y	N	N	Y	N	N	Y	Y	N	Y (simple MTL + interpretative texts: sugar	N
155	61	Machin 2018b	Uruguay	English	1003	N	N	TLS	randomized controlled trial	high	>18	adults or most adults	0.75	mostly female	ns	ns	ns	ns	Bread & bakery products, Meat and meat products, Dairy	multiple	N		package	Y	Y	N	Y	N	N	Y	N	N	Y	Y	N	Y (simple MTL + interpretative texts: sugar, fat, salt)	N
156	62	Machin 2018d	Uruguay	English	1182	N	N	no label	randomized controlled trial	high	>18	adults or most adults	0.91	mostly female	ns	low (<50% college)	ns	ns	general food and beverage products	multiple	N		package	Y	Y	N	Y	N	N	Y	N	N	Y	Y	N	Y (detailed MTL + interpretative texts: sugar, saturated	N
158	63	Mantzari 2018	UK	English	997	N	N	no label	randomized controlled trial	high	43.8	adults or most adults	0.481	mixed	white	high (>50% college)	ns	ns	Non-alcoholic beverages	single	N		package	Y	N	Y	Y	Y	N	Y	N	Y	N	N	N	N	N
160	64	Mazza 2018	US	English	sales data	N	N	no label	Interrupted Time-Series Design	high									campus/workplace cafeteria meal	multiple	Y	cafeteria	package	N	N	N	N	N	N	N	N	N	Y	Y	Y (base on calories, fat, saturated fat, trans fat, cholesterol, sodium, sugars)	N	N
161	65	Moran 2018	US	English	1970	N	N	no label	randomized controlled trial	high	36.2	adults or most adults	0.7	mostly female	white	low (<50% college)	mix	ns	Non-alcoholic beverages	single	N		package	Y	N	Y	N	Y	Y	N	N	Y	N	N	N	N	N
172	66	Talati 2018a	Australia	English	690	Y	N	no label	randomized controlled trial	high	>10 (72% adults)	adults or most adults	0.51	mixed	ns				Cereal and cereal products, Convenient foods, snack foods, Dairy	multiple	N		package	N	N	N	N	N	N	N	N	N	Y	Y	N	Y (detailed MTL)	N
173	67	Talati 2018b	Australia	English	1505	N	N	no label	choice conjoint task	high	>18	adults or most adults	0.5	mixed	ns				Cereal and cereal products, Convenient foods, snack foods, Dairy	multiple	N		package	N	N	N	N	N	N	N	N	N	Y	Y	N	Y (detailed MTL)	N
177	68	Acton 2019	Canada	English	2866	N	N	no label	randomized controlled trial	high	>13 (84.7% adults)	adults or most adults	0.56	mixed	mix	high (>50% college)	mix	ns	Non-acoholic beverages, snack foods	multiple	N		package	Y	N	Y	Y	N	N	N	N	N	Y	Y	N	Y (detailed MTL)	Y
178	69	Alonso-Dos-Santos 2019	Chile	English	337	N	N	NFt	randomized controlled trial	high	23.4	adults or most adults	0.5	mixed	ns	ns	ns	ns	Bread and bakery products, snack foods, Dairy	multiple	N		package	Y	Y	N	Y	N	N	Y	N	N	N	N	N	N	N
179	70	Ang 2019	Singapore	English	512	Y	N	no label	randomized controlled trial	high	38.1	adults or most adults	0.467	mixed	Chinese	ns	ns	ns	general food and beverage products	multiple	N		package	Y	Y	Y	Y	Y	N	N	N	Y	N	N	N	N	N

185	71	Egnell 2019a	France	English	352	Y	N	no label	randomized controlled trial	high	>18	adults or most adults	0.797	mostly female	ns	high (>50% college)	mix	mix (>50% intermediate profession/office staff)	general food and beverage products	multiple	N		package	N	N	N	N	N	N	N	N	N	Y	Y	N	Y (detailed MTL)	Y
188	72	Galan 2019	Spain	Spanish	1000	N	N	no label	randomized controlled trial	high	>18	adults or most adults	0.5	mixed	ns	low (<50% college)	mix	ns	Cereal and cereal products, Bread & bakery products, Convenience	multiple	N		package	Y	Y	N	Y	N	N	Y	N	N	Y	Y	N	Y (detailed MTL)	Y
189	73	Grummon 2019a	US	English	1360	N	N	no label	randomized controlled trial	high	37.4	adults or most adults	0.47	mixed	white	high (>50% college)	mix	ns	Non-alcoholic beverages	single	N		package	Y	Y	Y	Y	Y	N	Y	N	Y	N	N	N	N	N
191	74	Hayward 2019	US	English	681	N	N	no label	randomized controlled trial	high	41.38	adults or most adults	0.49	mixed	white	high (>50% college)	ns	ns	Non-alcoholic beverages	single	N		package	Y	N	Y	N	Y	N	Y	N	Y	N	N	N	N	N
193	75	Karamanos 2019	Canada	English	242	N	N	NFt	cross-sectional	high	>25	adults or most adults	0.5372	mixed	ns	high (>50% college)	ns	ns	NS	NS	N		package	N	N	N	N	N	N	N	N	N	Y	Y	N	Y (detailed MTL)	N
196	76	Lima 2019	Brazil	English	800	Y	N	TLS	randomized controlled trial	upper-middle	>6 (50% adults)	mix	0.59	mixed	ns	ns	ns	ns	Sugars, honey and related products, Dairy	multiple	N		package	Y	Y	N	Y	N	N	Y	N	N	Y	Y	N	Y (detailed MTL + interpretative texts: sugar	N
198	77	Machín 2019	Uruguay	English	199	N	N	no label	randomized controlled trial	high	>18	adults or most adults	0.66	mostly female	ns	high (>50% college)	ns	ns	snack foods	single	N		package	Y	Y	N	Y	N	N	Y	N	N	N	N	N	N	N
199	78	Marette 2019	France	English	86	N	N	no label	one-group design: pretest-posttest design	high	38.45	adults or most adults	0.744	mostly female	ns	ns	low		Cereal and cereal products	single	N		package	N	N	N	N	N	N	N	N	N	Y	N	N	N	Y
206	79	Popova 2019	US	English	180	N	N	no label	randomized controlled trial	high	25.6	adults or most adults	0.739	mostly female	mix	high (>50% college)	mix	ns	Non-alcoholic beverages	single	N		package	Y	N	Y	N	Y	Y	N	N	Y	N	N	N	N	N
208	80	Rosenblatt 2019a	Australia	English	100	N	N	textual warning	randomized controlled trial	high	27.59	adults or most adults	0.74	mostly female	ns	ns	ns	ns	snack foods	single	N		package	Y	Y	Y	Y	Y	Y	Y	Y	Y	N	N	N	N	N
208	80	Rosenblatt 2019b	Australia	English	43	N	N	positively framed warning	randomized controlled trial	high	24.85	adults or most adults	0.58	mixed	ns	ns	ns	ns	snack foods	single	N		package	Y	Y	Y	Y	Y	Y	Y	Y	Y	N	N	N	N	N
210	81	Schnettler 2019a	Chile	English	245	N	N	no label	choice conjoint task	high	39.4	adults or most adults	0.77	mostly female	ns				Meat and meat products	single	N		package	Y	Y	N	Y	N	N	Y	N	N	N	N	N	N	N
210	81	Schnettler 2019b	Chile	English	249	N	N	no label	choice conjoint task	high	40.4	adults or most adults	0.746	mostly female	ns				Meat and meat products	single	N		package	Y	Y	N	Y	N	N	Y	N	N	N	N	N	N	N
211	82	Schnettler 2019	Chile	English	548	N	N	no label	one-group design: pretest-posttest design	high	40	adults or most adults	0.724	mostly female	ns				Meat and meat products	single	N		package	Y	Y	N	Y	N	N	Y	N	N	N	N	N	N	N
216	83	Velasco Vizcaíno 2019d	Ecuador	English	133	N	N	NFt	nonequivalent control group design: posttest design	upper-middle	24.83	adults or most adults	0.38	mostly male	ns	ns	ns	ns	Dairy	single	N		package	N	N	N	N	N	N	N	N	N	Y	Y	N	Y (simple MTL + interpretative texts: sugar	N
216	83	Velasco Vizcaíno 2019a	Ecuador	English	837	N	N	no label	randomized controlled trial	upper-middle	28.79	adults or most adults	0.53	mixed	ns	ns	ns	ns	Meat and meat products	single	N		package	N	N	N	N	N	N	N	N	N	Y	Y	N	Y (simple MTL + interpretative texts: sugar	N
216	83	Velasco Vizcaíno 2019b	Ecuador	English	181	N	N	no label	randomized controlled trial	upper-middle	37.24	adults or most adults	0.467	mixed	ns	ns	ns	ns	snack foods	single	N		package	N	N	N	N	N	N	N	N	N	Y	Y	N	Y (simple MTL + interpretative texts: sugar	N
216	83	Velasco Vizcaíno 2019c	Ecuador	English	201	N	N	no label	randomized controlled trial	upper-middle	37.85	adults or most adults	0.711	mostly female	ns	ns	ns	ns	Confectionery	single	N		package	N	N	N	N	N	N	N	N	N	Y	Y	N	Y (simple MTL + interpretative texts: sugar	N
218	84	Nobrega 2020	Brazil	English	820	N	N	no label	choice conjoint task	upper-middle	>18	adults or most adults	0.52	mixed	ns	low (<50% college)	mix	ns	Dairy, Non-acoholic beverages, Bread & bakery products, snack foods	multiple	N		package	Y	Y	N	Y	N	N	Y	N	N	N	N	N	N	N

229	85	Bix 2015	US	English	55	Y	N	no label	choice conjoint task	high	36.6	adults or most adults	0.6	mixed	mix	low (<50% college)	low (>50% low household income)	ns	Cereal and cereal products, snack foods	multiple	N		package	N	N	N	N	N	N	N	N	N	Y	Y	N	Y (detailed MTL + facial icons: total fat, saturated fat, sugar	N
231	86	Hobin 2015	Canada	English	1005	Y	N	no label	randomized controlled trial	high	16-24 (50% 16-18)	mix	0.5	mixed	white	low (<50% college)	ns	ns	snack foods	single	N		package	N	N	N	N	N	N	N	N	N	Y	Y	N	Y (detailed MTL: calories, total fat, saturated fat, sodium)	N
237	87	Egnell 2019b	France	English	1215	N	N	no label	randomized controlled trial	high	20.4	adults or most adults	0.73	mostly female	ns	high (>51% college)	ns	undergraduate students	general food and beverage products	multiple	N		package	N	N	N	N	N	N	N	N	N	Y	N	N	N	Y
242	88	Poquet 2019	France	English	95	N	N	no label	one-group design: pretest-posttest design	high	9.38	adults or most adults	0.568	mixed	ns	low (primary school)	mix	primary school student	snack foods	multiple	N		package	N	N	N	N	N	N	N	N	N	Y	N	N	N	Y
244	89	Trudel 2015a	US	English	123	Y	N	no label	randomized controlled trial	high	19.93	adults or most adults	0.54	mixed	ns	high (>50% college)	ns	undergraduate students	Convenient foods	single	N		package	N	N	N	N	N	N	N	N	N	Y	Y	N	Y (detailed MTL: total fat, saturated fat, trans fatty acids, cholesterol, sodium, carbohydrates, sugars,	N
244	89	Trudel 2015b	US	English	227	Y	N	no label	randomized controlled trial	high	>18	adults or most adults	0.57	mixed	ns	high (>51% college)	ns	undergraduate students	Convenient foods	single	N		package	N	N	N	N	N	N	N	N	N	Y	Y	N	Y (detailed MTL: total fat, saturated fat, trans fatty acids, cholesterol, sodium, carbohydrates, sugars,	N
245	90	Crockett 2014	UK	English	184	N	N	no label	randomized controlled trial	high	>18	adults or most adults	0.64	mostly female	ns				snack foods	single	Y	cinema	package	N	N	N	N	N	N	N	N	N	Y	Y	N	Y (fat only: only green, only red)	N
252	91	Egnell 2019c	Germany	English	600	N	N	no label	randomized controlled trial	high	>18	adults or most adults	0.5	mixed	ns	low (<50% college)	mix (33% high, 33% low)	ns	Cereal and cereal products, Bread & bakery products, Convenient foods	multiple	N		package	Y	Y	N	Y	N	N	Y	N	N	Y	Y	N	Y (detailed MTL)	Y
253	92	Finkelstein 2019	Singapore	English	154	Y	N	no label	randomized controlled trial	high	34.4	adults or most adults	0.6883	mostly female	Chinese	high (>50% college)	mix	ns	general food and beverage products	multiple	N		package	N	N	N	N	N	N	N	N	N	Y	Y	N	Y (detailed MTL)	Y
255	93	Grummon 2019b	US	English	400	N	N	no label	randomized controlled trial	high	29	adults or most adults	0.6	mixed	mix	high (>50% college)	mix	ns	Non-alcoholic beverages	single	N		package	Y	N	Y	N	Y	N	Y	N	Y	N	N	N	N	N
261	94	Nieto 2019	US and Mexico	English	7159	N	N	NFt	cross-sectional	high & upper-middle	>18	adults or most adults	0.5	mixed	mix	high (>50% college)	mix	mix (>50% worker and student)	NS	NS	N		package	Y	Y	N	Y	N	N	Y	N	N	Y	Y	N	Y (detailed MTL)	N
264	95	Talati 2019a	12 countries (Argentina, Australia, Bulgaria, Canada, Denmark, France, Germany, Mexico, Singapore, Spain, the UK and the	English	6660	N	N	no label	randomized controlled trial	high & upper-middle	>18	adults or most adults	0.5	mixed	ns	ns	ns	ns	Cereal and cereal products, Bread & bakery products, Convenient foods	multiple	N		package	Y	Y	N	Y	N	N	Y	N	N	Y	Y	N	Y (detailed MTL)	Y

265	96	Talati 2019b	12 countries (Argentina, Australia, Bulgaria, Canada, Denmark, France, Germany, Mexico, Singapore, Spain, the UK and the	English	7087	N	N	TLS	randomized controlled trial	high & upper-middle	>18	adults or most adults	0.5	mixed	ns	ns	mix (33% high, 33% low)	ns	NS	NS	N		package	Y	Y	N	Y	N	N	Y	N	N	Y	Y	N	Y (detailed MTL)	Y
268	97	Mantzari 2020	UK	English	270	N	N	no label	randomized controlled trial	high	40	adults or most adults	0.56	mixed	white				Non-alcoholic beverages	single	N		package	Y	N	Y	N	Y	N	Y	N	Y	N	N	N	N	N
271	98	Anabtawi 2020	Peru	English	100	N	N	no label	randomized controlled trial	upper-middle	19.81	adults or most adults	0.53	mixed	ns	high (100% college)	ns	ns	Non-alcoholic beverages & snack	multiple	N		package	N	N	N	N	N	N	N	N	N	Y	Y	N	Y (detailed MTL)	N
275	99	Egnell 2020a	Switzerland	English	600	N	N	TLS	randomized controlled trial	high	>18	adults or most adults	0.49	mixed	ns	low	mixed	ns	Cereal and cereal products, Bread & bakery products	multiple	N		package	Y	Y	N	Y	N	N	Y	N	N	Y	Y	N	Y (detailed MTL)	Y
276	100	Franco-Arellano 2020	Canada	English	1498	Y	N	no label	randomized controlled trial	high	>18	adults or most adults	0.52	mixed	white	high	mixed	ns	Non-alcoholic beverages & snack	multiple	N		package	Y	N	Y	Y	N	N	Y	N	N	Y	Y	N	Y (simplified MTL)	N
279	101	Gabor 2019	Spain	English	50	N	N	NS	randomized controlled trial	high	21.3	adults or most adults	0.64	mostly female	ns	ns	ns	ns	Confectionery	single	N		package	N	N	N	N	N	N	N	N	N	Y	Y	N	Y (detailed MTL)	Y
280	102	Hagmann 2020	Switzerland	English	799	N	N	no label	randomized controlled trial	high	48.7	adults or most adults	0.54	mixed	ns	low	ns	ns	snack foods	single	N		package	N	N	N	N	N	N	N	N	N	Y	Y	N	Y (detailed MTL)	Y
282	103	Hall 2020a	US	English	1352	N	N	textual HW	randomized controlled trial	high	37	adults or most adults	0.47	mixed	white	high	mixed	ns	Non-alcoholic beverages	single	N		package	Y	N	Y	N	Y	Y	Y	N	Y	N	N	N	N	N
283	104	Hall 2020b	US	English	2139	N	N	no label	randomized controlled trial	high	43	adults or most adults	0.45	mixed	white	low	mixed	ns	Non-alcoholic beverages	single	N		package	Y	N	Y	N	Y	Y	N	N	Y	N	N	N	N	N
283	104	Hall 2020c	US	English	670	N	N	no label	randomized controlled trial	high	37	adults or most adults	0.49	mixed	white	low	mixed	ns	Non-alcoholic beverages	single	N		package	Y	N	Y	N	Y	Y	N	N	Y	N	N	N	N	N
283	104	Hall 2020d	US	English	1001	N	N	no label	randomized controlled trial	high	39	adults or most adults	0.46	mixed	white	high	mixed	ns	Non-alcoholic beverages	single	N		package	Y	N	Y	N	Y	Y	N	N	Y	N	N	N	N	N
284	105	Hernández-Nava 2020	Mexico	Spanish	600	N	N	no label	randomized controlled trial	upper-middle	>18	adults or most adults	0.5	mixed	ns	high	mixed	ns	Cereal and cereal products, Bread and bakery products	multiple	N		package	Y	Y	N	Y	N	N	Y	N	N	Y	Y	N	Y (detailed MTL)	Y
285	106	Jáuregui 2020	Mexico	English	1469	N	N	TLS	randomized controlled trial	upper-middle	>21	adults or most adults	0.43	mixed	ns	low	ns	mixed	Cereal and cereal products, Bread and bakery products, snack foods, non-alcoholic beverage, dairy, convenie	multiple	N		package	Y	Y	N	Y	N	N	Y	N	N	Y	Y	N	Y (detailed MTL)	N
289	107	Retno 2019	Indonesia	English	41	Y	N	no label	nonequivalent control group design: posttest design	lower-middle	15-17	children or adolescent	1	mostly female	ns	low	ns	ns	Bread and bakery products, dairy	multiple	N		package	N	N	N	N	N	N	N	N	N	Y	Y	N	Y (detailed MTL)	N
291	108	Taillie 2020a	Chile	English	2383	Y	N	no label	one-group design: pretest-posttest design	high	ns	ns		mixed	ns	low	mixed		Non-alcoholic beverages	single	Y	supermarkets and gocery stores	package	Y	Y	N	Y	N	N	Y	N	N	N	N	N	N	N
292	109	Uribe 2020	Chile	English	320	N	N	no label	randomized controlled trial	high	ns	ns	ns	ns	ns	ns	ns	ns	Cereal and cereal products, snack, condiments	multiple	N		package	Y	Y	N	Y	N	N	Y	N	N	N	N	N	N	N

293	110	Vandevijvere 2020	Belgium	English	600	N	N	NS	randomized controlled trial	high	>18	adults or most adults	0.5	mixed	ns	high	mixed	ns	Cereal and cereal products, Bread and bakery products	multiple	N		package	Y	Y	N	Y	N	N	Y	N	N	Y	Y	N	Y (detailed MTL)	Y
294	111	Vargas-Meza 2019	Mexico	English	1408	N	N	TLS	randomized controlled trial	upper-middle	>18	adults or most adults	0.57	mixed	ns	low	mixed	ns	Cereal and cereal products, snack foods, non-alcoholic beverage, dairy, convenie	multiple	N		package	Y	Y	N	Y	N	N	Y	N	N	Y	Y	N	Y (detailed MTL)	N
298	112	Julia 2016	France	English	901	N	N	no label	nonequivalent control group design: posttest design	high	>18	adults or most adults	0.71	mostly female	ns	ns	ns	mixed	snacks and cereals	multiple	N		package	N	N	N	N	N	N	N	N	N	Y	N	N	N	Y
299	113	Roberto 2016	US	English	1984	N	N	no label	randomized controlled trial	high	36.2	adults or most adults	0.7	mostly female	white	high	mixed	ns	Non-alcoholic beverages	single	N		package	Y	N	Y	N	Y	Y	N	N	Y	N	N	N	N	N
300	114	Andreeva 2020	Bulgaria	English	808	N	N	NFt	randomized controlled trial	upper-middle	39.2	adults or most adults	0.499	mixed	ns	high	mixed	ns	Bread & bakery products, Cereal and cereal products	multiple	N		package	Y	Y	N	Y	N	N	Y	N	N	Y	Y	N	Y (detailed MTL)	Y
301	115	Blitstein 2020	US	English	724	Y	Y	no label	randomized controlled trial	high	34.5	adults or most adults		mostly female	white	low	mixed	ns	Bread & bakery products, Cereal and cereal products, snack foods	multiple	N		package	N	N	N	N	N	N	N	N	N	N	Y	N	Y (detailed MTL)	N
302	116	Clarke 2020	UK	English	4134	N	N	no label	randomized controlled trial	high	46.5-47.8	adults or most adults	0.47-0.49	mixed	white	high	ns	ns	snack foods	single	N		package	Y	N	Y	N	Y	Y	Y	N	Y	N	N	N	N	N
303	117	Fialon 2020	Italy	English	826	N	N	NFt	randomized controlled trial	high	>18	adults or most adults	0.501	mixed	ns	high	ns	ns	Bread & bakery products, Cereal and cereal products	multiple	N		package	Y	Y	N	Y	N	N	Y	N	N	Y	Y	N	Y (detailed MTL)	Y
304	118	Hamlin 2020	New Zealand	English	240	N	N	no label	choice conjoint task	high	16-18	children or adolescent	0.58	mixed	ns	low	ns	ns	Cereal and cereal products	single	N		package	Y	Y	N	Y	N	N	N	N	N	N	N	N	N	N
306	119	Panzone 2020	UK	English	756	N	N	no label	randomized controlled trial	high	48.4	adults or most adults	0.53	mixed	ns	ns	ns	ns	Fruit and Vegetables, diary	multiple	N		package	N	N	N	N	N	N	N	N	N	Y	Y	N	Y	N
307	120	Santos 2020	Portugal	English	357	N	N	no label	randomized controlled trial	high	40.6	adults or most adults	0.54	mixed	ns	ns	ns	ns	Cereal and cereal products, Dairy, Fish and fish product	multiple	N		package	N	N	N	N	N	N	N	N	N	Y	Y	N	Y (detailed MTL)	Y
308	121	Alcantara 2020	Brazil	English	821	N	N	no label	randomized controlled trial	upper-middle	18-65	adults or most adults	0.52	mixed	ns	low	low	ns	Cereal and cereal products, Dairy, Fish and fish product, convenient foods, snack	multiple	N		package	Y	Y	N	Y	N	N	Y	N	N	N	N	N	N	N
309	122	Egnell 2020b	12 European countries	English	7434	N	N	no label	randomized controlled trial	high & upper-middle	>18	adults or most adults	0.5	mixed	ns	low	mixed	ns	Cereal and cereal products, bread & bakery product, convenient foods	multiple	N		package	Y	Y	N	Y	N	Y	Y	N	N	Y	Y	N	Y (detailed MTL)	Y
311	123	Sarda 2020	France	English	2006	Y	N	no label	Interrupted Time-Series Design	high		adults or most adults	0.52	mixed	ns	high	mixed	mixed		multiple	N		package	N	N	N	N	N	N	N	N	N	Y	N	N	N	Y

312	124	Taillie 2020b	Colombia	English	1997	N	N	no label	randomized controlled trial	upper-middle	>18	adults or most adults	0.5	mixed	mixed	high	ns	ns	Non-alcoholic beverages, Cereal and cereal products, bread & bakery product	multiple	N		package	Y	Y	Y	Y	N	N	Y	N	N	N	N	N	N	N
313	125	Aguenaou 2021	Morocco	English	814	N	N	no label	randomized controlled trial	lower-middle	>10	adults or most adults	0.6	mixed	ns	high	mixed	mixed	Meat & meat product, cereal and cereal products, diary	multiple	N		package	Y	Y	N	Y	N	N	Y	N	N	Y	Y	N	Y (detailed MTL)	Y
314	126	Andrews 2021a	US	English	711	Y	N	no label	randomized controlled trial	high		adults or most adults		ns	ns	ns	ns	ns	Meat & meat product	single	N		package	Y	Y	N	Y	N	N	Y	N	N	Y	Y	N	Y (detailed MTL)	N
314	126	Andrews 2021b	US	English	1,087	Y	N	no label	randomized controlled trial	high		adults or most adults		ns	ns	ns	ns	ns	Meat & meat product	single	N		package	Y	Y	N	Y	N	N	Y	N	N	Y	Y	N	Y (detailed MTL)	N
315	127	Ares 2021	Uruguay	English	1772	Y	N	no label	Interrupted Time-Series Design	high	>18	adults or most adults	0.67	mostly female		low	ns	ns	Non-alcoholic beverages, Cereal and cereal products, diary, convenient foods, snack foods, vegetabl	multiple	N		package	Y	Y	N	Y	N	N	Y	N	N	N	N	N	N	N
316	128	Asbridge 2021	UK	English	1185	N	N	no label	randomized controlled trial	high	>18	adults or most adults	0.55	mixed	white	high	ns	ns	Vegetable and fruit, cereal and cereal product, confectionary, snack food, bread	multiple	N		package	Y	N	Y	N	Y	Y	Y	N	Y	N	N	N	N	N
317	129	Bandeira 2021	Brazil	English	2,400	Y	N	no label	randomized controlled trial	upper-middle	>18	adults or most adults	0.51	mixed	ns	ns	mixed	ns	Sauce and spread, cereal and cereal product, bread and bakery product, snack food,	multiple	N		package	Y	Y	N	Y	N	N	Y	N	N	Y	Y	N	Y (detailed MTL)	N
318	130	De Temmerman 2021a	Belgium	English	292	N	N	no label	randomized controlled trial	high	30.82	adults or most adults		ns	ns	high	ns	mixed	Convenient food	single	N		package	N	N	N	N	N	N	N	N	N	Y	N	N	N	Y
318	130	De Temmerman 2021b	Belgium	English	415	N	N	no label	randomized controlled trial	high	37.66	adults or most adults	0.69	mostly female	ns	ns	ns	ns	Convenient food	single	N		package	N	N	N	N	N	N	N	N	N	Y	N	N	N	Y
319	131	Devia 2021a	Uruguay	English	377	N	N	no label	choice conjoint task	high	18-89	adults or most adults	0.64	mostly female	ns	low	ns	ns	Convenient food, sauce and spread, snack food	multiple	N		package	Y	Y	N	Y	N	N	Y	N	N	Y	N	N	N	N
319	131	Devia 2021b	Uruguay	English	413	N	N	no label	choice conjoint task	high	18-89	adults or most adults	0.66	mostly female	ns	low	ns	ns	Convenient food, sauce and spread, snack food	multiple	N		package	Y	Y	N	Y	N	N	Y	N	N	Y	N	N	N	N
320	132	Egnell 2021a	France	English	220	N	N	no label	randomized controlled trial	high	41.3	adults or most adults	0.87	mostly female	ns	high	low	ns		multiple	N		package	N	N	N	N	N	N	N	N	N	Y	N	N	N	Y
321	133	Egnell 2021b	France	English	2223	N	N	no label	randomized controlled trial	high	>18	adults or most adults	0.7	mostly female	ns	high	ns	ns		multiple	N		package	N	N	N	N	N	N	N	N	N	Y	N	N	N	Y
322	134	Hall 2021	France	English	1078	N	N	no label	randomized controlled trial	high	35.3	adults or most adults	0.58	mixed	white	high	mixed	ns	Non-alcoholic beverages	single	N		package	Y	Y	Y	Y	Y	Y	Y	N	Y	N	N	N	N	N
324	135	Mauri 2021	UK and Italy	English	180	N	N	no label	randomized controlled trial	high	>18	adults or most adults	0.717	mostly female	ns	ns	ns	ns	Non-alcoholic beverages, diery, convenient food	multiple	N		package	N	N	N	N	N	N	N	N	N	Y	Y	N	Y (detailed MTL)	N

325	136	Mediano Stoltze 2021	Chile	English	497	N	N	no label	randomized controlled trial	high	35.9	adults or most adults	0.5	mixed	ns	low	ns	ns	Cereal and cereal product	single	N		package	Y	Y	N	Y	N	N	Y	N	N	Y	N	N	N	N
326	137	Packer 2021	UK	English	3618	N	N	no label	randomized controlled trial	high	>18 years	adults or most adults	0.57	mixed	white	low	ns	ns	Convenie nt food, non-alcoholic drink, cereal and cereal product, snack food,	multiple	N		package	Y	Y	N	Y	N	N	Y	N	N	Y	Y	N	Y (detailed MTL)	Y
327	138	Vanderlee 2021	Canada	English	1498	Y	N	no label	randomized controlled trial	high	>18 years	adults or most adults	0.5	mixed	white	low	mixed	ns	nt food, cereal and cereal product, diary, vegetabl e and	multiple	N		package	Y	Y	N	Y	N	N	N	N	N	Y	Y	N	Y (simple MTL)	N

Supplementary References

1. Burton S, Biswas A. Preliminary Assessment of Changes in Labels Required by the Nutrition Labeling and Education Act of 1990. J Consum Aff. 1993;27(1):127-44. doi: 10.1111/j.1745-6606.1993.tb00741.x.
2. Bushman BJ. Effects of warning and information labels on consumption of full-fat, reduced-fat, and no-fat products. J Appl Psychol. 1998;83(1):97-101. Epub 1998/03/12. PubMed PMID: 9494441.
3. Jones G, Richardson M. An objective examination of consumer perception of nutrition information based on healthiness ratings and eye movements. Public Health Nutr. 2007;10(3):238-44. Epub 2007/02/10. doi: 10.1017/s1368980007258513. PubMed PMID: 17288620.
4. Borgmeier I, Westenhoefer J. Impact of different food label formats on healthiness evaluation and food choice of consumers: a randomized-controlled study. BMC Public Health. 2009;9:184. Epub 2009/06/16. doi: 10.1186/1471-2458-9-184. PubMed PMID: 19523212.
5. Drichoutis AC, Lazaridis P, Nayga Jr RM. Would consumers value food-away-from-home products with nutritional labels? Agribusiness. 2009;25(4):550-75. doi: 10.1002/agr.20224.
6. Gorton D, Ni Mhurchu C, Chen MH, Dixon R. Nutrition labels: a survey of use, understanding and preferences among ethnically diverse shoppers in New Zealand. Public Health Nutr. 2009;12(9):1359-65. Epub 2008/12/18. doi: 10.1017/s1368980008004059. PubMed PMID: 19087382.
7. Sacks G, Rayner M, Swinburn B. Impact of front-of-pack 'traffic-light' nutrition labelling on consumer food purchases in the UK. Health Promot Int. 2009;24(4):344-52. Epub 2009/10/10. doi: 10.1093/heapro/dap032. PubMed PMID: 19815614.
8. Fatimah S, Jr., Nik Ismail ND, Tee ES. Consumer understanding and preferences for different nutrition information panel formats. Malays J Nutr. 2010;16(2):243-50. Epub 2010/08/01. PubMed PMID: 22691929.
9. Sacks G, Tikellis K, Millar L, Swinburn B. Impact of 'traffic-light' nutrition information on online food purchases in Australia. Aust N Z J Public Health. 2011;35(2):122-6. Epub 2011/04/06. doi: 10.1111/j.1753-6405.2011.00684.x. PubMed PMID: 21463406.
10. Temple JL, Johnson KM, Archer K, Lacarte A, Yi C, Epstein LH. Influence of simplified nutrition labeling and taxation on laboratory energy intake in adults. Appetite. 2011;57(1):184-92. Epub 2011/05/17. doi: 10.1016/j.appet.2011.04.018. PubMed PMID: 21569807.
11. van Herpen E, Trijp HCMV. Front-of-pack nutrition labels. Their effect on attention and choices when consumers have varying goals and time constraints. Appetite. 2011;57(1):148-60. doi: 10.1016/j.appet.2011.04.011.
12. Ares G, Gimenez A, Bruzzone F, Antunez L, Sapolinski A, Vidal L, et al. Attentional capture and understanding of nutrition labelling: a study based on response times. Int J Food Sci Nutr. 2012;63(6):679-88. Epub 2012/01/26. doi: 10.3109/09637486.2011.652598. PubMed PMID: 22273500.
13. Koenigstorfer J, Groeppel-Klein A, Kamm F, Rohr M, Wentura D. The traffic light colors red and green in the context of healthy food decision-making. Adv Consum Res. 2012;40:945-6.
14. McLean R, Hoek J, Hedderley D. Effects of alternative label formats on choice of high- and low-sodium products in a New Zealand population sample. Public Health Nutr. 2012;15(5):783-91. Epub 2012/01/28. doi: 10.1017/s1368980011003508. PubMed PMID: 22281127.
15. Roberto CA, Bragg MA, Schwartz MB, Seamans MJ, Musicus A, Novak N, et al. Facts up front versus traffic light food labels: a randomized controlled trial. Am J Prev Med. 2012;43(2):134-41. Epub 2012/07/21. doi: 10.1016/j.amepre.2012.04.022. PubMed PMID: 22813677.
16. Antúnez L, Vidal L, Sapolinski A, Giménez A, Maiche A, Ares G. How do design features influence consumer attention when looking for nutritional information on food labels? Results from an eye-tracking study on pan bread labels. Int J Food Sci Nutr. 2013;64(5):515-27. doi: 10.3109/09637486.2012.759187.
17. Goodman S, Hammond D, Hanning R, Sheeshka J. The impact of adding front-of-package sodium content labels to grocery products: an experimental study. Public Health Nutr. 2013;16(3):383-91. Epub 2012/08/04. doi: 10.1017/s1368980012003485. PubMed PMID: 22857386.
18. Mejean C, Macouillard P, Péneau S, Hercberg S, Castetbon K. Consumer acceptability and understanding of front-of-pack nutrition labels. J Hum Nutr Diet. 2013;26(5):494-503. doi: 10.1111/jhn.12039.
19. Savoie N, Barlow K, Harvey KL, Binnie MA, Pasut L. Consumer perceptions of front-of-package labelling systems and healthiness of foods. Can J Public Health. 2013;104(5):e359-e63.
20. Sonnenberg L, Gelsomin E, Levy DE, Riis J, Barraclough S, Thorndike AN. A traffic light food labeling intervention increases consumer awareness of health and healthy choices at the point-of-purchase. Prev Med. 2013;57(4):253-7. Epub 2013/07/19. doi: 10.1016/j.ypmed.2013.07.001. PubMed PMID: 23859926.
21. Ares G, Mawad F, Giménez A, Maiche A. Influence of rational and intuitive thinking styles on food choice: Preliminary evidence from an eye-tracking study with yogurt labels. Food Qual Prefer. 2014;31(1):28-37. doi: 10.1016/j.foodqual.2013.07.005.
22. Emrich TE, Qi Y, Mendoza JE, Lou W, Cohen JE, L'Abbé MR. Consumer perceptions of the Nutrition Facts table and front-of-pack nutrition rating systems. Physiologie appliquee, nutrition et metabolisme Appl Physiol Nutr Metab. 2014;39(4):417-24. doi: 10.1139/apnm-2013-0304. PubMed PMID: CN-01157755.
23. Koenigstorfer J, Wąsowicz-Kiryło G, Styśko-Kunkowska M, Groeppel-Klein A. Behavioural effects of directive cues on front-of-package nutrition information: The combination matters! Public Health Nutr. 2014;17(9):2115-21. doi: 10.1017/S136898001300219X.
24. Maubach N, Hoek J, Mather D. Interpretive front-of-pack nutrition labels. Comparing competing recommendations. Appetite. 2014;82:67-77. Epub 2014/07/20. doi: 10.1016/j.appet.2014.07.006. PubMed PMID: 25038407.
25. Van Herpen E, Hieke S, Van Trijp HCM. Inferring product healthfulness from nutrition labelling. The influence of reference points. Appetite. 2014;72:138-49. doi: 10.1016/j.appet.2013.10.012.
26. Watson WL, Kelly B, Hector D, Hughes C, King L, Crawford J, et al. Can front-of-pack labelling schemes guide healthier food choices? Australian shoppers' responses to seven labelling formats. Appetite. 2014;72:90-7. Epub 2013/10/16. doi: 10.1016/j.appet.2013.09.027. PubMed PMID: 24126243.
27. Ducrot P, Méjean C, Julia C, Kesse-Guyot E, Touvier M, Fezeu LK, et al. Objective understanding of front-of-package nutrition labels among nutritionally at-risk individuals. Nutrients. 2015;7(8):7106-25. doi: 10.3390/nu7085325.
28. Hamlin RP, McNeill LS, Moore V. The impact of front-of-pack nutrition labels on consumer product evaluation and choice: an experimental study. Public Health Nutr. 2015;18(12):2126-34. Epub 2014/12/23. doi: 10.1017/s1368980014002997. PubMed PMID: 25529170.
29. Mawad F, Trías M, Giménez A, Maiche A, Ares G. Influence of cognitive style on information processing and selection of yogurt labels: Insights from an eye-tracking study. Food Res Int. 2015;74:1-9. doi: 10.1016/j.foodres.2015.04.023.
30. Pauline D, Caroline M, Chantal J, Emmanuelle KG, Mathilde T, Léopold F, et al. Effectiveness of front-of-pack nutrition labels in french adults: Results from the nutrinet-santé cohort study. PLoS One. 2015;10(10). doi: 10.1371/journal.pone.0140898.
31. Siegrist M, Leins-Hess R, Keller C. Which front-of-pack nutrition label is the most efficient one? The results of an eye-tracker study. Food Qual Prefer. 2015;39:183-90. doi: 10.1016/j.foodqual.2014.07.010.
32. Acton RB, Vanderlee L, White C, Hammond D. The efficacy of calorie labelling formats on pre-packaged foods: An experimental study among adolescents and young adults in Canada. Can J Public Health. 2016;107(3):e296-e302. Epub 2016/10/21. doi: 10.17269/cjph.107.5513. PubMed PMID: 27763846.
33. Bollard T, Maubach N, Walker N, Ni Mhurchu C. Effects of plain packaging, warning labels, and taxes on young people's predicted sugar-sweetened beverage preferences: an experimental study. Int J Behav Nutr Phys Act. 2016;13(1):95. doi:

10.1186/s12966-016-0421-7. PubMed PMID: 27580589.
34. Crosetto P, Muller L, Ruffieux B. Helping consumers with a front-of-pack label: Numbers or colors?: Experimental comparison between Guideline Daily Amount and Traffic Light in a diet-building exercise. J Econ Psychol. 2016;55:30-50. doi: 10.1016/j.joep.2016.03.006.
35. Ducrot P, Julia C, Méjean C, Kesse-Guyot E, Touvier M, Fezeu LK, et al. Impact of different front-of-pack nutrition labels on consumer purchasing intentions: A randomized controlled trial. Am J Prev Med. 2016;50(5):627-36. doi: 10.1016/j.amepre.2015.10.020.
36. Talati T, Pettigrew S, Dixon H, Neal B, Ball K, Hughes C. Do health claims and front-of-pack labels lead to a positivity bias in unhealthy foods? Nutrients. 2016;8(12). doi: 10.3390/nu8120787.
37. VanEpps EM, Roberto CA. The Influence of Sugar-Sweetened Beverage Warnings: A Randomized Trial of Adolescents' Choices and Beliefs. Am J Prev Med. 2016;51(5):664-72. Epub 2016/09/13. doi: 10.1016/j.amepre.2016.07.010. PubMed PMID: 27617366.
38. Arrua A, Curutchet MR, Rey N, Barreto P, Golovchenko N, Sellanes A, et al. Impact of front-of-pack nutrition information and label design on children's choice of two snack foods: Comparison of warnings and the traffic-light system. Appetite. 2017;116:139-46. Epub 2017/04/22. doi: 10.1016/j.appet.2017.04.012. PubMed PMID: 28428151.
39. Arrúa A, Machín L, Curutchet MR, Martínez J, Antúnez L, Alcaire F, et al. Warnings as a directive front-of-pack nutrition labelling scheme: comparison with the Guideline Daily Amount and traffic-light systems. Public Health Nutr. 2017;20(13):2308-17. doi: 10.1017/S1368980017000866.
40. Boncinelli F, Gerini F, Pagnotta G, Alfnes F. Warning labels on junk food: experimental evidence. Int J Consum Stud. 2017;41(1):46-53. doi: 10.1111/ijcs.12312.
41. Julia C, Péneau S, Buscail C, Gonzalez R, Touvier M, Hercberg S, et al. Perception of different formats of front-of-pack nutrition labels according to sociodemographic, lifestyle and dietary factors in a French population: Cross-sectional study among the NutriNet-Santé cohort participants. BMJ Open. 2017;7(6). doi: 10.1136/bmjopen-2017-016108.
42. Neal B, Crino M, Dunford E, Gao A, Greenland R, Li N, et al. Effects of different types of front-of-pack labelling information on the healthiness of food purchases—a randomised controlled trial. Nutrients. 2017;9(12). doi: 10.3390/nu9121284. PubMed PMID: CN-01621767.
43. Ni Mhurchu C, Volkova E, Jiang Y, Eyles H, Michie J, Neal B, et al. Effects of interpretive nutrition labels on consumer food purchases: the Starlight randomized controlled trial. Am J Clin Nutr. 2017;105(3):695-704. Epub 2017/02/06. doi: 10.3945/ajcn.116.144956. PubMed PMID: 28148503.
44. Talati Z, Pettigrew S, Ball K, Hughes C, Kelly B, Neal B, et al. The relative ability of different front-of-pack labels to assist consumers discriminate between healthy, moderately healthy, and unhealthy foods. Food Qual Prefer. 2017;59:109-13. doi: 10.1016/j.foodqual.2017.02.010.
45. Yoo HJ, Machín L, Arrúa A, Antúnez L, Vidal L, Giménez A, et al. Children and adolescents' attitudes towards sugar reduction in dairy products. Food Res Int (Ottawa, Ont). 2017;94:108-14. doi: 10.1016/j.foodres.2017.02.005.
46. Acton RB, Hammond D. The impact of price and nutrition labelling on sugary drink purchases: Results from an experimental marketplace study. Appetite. 2018;121:129-37. Epub 2017/11/18. doi: 10.1016/j.appet.2017.11.089. PubMed PMID: 29146460.
47. Acton RB, Vanderlee L, Hammond D. Influence of front-of-package nutrition labels on beverage healthiness perceptions: Results from a randomized experiment. Prev Med. 2018;115:83-9. Epub 2018/08/27. doi: 10.1016/j.ypmed.2018.08.022. PubMed PMID: 30145345.
48. Ares G, Aschemann-Witzel J, Curutchet MR, Antunez L, Machin L, Vidal L, et al. Product reformulation in the context of nutritional warning labels: Exploration of consumer preferences towards food concepts in three food categories. Food Res Int (Ottawa, Ont). 2018;107:669-74. Epub 2018/03/28. doi: 10.1016/j.foodres.2018.03.021. PubMed PMID: 29580533.
49. Ares G, Aschemann-Witzel J, Curutchet MR, Antúnez L, Machín L, Vidal L, et al. Nutritional warnings and product substitution or abandonment: Policy implications derived from a repeated purchase simulation. Food Qual Prefer. 2018;65:40-8. doi: 10.1016/j.foodqual.2017.12.001.
50. Ares G, Varela F, Machin L, Antúnez L, Giménez A, Curutchet MR, et al. Comparative performance of three interpretative front-of-pack nutrition labelling schemes: Insights for policy making. Food Qual Prefer. 2018;68:215-25. doi: 10.1016/j.foodqual.2018.03.007.
51. Billich N, Blake MR, Backholer K, Cobcroft M, Li V, Peeters A. The effect of sugar-sweetened beverage front-of-pack labels on drink selection, health knowledge and awareness: An online randomised controlled trial. Appetite. 2018;128:233-41. Epub 2018/06/08. doi: 10.1016/j.appet.2018.05.149. PubMed PMID: 29879450.
52. David IA, Krutman L, Fernandez-Santaella MC, Andrade JR, Andrade EB, Oliveira L, et al. Appetitive drives for ultra-processed food products and the ability of text warnings to counteract consumption predispositions. Public Health Nutr. 2018;21(3):543-57. Epub 2017/11/28. doi: 10.1017/s1368980017003263. PubMed PMID: 29173214.
53. Donnelly GE, Zatz LY, Svirsky D, John LK. The Effect of Graphic Warnings on Sugary-Drink Purchasing. Psychol Sci. 2018;29(8):1321-33. Epub 2018/06/19. doi: 10.1177/0956797618766361. PubMed PMID: 29912624.
54. Egnell M, Ducrot P, Touvier M, Allès B, Hercberg S, Kesse-Guyot E, et al. Objective understanding of Nutri-Score Front-Of-Package nutrition label according to individual characteristics of subjects: Comparisons with other format labels. PLoS One. 2018;13(8). doi: 10.1371/journal.pone.0202095.
55. Egnell M, Kesse-Guyot E, Galan P, Touvier M, Rayner M, Jewell J, et al. Impact of front-of-pack nutrition labels on portion size selection: An experimental study in a French cohort. Nutrients. 2018;10(9). doi: 10.3390/nu10091268.
56. Goodman S, Vanderlee L, Acton R, Mahamad S, Hammond D. The Impact of Front-of-Package Label Design on Consumer Understanding of Nutrient Amounts. Nutrients. 2018;10(11). Epub 2018/11/08. doi: 10.3390/nu10111624. PubMed PMID: 30400146.
57. Gorski Findling MT, Werth PM, Musicus AA, Bragg MA, Graham DJ, Elbel B, et al. Comparing five front-of-pack nutrition labels' influence on consumers' perceptions and purchase intentions. Prev Med. 2018;106:114-21. Epub 2017/10/27. doi: 10.1016/j.ypmed.2017.10.022. PubMed PMID: 29066375.
58. Khandpur N, de Morais Sato P, Mais LA, Bortoletto Martins AP, Spinillo CG, Garcia MT, et al. Are Front-of-Package Warning Labels More Effective at Communicating Nutrition Information than Traffic-Light Labels? A Randomized Controlled Experiment in a Brazilian Sample. Nutrients. 2018;10(6). Epub 2018/05/31. doi: 10.3390/nu10060688. PubMed PMID: 29843449.
59. Lima M, Ares G, Deliza R. How do front of pack nutrition labels affect healthfulness perception of foods targeted at children? Insights from Brazilian children and parents. Food Qual Prefer. 2018;64:111-9. doi: 10.1016/j.foodqual.2017.10.003.
60. Machin L, Arrua A, Gimenez A, Curutchet MR, Martinez J, Ares G. Can nutritional information modify purchase of ultra-processed products? Results from a simulated online shopping experiment. Public Health Nutr. 2018;21(1):49-57. Epub 2017/07/19. doi: 10.1017/s1368980017001185. PubMed PMID: 28716163.
61. Machin L, Aschemann-Witzel J, Curutchet MR, Gimenez A, Ares G. Traffic Light System Can Increase Healthfulness Perception: Implications for Policy Making. J Nutr Educ Behav. 2018;50(7):668-74. Epub 2018/04/09. doi: 10.1016/j.jneb.2018.03.005.

PubMed PMID: 29627330.
62. Machín L, Aschemann-Witzel J, Curutchet MR, Giménez A, Ares G. Does front-of-pack nutrition information improve consumer ability to make healthful choices? Performance of warnings and the traffic light system in a simulated shopping experiment. Appetite. 2018;121:55-62. doi: 10.1016/j.appet.2017.10.037.
63. Mantzari E, Vasiljevic M, Turney I, Pilling M, Marteau T. Impact of warning labels on sugar-sweetened beverages on parental selection: An online experimental study. Prev Med Rep. 2018;12:259-67. doi: 10.1016/j.pmedr.2018.10.016.
64. Mazza MC, Dynan L, Siegel RM, Tucker AL. Nudging Healthier Choices in a Hospital Cafeteria: Results From a Field Study. Health Promot Pract. 2018;19(6):925-34. doi: 10.1177/1524839917740119.
65. Moran AJ, Roberto CA. Health Warning Labels Correct Parents' Misperceptions About Sugary Drink Options. Am J Prev Med. 2018;55(2):e19-e27. Epub 2018/06/16. doi: 10.1016/j.amepre.2018.04.018. PubMed PMID: 29903567.
66. Talati Z, Norman R, Kelly B, Dixon H, Neal B, Miller C, et al. A randomized trial assessing the effects of health claims on choice of foods in the presence of front-of-pack labels. Am J Clin Nutr. 2018;108(6):1275-82. doi: 10.1093/ajcn/nqy248.
67. Talati Z, Pettigrew S, Kelly B, Ball K, Neal B, Dixon H, et al. Can front-of-pack labels influence portion size judgements for unhealthy foods? Public Health Nutr. 2018;21(15):2776-81. Epub 2018/07/19. doi: 10.1017/s1368980018001702. PubMed PMID: 30017011.
68. Acton RB, Jones AC, Kirkpatrick SI, Roberto CA, Hammond D. Taxes and front-of-package labels improve the healthiness of beverage and snack purchases: a randomized experimental marketplace. Int J Behav Nutr Phys Act. 2019;16(1):46. Epub 2019/05/23. doi: 10.1186/s12966-019-0799-0. PubMed PMID: 31113448.
69. Alonso-Dos-Santos M, Ulloa RQ, Quintana ÁS, Quijada DV, Nazel PF. Nutrition labeling schemes and the time and effort of consumer processing. Sustainability (Switzerland). 2019;11(4). doi: 10.3390/su11041079.
70. Ang FJL, Agrawal S, Finkelstein EA. Pilot randomized controlled trial testing the influence of front-of-pack sugar warning labels on food demand. BMC Public Health. 2019;19(1):164. Epub 2019/02/09. doi: 10.1186/s12889-019-6496-8. PubMed PMID: 30732609.
71. Egnell M, Crosetto P, D'Almeida T, Kesse-Guyot E, Touvier M, Ruffieux B, et al. Modelling the impact of different front-of-package nutrition labels on mortality from non-communicable chronic disease. Int J Behav Nutr Phys Act. 2019;16(1). doi: 10.1186/s12966-019-0817-2.
72. Galan P, Egnell M, Salas-Salvadó J, Babio N, Pettigrew S, Hercberg S, et al. Understanding of different front-of-package labels by the Spanish population: Results of a comparative study. Endocrinol Diabetes Nutr. 2019. doi: 10.1016/j.endinu.2019.03.013.
73. Grummon AH, Hall MG, Taillie LS, Brewer NT. How should sugar-sweetened beverage health warnings be designed? A randomized experiment. Prev Med. 2019;121:158-66. Epub 2019/02/18. doi: 10.1016/j.ypmed.2019.02.010. PubMed PMID: 30772370.
74. Hayward LE, Vartanian LR. Potential unintended consequences of graphic warning labels on sugary drinks: do they promote obesity stigma? Obes Sci Pract. 2019. doi: 10.1002/osp4.353.
75. Karamanos V, Hobbs JE, Slade P. Consumer responses to private nutrition signals. J Food Prod Mark. 2019;25(2):111-36. doi: 10.1080/10454446.2018.1498044.
76. Lima M, de Alcantara M, Ares G, Deliza R. It is not all about information! Sensory experience overrides the impact of nutrition information on consumers' choice of sugar-reduced drinks. Food Qual Prefer. 2019;74:1-9. doi: 10.1016/j.foodqual.2018.12.013.
77. Machín L, Curutchet MR, Giménez A, Aschemann-Witzel J, Ares G. Do nutritional warnings do their work? Results from a choice experiment involving snack products. Food Qual Prefer. 2019;77:159-65. doi: 10.1016/j.foodqual.2019.05.012.
78. Marette S, Nabec L, Durieux F. Improving Nutritional Quality of Consumers' Food Purchases With Traffic-Lights Labels: An Experimental Analysis. J Consum Policy (Dordr). 2019. doi: 10.1007/s10603-019-09420-5.
79. Popova L, Nonnemaker J, Taylor N, Bradfield B, Kim A. Warning Labels on Sugar-sweetened Beverages: An Eye Tracking Approach. Am J Health Behav. 2019;43(2):406-19. Epub 2019/02/28. doi: 10.5993/ajhb.43.2.16. PubMed PMID: 30808479.
80. Rosenblatt DH, Dixon H, Wakefield M, Bode S. Evaluating the influence of message framing and graphic imagery on perceptions of food product health warnings. Food Qual Prefer. 2019;77:32-42. doi: 10.1016/j.foodqual.2019.05.003.
81. Schnettler B, Ares G, Sepúlveda N, Bravo S, Villalobos B, Hueche C, et al. How do consumers perceive reformulated foods after the implementation of nutritional warnings? Case study with frankfurters in Chile. Food Qual Prefer. 2019;74:179-88. doi: 10.1016/j.foodqual.2019.01.021.
82. Schnettler B, Ares G, Sepúlveda N, Bravo S, Villalobos B, Hueche C, et al. Are consumers willing to pay more for reformulated processed meat products in the context of the implementation of nutritional warnings? Case study with frankfurters in Chile. Meat Sci. 2019;152:104-8. doi: 10.1016/j.meatsci.2019.02.007.
83. Velasco Vizcaíno F, Velasco A. The battle between brands and nutritional labels: How brand familiarity decreases consumers' alertness toward traffic light nutritional labels. J Bus Res. 2019;101:637-50. doi: 10.1016/j.jbusres.2019.02.054.
84. Nobrega L, Ares G, Deliza R. Are nutritional warnings more efficient than claims in shaping consumers' healthfulness perception? Food Qual Prefer. 2020;79. doi: 10.1016/j.foodqual.2019.103749.
85. Bix L, Sundar RP, Bello NM, Peltier C, Weatherspoon LJ, Becker MW. To See or Not to See: Do Front of Pack Nutrition Labels Affect Attention to Overall Nutrition Information? PLoS One. 2015;10(10):e0139732. Epub 2015/10/22. doi: 10.1371/journal.pone.0139732. PubMed PMID: 26488611.
86. Hobin E, Sacco J, Vanderlee L, White CM, Zuo F, Sheeshka J, et al. A randomized trial testing the efficacy of modifications to the nutrition facts table on comprehension and use of nutrition information by adolescents and young adults in Canada. Health Promot Chronic Dis Prev Can. 2015;35(10):173-83. Epub 2015/12/18. PubMed PMID: 26674187.
87. Egnell M, Boutron I, Peneau S, Ducrot P, Touvier M, Galan P, et al. Front-of-Pack Labeling and the Nutritional Quality of Students' Food Purchases: A 3-Arm Randomized Controlled Trial. Am J Public Health. 2019;109(8):1122-9. Epub 2019/06/21. doi: 10.2105/ajph.2019.305115. PubMed PMID: 31219721.
88. Poquet D, Ginon E, Goubel B, Chabanet C, Marette S, Issanchou S, et al. Impact of a front-of-pack nutritional traffic-light label on the nutritional quality and the hedonic value of mid-afternoon snacks chosen by mother-child dyads. Appetite. 2019;143:104425. Epub 2019/08/30. doi: 10.1016/j.appet.2019.104425. PubMed PMID: 31465810.
89. Trudel R, Murray KB, Kim S, Chen S. The impact of traffic light color-coding on food health perceptions and choice. J Exp Psychol Appl. 2015;21(3):255-75. Epub 2015/06/30. doi: 10.1037/xap0000049. PubMed PMID: 26121372.
90. Crockett RA, Jebb SA, Hankins M, Marteau TM. The impact of nutritional labels and socioeconomic status on energy intake. An experimental field study. Appetite. 2014;81:12-9. Epub 2014/06/01. doi: 10.1016/j.appet.2014.05.024. PubMed PMID: 24879885.
91. Egnell M, Talati Z, Pettigrew S, Galan P, Hercberg S, Julia C. Comparison of front-of-pack labels to help German consumers understand the nutritional quality of food products: Color-coded labels outperform all other systems. Ernahrungs Umschau. 2019;66(5):76-84. doi: 10.4455/eu.2019.020.
92. Finkelstein EA, Ang FJL, Doble B, Wong WHM, Van Dam RM. A randomized controlled trial evaluating the relative effectiveness of the multiple trafic light and nutri-score front of package nutrition labels. Nutrients. 2019;11(9). doi: 10.3390/nu11092236.
93. Grummon AH, Taillie LS, Golden SD, Hall MG, Ranney LM, Brewer NT. Sugar-Sweetened Beverage Health Warnings and Purchases: A Randomized Controlled Trial. Am J Prev Med. 2019;57(5):601-10. Epub 2019/10/07. doi: 10.1016/j.amepre.2019.06.019. PubMed PMID: 31586510.

94. Nieto C, Jauregui A, Contreras-Manzano A, Arillo-Santillan E, Barquera S, White CM, et al. Understanding and use of food labeling systems among Whites and Latinos in the United States and among Mexicans: Results from the International Food Policy Study, 2017. Int J Behav Nutr Phys Act. 2019;16(1):87. Epub 2019/10/19. doi: 10.1186/s12966-019-0842-1. PubMed PMID: 31623663.
95. Talati Z, Egnell M, Hercberg S, Julia C, Pettigrew S. Food Choice Under Five Front-of-Package Nutrition Label Conditions: An Experimental Study Across 12 Countries. Am J Public Health. 2019:e1-e6. Epub 2019/10/18. doi: 10.2105/ajph.2019.305319. PubMed PMID: 31622139.
96. Talati Z, Egnell M, Hercberg S, Julia C, Pettigrew S. Consumers' Perceptions of Five Front-of-Package Nutrition Labels: An Experimental Study Across 12 Countries. Nutrients. 2019;11(8). Epub 2019/08/21. doi: 10.3390/nu11081934. PubMed PMID: 31426450.
97. Mantzari E, Pechey R, Codling S, Sexton O, Hollands GJ, Marteau TM. The impact of 'on-pack' pictorial health warning labels and calorie information labels on drink choice: A laboratory experiment. Appetite. 2020;145. doi: 10.1016/j.appet.2019.104484.
98. Defago D, Geng JF, Molina O, Santa María D. Can traffic light nutritional labels induce healthier consumer choices? Experimental evidence from a developing country. Int J Consum Stud. 2019.
99. Egnell M, Galan P, Farpour-Lambert NJ, Talati Z, Pettigrew S, Hercberg S, et al. Compared to other front-of-pack nutrition labels, the Nutri-Score emerged as the most efficient to inform Swiss consumers on the nutritional quality of food products. PLoS One. 2020;15(2):e0228179. Epub 2020/02/29. doi: 10.1371/journal.pone.0228179. PubMed PMID: 32107489.
100. Franco-Arellano B, Vanderlee L, Ahmed M, Oh A, L'Abbé M. Influence of front-of-pack labelling and regulated nutrition claims on consumers' perceptions of product healthfulness and purchase intentions: A randomized controlled trial. Appetite. 2020;149:104629. Epub 2020/02/18. doi: 10.1016/j.appet.2020.104629. PubMed PMID: 32061707.
101. Gabor AM, Stojnić B, Ostić DB. Effects of different nutrition labels on visual attention and accuracy of nutritional quality perception–Results of an experimental eye-tracking study. Food Qual Prefer. 2020;84:103948. doi: 10.1016/j.foodqual.2020.103948.
102. Hagmann D, Siegrist M. Nutri-Score, multiple traffic light and incomplete nutrition labelling on food packages: Effects on consumers' accuracy in identifying healthier snack options. Food Qual Prefer. 2020;83:103894. doi: 10.1016/j.foodqual.2020.103894.
103. Hall MG, Grummon AH, Lazard AJ, Maynard OM, Taillie LS. Reactions to graphic and text health warnings for cigarettes, sugar-sweetened beverages, and alcohol: An online randomized experiment of US adults. Prev Med. 2020:106120. Epub 2020/05/22. doi: 10.1016/j.ypmed.2020.106120. PubMed PMID: 32437703.
104. Hall MG, Lazard AJ, Grummon AH, Mendel JR, Taillie LS. The impact of front-of-package claims, fruit images, and health warnings on consumers' perceptions of sugar-sweetened fruit drinks: Three randomized experiments. Prev Med. 2020;132:105998. Epub 2020/01/27. doi: 10.1016/j.ypmed.2020.105998. PubMed PMID: 31982477.
105. Hernández-Nava LG, Egnell M, Aguilar-Salinas CA, Córdova-Villalobos J, Barriguete-Meléndez JA, Pettigrew S, et al. [Impact of different front-of-pack nutrition labels on foods according to their nutritional quality: a comparative study in Mexico]. Salud Publica Mex. 2019;61(5):609-18. Epub 2019/10/30. doi: 10.21149/10318. PubMed PMID: 31661738.
106. Jáuregui A, Vargas-Meza J, Nieto C, Contreras-Manzano A, Alejandro NZ, Tolentino-Mayo L, et al. Impact of front-of-pack nutrition labels on consumer purchasing intentions: a randomized experiment in low- and middle-income Mexican adults. BMC Public Health. 2020;20(1):463. Epub 2020/04/08. doi: 10.1186/s12889-020-08549-0. PubMed PMID: 32252716.
107. Retno D. The Impact of Front-of-Package Traffic Light (Foptl) in the Senior High School Students' Nutrition Labels Comprehension. Curr Res Nutr Food Sci. 2019;7(3):918. doi: 10.12944/CRNFSJ.7.3.30
108. Taillie LS, Reyes M, Colchero MA, Popkin B, Corvalán C. An evaluation of Chile's Law of Food Labeling and Advertising on sugar-sweetened beverage purchases from 2015 to 2017: A before-and-after study. PLoS Med. 2020;17(2):e1003015. Epub 2020/02/12. doi: 10.1371/journal.pmed.1003015. PubMed PMID: 32045424.
109. Uribe R, Manzur E, Cornejo C. Varying the Number of FOP Warnings on Hedonic and Utilitarian Food Products: Evidence from Chile. J Food Prod Mark. 2020;26(2):123-43. doi: 10.1080/10454446.2020.1738971.
110. Vandevijvere S, Vermote M, Egnell M, Galan P, Talati Z, Pettigrew S, et al. Consumers' food choices, understanding and perceptions in response to different front-of-pack nutrition labelling systems in Belgium: results from an online experimental study. Arch Public Health. 2020;78:30. Epub 2020/04/09. doi: 10.1186/s13690-020-00404-3. PubMed PMID: 32266069.
111. Vargas-Meza J, Jáuregui A, Contreras-Manzano A, Nieto C, Barquera S. Acceptability and understanding of front-of-pack nutritional labels: an experimental study in Mexican consumers. BMC Public Health. 2019;19(1):1751. Epub 2020/01/01. doi: 10.1186/s12889-019-8108-z. PubMed PMID: 31888575.
112. Julia C, Blanchet O, Mejean C, Peneau S, Ducrot P, Alles B, et al. Impact of the front-of-pack 5-colour nutrition label (5-CNL) on the nutritional quality of purchases: an experimental study. Int J Behav Nutr Phys Act. 2016;13(1):101. Epub 2016/09/21. doi: 10.1186/s12966-016-0416-4. PubMed PMID: 27645372.
113. Roberto CA, Wong D, Musicus A, Hammond D. The Influence of Sugar-Sweetened Beverage Health Warning Labels on Parents' Choices. Pediatrics. 2016;137(2):e20153185. Epub 2016/01/16. doi: 10.1542/peds.2015-3185. PubMed PMID: 26768346.
114. Andreeva VA, Egnell M, Handjieva-Darlenska T, Talati Z, Touvier M, Galan P, et al. Bulgarian consumers' objective understanding of front-of-package nutrition labels: a comparative, randomized study. Arch Public Health. 2020;78:35. Epub 2020/06/13. doi: 10.1186/s13690-020-00416-z. PubMed PMID: 32528678.
115. Blitstein JL, Guthrie JF, Rains C. Low-Income Parents' Use of Front-of-Package Nutrition Labels in a Virtual Supermarket. J Nutr Educ Behav. 2020. Epub 2020/06/02. doi: 10.1016/j.jneb.2020.04.003. PubMed PMID: 32475704.
116. Clarke N, Pechey E, Mantzari E, Blackwell AKM, De-Loyde K, Morris RW, et al. Impact of health warning labels on snack selection: An online experimental study. Appetite. 2020;154:104744. Epub 2020/06/21. doi: 10.1016/j.appet.2020.104744. PubMed PMID: 32562806.
117. Fialon M, Egnell M, Talati Z, Galan P, Dréano-Trécant L, Touvier M, et al. Effectiveness of Different Front-of-Pack Nutrition Labels among Italian Consumers: Results from an Online Randomized Controlled Trial. Nutrients. 2020;12(8). Epub 2020/08/06. doi: 10.3390/nu12082307. PubMed PMID: 32752021.
118. Hamlin R, Hamlin B. An Experimental Comparison of the Impact of 'Warning' and 'Health Star Rating' FoP Labels on Adolescents' Choice of Breakfast Cereals in New Zealand. Nutrients. 2020;12(6). Epub 2020/05/30. doi: 10.3390/nu12061545. PubMed PMID: 32466408.
119. Panzone LA, Sniehotta FF, Comber R, Lemke F. The effect of traffic-light labels and time pressure on estimating kilocalories and carbon footprint of food. Appetite. 2020:104794. Epub 2020/08/12. doi: 10.1016/j.appet.2020.104794. PubMed PMID: 32781081.
120. Santos O, Alarcão V, Feteira-Santos R, Fernandes J, Virgolino A, Sena C, et al. Impact of different front-of-pack nutrition labels on online food choices. Appetite. 2020;154:104795. Epub 2020/08/17. doi: 10.1016/j.appet.2020.104795. PubMed PMID: 32798050.
121. Alcantara MD, Ares G, de Castro IPL, Deliza R. Gain vs. loss-framing for reducing sugar consumption: Insights from a choice experiment with six product categories. Food Res Int. 2020;136. doi: 10.1016/j.foodres.2020.109458. PubMed PMID: 32846549.
122. Egnell M, Talati Z, Galan P, Andreeva VA, Vandevijvere S, Gombaud M, et al. Objective understanding of the Nutri-score front-of-pack label by European consumers and its effect on food choices: an online experimental study. Int J Behav Nutr Phys

Act. 2020;17(1). doi: 10.1186/s12966-020-01053-z. PubMed PMID: 33213459.
123. Sarda B, Julia C, Serry AJ, Ducrot P. Appropriation of the front-of-pack nutrition label nutri-score across the french population: Evolution of awareness, support, and purchasing behaviors between 2018 and 2019. Nutrients. 2020;12(9):1-12. doi: 10.3390/nu12092887. PubMed PMID: 32971744.
124. Taillie LS, Hall MG, Gómez LF, Higgins I, Bercholz M, Murukutla N, et al. Designing an effective front-of-package warning label for food and drinks high in added sugar, sodium, or saturated fat in colombia: An online experiment. Nutrients. 2020;12(10):1-20. doi: 10.3390/nu12103124. PubMed PMID: 33066130.
125. Aguenaou H, El Ammari L, Bigdeli M, El Hajjab A, Lahmam H, Labzizi S, et al. Comparison of appropriateness of Nutri-Score and other front-of-pack nutrition labels across a group of Moroccan consumers: awareness, understanding and food choices. Arch Public Health. 2021;79(1):71. Epub 2021/05/08. doi: 10.1186/s13690-021-00595-3. PubMed PMID: 33957970.
126. Andrews JC, Netemeyer R, Burton S, Kees J. What consumers actually know: The role of objective nutrition knowledge in processing stop sign and traffic light front-of-pack nutrition labels. J Bus Res. 2021;128:140-55. doi: 10.1016/j.jbusres.2021.01.036.
127. Ares G, Antúnez L, Curutchet MR, Galicia L, Moratorio X, Giménez A, et al. Immediate effects of the implementation of nutritional warnings in Uruguay: Awareness, self-reported use and increased understanding. Public Health Nutr. 2021;24(2):364-75. doi: 10.1017/S1368980020002517. PubMed PMID: 32782052
128. Asbridge SCM, Pechey E, Marteau TM, Hollands GJ. Effects of pairing health warning labels with energy-dense snack foods on food choice and attitudes: Online experimental study. Appetite. 2021;160. doi: 10.1016/j.appet.2020.105090. PubMed PMID: 33373631.
129. Bandeira LM, Pedroso J, Toral N, Gubert MB. Performance and perception on front-of-package nutritional labeling models in Brazil. Rev Saude Publica. 2021;55:19. doi: 10.11606/s1518-8787.2021055002395. PubMed PMID: 33978115.
130. De Temmerman J, Heeremans E, Slabbinck H, Vermeir I. The impact of the Nutri-Score nutrition label on perceived healthiness and purchase intentions. Appetite. 2021;157. doi: 10.1016/j.appet.2020.104995. PubMed PMID: 33068665.
131. Devia G, Forli S, Vidal L, Curutchet MR, Ares G. References to home-made and natural foods on the labels of ultra-processed products increase healthfulness perception and purchase intention: Insights for policy making. Food Qual Prefer. 2021;88. doi: 10.1016/j.foodqual.2020.104110.
132. Egnell M, Boutron I, Péneau S, Ducrot P, Touvier M, Galan P, et al. Randomised controlled trial in an experimental online supermarket testing the effects of front-of-pack nutrition labelling on food purchasing intentions in a low-income population. BMJ open. 2021;11(2). doi: 10.1136/bmjopen-2020-041196. PubMed PMID: 33558350.
133. Egnell M, Galan P, Fialon M, Touvier M, Péneau S, Kesse-Guyot E, et al. The impact of the Nutri-Score front-of-pack nutrition label on purchasing intentions of unprocessed and processed foods: post-hoc analyses from three randomized controlled trials. Int J Behav Nutr Phys Act. 2021;18(1). doi: 10.1186/s12966-021-01108-9. PubMed PMID: 33731145.
134. Hall MG, Lazard AJ, Grummon AH, Higgins ICA, Bercholz M, Richter APC, et al. Designing warnings for sugary drinks: A randomized experiment with Latino parents and non-Latino parents. Prev Med. 2021;148:106562. Epub 2021/04/21. doi: 10.1016/j.ypmed.2021.106562. PubMed PMID: 33878350.
135. Mauri C, Grazzini L, Ulqinaku A, Poletti E. The effect of front-of-package nutrition labels on the choice of low sugar products. Psychol Mark. 2021. doi: 10.1002/mar.21473.
136. Mediano Stoltze F, Busey E, Taillie LPS, Dillman Carpentier FR. Impact of warning labels on reducing health halo effects of nutrient content claims on breakfast cereal packages: A mixed-measures experiment. Appetite. 2021;163. doi: 10.1016/j.appet.2021.105229. PubMed PMID: 33789168.
137. Packer J, Russell SJ, Ridout D, Hope S, Conolly A, Jessop C, et al. Assessing the effectiveness of front of pack labels: Findings from an online randomised-controlled experiment in a representative British sample. Nutrients. 2021;13(3):1-15. doi: 10.3390/nu13030900. PubMed PMID: 33802115.
138. Vanderlee L, Franco-Arellano B, Ahmed M, Oh A, Lou W, L'Abbé MR. The efficacy of 'high in' warning labels, health star and traffic light front-of-package labelling: An online randomised control trial. Public Health Nutr. 2021;24(1):62-74. doi: 10.1017/S1368980020003213. PubMed PMID: 33019950.
