# Supplementary material for: Impact of color-coded and warning nutrition labelling schemes: A systematic review and network meta-analysis
Source: PLoS Med. 2021 Oct 5;18(10):e1003765. doi: 10.1371/journal.pmed.1003765 (PMC8491916; doi:10.1371/journal.pmed.1003765)
Supplement: Attachment — Submitted filename: song.pdf [file pmed.1003765.s022.pdf]

This paper describes a systematic review with meta–analysis of studies of nutrition labelling.

I was asked for a statistical report and I interpret that to include all aspects of the design and conduct of the study.

Points of detail

Page 5 Perhaps state why other FOPLs were excluded? Are they already known to be ineffective?

Page 7 Reference 46 is to a very old version of the netmeta package. There have been a number of enhancements and bug fixes in the intervening five years so I would suggest updating it and re–running the analyses would be wise.

Page 9 Will journalists and policy makers understand what an out–of–home sector is?

Page 10 Does mixed race mean all the participants were of mixed race or does it mean different races were represented in the sample? I must say I find the idea of a representative sample of the population in the countries mentioned being exclusively white hard to credit but if that is what the primary authors claimed then I suppose we and the current authors have to believe them.

Figures S2 to S4 I am afraid I am baffled by the statement that "The colored polygons represent multiarm trials in the network". Does this mean that all comparisons represented by edges of a polygon were multi-arm, or just some, and if the latter which? Why are the polygons of different saturations?

Figures S5 to S7 Are these plots of the head to head direct comparisons or the indirect ones?

Figure S8 I appreciate that there are many funnel plots so we cannot expect detailed scrutiny of each but, not for the first time, I am struck by the conflict between the Egger test results and the plots. Here for instance sub-panel E appears to show little evidence of small study effects but the Egger test has a p–value of 0.0031. There are other examples in other funnel plots.

Table S3 The text tells us that there were two studies in Spanish but I can only see one here (reference 105).

Table S26 I find this a more helpful way of displaying the results than Table 2 in the main text as we can compare the effect of the network analysis with the naive direct approach. It would, I think, be helpful to be consistent as I assume the values here should replicate the ones in Table 2 but they seem to be reciprocals. At least 0.61, 0.51 and 0.73 are the reciprocals of the values here 1.64, 1.96 and 1.37. I did not check any of the others.

Table S26 This does not seem to be called out in the main text so reducing its impact. In fact several of the other supplementary sections are not called out.

Points of more substance

Selection of control

The studies included in the meta–analysis use two different control groups: no label and Nutrition Facts Table (NFt). The authors have merged these into one category assuming that there is no difference between them. This seems to me to be a major mistake as the effect sizes presented are now a mixture in some proportion of the difference against no label and the difference against NFt. See Barth et al. (2013, Table 3) for an example in a different subject area with three different control conditions which were analysed separately and which turned out not to be equivalent. If there are extant studies comparing no label directly with NFt then they need adding but otherwise an indirect comparison would be available.

Experiment versus real–world

Some of the language used seems to me to obscure the difference between the experiments and the real–world studies by referring to purchasing when that only occurs in real settings. This seems to me to be a limitation which should be mentioned more prominently including in the abstract. Obviously this is not the authors' fault, we can only review the studies there are, not the ones we would like to have read.

Relation between statistics and discussion

There seem to be sections of the discussion which lack empirical justification from the analyses presented here. For instance page 16 starting at 'The performance of color–coded labels' to the end of the paragraph refers to a number of comparisons which could presumably have been analysed from the

authors' database but I do not think they have been provided in the text. If the authors database is insufficient to answer them one way or the other then I think the text should be deleted.

Summary

Some points for clarification and some more important points about the analysis.

Michael Dewey

References

J Barth, T Munder, H Gerger, E Nüesch, S Trelle, H Znoj, P Jüni, and P Cuijpers. Comparative efficacy of seven psychotherapeutic interventions for patients with depression: a network meta–analysis. *PLOS Medicine*, 10(5), 2013.
