# Supplementary material for: Impact of color-coded and warning nutrition labelling schemes: A systematic review and network meta-analysis
Source: PLoS Med. 2021 Oct 5;18(10):e1003765. doi: 10.1371/journal.pmed.1003765 (PMC8491916; doi:10.1371/journal.pmed.1003765)
Supplement: Attachment — Submitted filename: Reviewers comments 2021.1.31_final.docx [file pmed.1003765.s023.docx]

Thank you very much for your comments. We felt that your comments were quite helpful to the improvement of our paper, and we have made modifications according to them.

*Requests from the editors:*

1. *Please restructure the abstract using the PLOS Medicine headings (Background, Methods and Findings, Conclusions - "Methods and Findings" is a single subsection).*

The abstract has now been restructured as per PLOS Medicine requirements.

2. *In the Abstract, following text, assume that the OR's given in the brackets reflects the range of OR's for the point estimates for those specific four forms of displaying nutrition information? This is a somewhat unusual way of representing statistics for this type of study, where generally you'd expect an OR for the point estimate followed by 95% CIs pertaining to that point estimate. Some readers might find this presentation a bit confusing, so if the authors can use a more conventional presentation that might be helpful.*

*"We found that the Traffic-light labelling system (TLS), Nutri-score (NS), Nutrient Warning (NW) and Health Warning (HW) all increased the probability of selecting healthier products (OR: 1.71-2.83), and warning labels were particularly effective in reducing consumers' probability of selecting less healthy products (odds ratio [OR]: 0.61-0.62)".*

The OR estimates and 95%CIs reported in the abstract have now been changed to the conventional presentation (OR and 95%CI) for each point estimation. This paragraph now reads:

“We found that the Traffic-light labelling system (TLS), Nutri-score (NS), Nutrient Warning (NW) and Health Warning (HW) all increased the probability of selecting healthier products (Odds Ratios [ORs] and 95% confidence intervals [CIs]: TLS, 1.50 [1.20, 1.87]; NS, 1.67 [1.06, 2.63]; NW, 3.76 [2.89, 4.89]; HW, 1.71 [1.36, 2.16]), and warning labels were particularly effective in reducing consumers’ probability of selecting less healthy products (OR and 95%CI: NW, 0.62 [0.51, 0.75]; HW, 0.61 [0.51, 0.73]).”

3. *In the abstract, for the following statement, is it possible to add information on what the outcome measure is for "healthfulness"? Also note that "healthy/unhealthy" is often seen as British English but "healthful" is more US English, would suggest the authors go for one or the other rather than a mix.*

*"TLS, NS and NW increased the overall healthfulness (TLS: +3%; NS: +5.4%; NW: +3.1%)"*

The information on what the outcome measurement is for “healthfulness” has now been added to the abstract. It reads:

“TLS, NS and NW increased the overall healthfulness (healthfulness ratings of products purchased using Models such as Modified AHEI-2010, FSAm-NPS/HCSP, and SSAg/1 score: TLS, +3%; NS, +5.4%; NW, +3.1%)”.

In addition, we have now changed all “healthy/unhealthy” across the manuscript into “healthful/unhealthful” as per your suggestions.

4. *In the last sentence of the Abstract Methods and Findings section, please add a note about any key limitation(s) of the study's methodology.*

Two key limitations of the methodology have been added to the last two sentences of the Methods and Findings session. It reads:

“However, heterogeneity and inconsistency were high in the comparison across different label types. Additional research is needed given the lack of real-world studies and long-term impact assessments.”

5. *In the author summary, some of the bullets could be rephrased a bit to flow better:*

*- Evidence on the impact of each type of color-coded labels and warning labels on modifying consumers purchasing behaviours were mixed. - perhaps "Prior to this study, evidence on the impact of...... was mixed"*

*- The feasibility and effectiveness of each label type applied in different contexts were unclear. - perhaps "Previously, the feasibility and likely effectiveness of using different label types in different contexts was unclear".*

**Suggest also rephrase the following:*

*- We provided the most comprehensive evidence to guide policy-makers in choosing the optimal front-of-package labelling policies, which will also inspire the generalization of mandatory front-of-package labelling schemes and largely mitigate the noncommunicable chronic disease burden. - perhaps tone this down a bit - "We provide more comprehensive evidence to guide..... This evidence synthesis may inform further generalization of mandatory front-of-package labelling schemes and help to mitigate the burden of noncommunicable chronic disease"*

**Suggest perhaps the following bullet could instead be replaced by something noting any limitations of the study which might affect interpretation/generalisation?*

*- In the future, we will expand our network meta-analysis work by summarizing on the impact of interpretative front-of-package labelling schemes on industrial reformulation and consumers' actual consumption behaviours.*

The bullets have now been rephrased as per your suggestions. The last bullet in under the subtitle of “What do these findings mean?” has now been replaced by the limitation of this study. It reads:

“Future studies should focus on the impact of FoPLs on dietary consumption in individuals, and industrial reformulation at population level, especially in real-world settings and over a longer timeframe. This will provide crucial, robust and comprehensive evidence to guide policy making.”

6. *Please reformat the citation style into PLOS Medicine's format (should be straight forward if using referencing software) - this should use callouts formatted as sequential numerals in square brackets (not superscript).*

We have now re-formatted the reference according to the PLOS Medicine’s citation style.

-----------------------------------------------------------

*Comments from the reviewers:*

*Reviewer #1: The manuscript deals with an actual and highly relevant topic for policy making worldwide. The authors have done a tremendous amount of work summarizing and analyzing all the available information on the topic. However, some of the analyses are not clearly explained or presented, which makes it difficult to judge the conclusions reached by the authors. I recommend a major revision after major methodological issues are addressed. Based on the data report on the paper I cannot say whether results are valid or not. Please find below some detailed comments. When submitting a revised version of the manuscript, please include line number to facilitate the review.*

The line numbers and page numbers have now been included.

*Introduction*

1. *The manuscript should include a logic model explaining how front-of-package nutrition labelling (FOPNL) is expected to influence health outcomes. References to the health communication theory should be provided on the Introduction.*

As per your suggestion, a logic model has been added to the introduction to explain the underlying mechanisms of how FOPL influences health outcomes (see below).

**Figure 1**. The logic model of the impact of color-coded labels and warning labels on consumers’ behaviours based on health communication theory. Consumers at first pay attention to nutrition labels and then process the nutrition information to understand the healthfulness and nutrient profile of the products. Correct interpretation of labels would further lead to a change in consumers’ perception towards of the products and influence the purchasing and consumption behaviours by promoting the purchase of healthier items and discouraging the purchase of less healthy products. Attention to and understanding of labels were considered necessary in this process, but the negative perception of food products (e.g., perception of severe risk, negative emotion) is key in motivating changes in food choice (red box).

A brief explanation has also been included in this part with reference to health communication theory. It reads:

“Based on health communication theory and previous similar studies[8, 23-26] (see Table S1, Figure 1), visual attention to and understanding of labels are necessary components in the mechanism of modifying consumers’ behaviours, but the negative perception of food products (e.g. perception of severe risk, lower grade of healthfulness, lower level or frequency of recommended consumption for unhealthful products) is key to changing food choice[26]. The mechanism may be confounded by study population demographics (e.g. age, sex, ethnicity/race, knowledge of nutrition labels, frequency of grocery shopping, familiarity with the brand, weight conscious and health status), product categories, purpose of tasks and characteristics of labels (e.g. use of aids, label position, the presence of summarized or detailed information)[8, 24-28].”.

2. *The authors state that "**Observational studies found that the mandatory implementation of warning labels in Chile resulted in lower sales of beverages high in sugars, salt, saturated fat or energy". However, the Chilean law does not only include FOPNL, which makes impossible to disentangle the effects of the different components (FOPNL, marketing regulations and prohibition of products with warnings at schools). In addition, Study 21 only reports qualitative data from focus groups with a specific group of participants, so the authors should tune down their statements related to the mechanisms underlying the efficacy of the warnings.*

We have now rephrased the description of the real-world observational study in Chile, as well as the statement derived from reference 21 (now reference 22), as follows:

“Observational studies found that Chile’s Law of Food Labeling and Advertising, which included the mandatory implementation of warning labels nationwide, resulted in lower sales of beverages high in sugars, salt, saturated fat or energy[21], and was likely to improve understanding and utilisation, especially in children [22]”.

Methods

1. *The authors should include detailed information on the criteria used for assessing risk of bias. This is particularly relevant for risk of bias for measurement of the outcome and confounding, given that most studies were categorized as high risk of bias. The authors refer to Table S22 but do not provide any specific explanation of how it was assessed or the reasons underlying the classification.*

We have now updated the supplementary Table S9-11 with more detailed information on the score of each question under each domain. We also added an explanation of how the risk of bias was assessed for each domain and how the summary score of the risk of bias was generated in the Supplementary Methods. It reads:

“The ROB 2 tool is made up of 5 domains of bias: randomisation process, deviation from intended interventions, missing outcome data, measurement of outcome, and selection of reported results. For each domain, several questions were included to assess different aspects of each domain of bias, and for each question a response was provided by choosing an answer from "Yes", "Probably Yes", "No information", "Probably no" or "No". Then a domain-specific overall assessment of bias was classified as "Low risk", "High risk" or "Some concerns" using recommended algorithms. Finally, a summary of biases across domains was grouped into (1) "Low risk" if the study is judged to be at low risk of bias for all domains for this result; (2) "Some concerns" if study is judged to raise some concerns in at least one domain, but not to be at high risk of bias for any domain; or (3) "High risk" if the study is judged to be at high risk of bias in at least one domain. The full questionnaires and algorithms summarising the domain-specific risk of bias are available at <https://www.riskofbias.info/welcome/rob-2-0-tool>.”

“The ROBINS-I tool is made up of 7 domains of bias: bias due to confounding, bias in selection of participants into the study, bias in classification of interventions, bias due to deviations from intended interventions, bias due to missing data, bias in measurement of outcomes, and bias in selection of the reported results. For each domain, several questions were included to assess different aspects of each domain of bias, and for each question a response was provided by choosing an answer from "Yes", "Probably Yes", "No information", "Probably no" or "No". Then a domain-specific overall assessment of bias was classified as "Low risk", "Moderate risk", "Serious risk of bias" or "No information". Finally a summary of biases across domains was grouped into (1) "Low risk" if the study is judged to be at low risk of bias for all domains for this result; (2) "Moderate" if study is judged to be at low or moderate risk for all domains; (3) "Serious risk" if the study is judged to be at serious risk of bias in at least one domain, but not at critical risk of bias in any domain; (4) "Serious" if study is judged to be at critical risk of bias in at least one domain; or (5) "No information" if there is a lack of information in one or more key domains, and there is no clear indication that the study is at serious or critical risk of bias. To integrated with the risk of bias assessment results of randomised controlled studies using ROB 2, we categorised "Serious risk" and "Critical risk" into "High risk", as well as "No information" and "Moderate risk" into "Some concerns", when summarising risk across domains as "High risk". The full questionnaires and criteria used for summarising the domain-specific risk and overall risk are available at https://www.riskofbias.info/welcome/home/current-version-of-robins-i/robins-i-tool-2016.”

“The NHLBI study quality assessment tools is made up of 13 different aspects of bias in observational cohort and cross-sectional studies, including: research question, study population, groups recruited from the same population and uniform eligibility criteria, sample size justification, exposure assessed prior to outcome measurement, sufficient timeframe to see an effect, different levels of the exposure of interest, exposure measures and assessment, repeated exposure assessment, outcome measures, blinding of outcome assessors, follow-up rate and statistical analyses. For each question, a response was provided by choosing an answer from "Yes", "No information", or "Others (cannot determine, not applicable or not reported)". Then a domain-specific overall assessment of bias was classified as "Low risk", "Moderate risk", "Serious risk of bias" or "No information". The full questionnaires and criteria for each response are available at https://www.nhlbi.nih.gov/health-topics/study-quality-assessment-tools.”

2. *Studies on FOPNL have been conducted using very different methodologies and study designs. Therefore, it is not clear how the authors calculated and analyzed the data to report ORs for all the variables. This is a major concern given that conclusions are reached based on these estimates. A detailed description on how these ORs were calculated should be provided in the manuscript.*

We have now added a detailed explanation of how the odds ratio and relative mean difference were assessed in the session of Supplementary Methods. It reads:

“For categorical outcomes, odds ratios (ORs) and 95% confidence intervals (95%CIs) were collected from original studies as the summary estimates for categorical outcomes. When ORs and 95%CIs (or coefficient of label type in logistic regression model [β] and its standard error [SE]) were not directly provided for FOPL of interest in our study, two researchers (J.S and M.B) calculated the unadjusted ORs and 95%CIs independently, and double-checked by a third researcher (M.T) if there was any inconsistency. The formula is set up based on a 2 x 2 table:

$Odds ratio=ad/bc$	(1)

$SE\left( odds ratio \right)=\sqrt{\frac{1}{a}+\frac{1}{b}+\frac{1}{c}+\frac{1}{d}}$	(2)

In formula (1) and (2), a and b represent frequencies in the intervention group (or follow-up) with and without an outcome observed (e.g., selection of a healthier options in a food category), while c and d stand for frequencies in the control group (or baseline) with and without an outcome observed.

For continuous outcomes, relative mean difference and SE were estimated using the following formula:

$Relative mean difference=(X2-X1)/X1$	(3)

$SE\left( relative mean difference \right)=\frac{X2}{X1}\sqrt{\left[ \frac{{SE(X1)}^{2}}{{SE(X1)}^{2}}+\frac{{SE(X2)}^{2}}{{SE(X2)}^{2}}-2\rho\frac{SE(X1)\times SE(X2)}{X1\times X2} \right]}$	(4)

In formula (3) and (4), X1 and X2 represent estimate of continuous outcome in control (or baseline) and intervention (or follow-up) condition respectively; ρ is the correlation coefficient between sample in intervention condition and control condition, where ρ= 0 when participants in both intervention and control conditions were separately collected from different population, ρ= 0.9 for studies with participants remained the same in both intervention and control conditions. For intervention trials with pre-post design in both intervention and control group, relative mean difference and SE were calculated with alternative formula as follow:

$Relative mean difference=\frac{\left( X2-X20 \right)-\left( X1-X10 \right)}{X1-X10}$	(5)

$SE\left( relative mean difference \right)=\sqrt{\frac{{X2}^{2}}{{X20}^{2}}\left[ \frac{{SE\left( X20 \right)}^{2}}{{SE\left( X20 \right)}^{2}}+\frac{{SE\left( X2 \right)}^{2}}{{SE\left( X2 \right)}^{2}}-2\rho\frac{SE\left( X20 \right)\times SE\left( X2 \right)}{X20\times X2} \right]+\frac{{X1}^{2}}{{X10}^{2}}\left[ \frac{{SE\left( X10 \right)}^{2}}{{SE\left( X10 \right)}^{2}}+\frac{{SE\left( X1 \right)}^{2}}{{SE\left( X1 \right)}^{2}}-2\rho\frac{SE\left( X10 \right)\times SE\left( X1 \right)}{X10\times X1} \right]}$	(6)

where X1 and X10 represent estimate of continuous outcome of baseline and follow-up condition in control group, while X2 and X20 represent intervention group. ρ= 0.9 in (6).”.

3. *A similar comment applies to the network analysis, more detailed information should be provided to enable reviewers to actually judge the validity of the conclusions.*

We have now added a detailed explanation of how the network meta-analysis was conducted in the session of Supplementary Methods. It reads:

“In this systematic review, a range of front-of-package labelling (FOPL) systems were included as the competing intervention groups and we aimed to find out whether one FOPL system is more or less effective than other FOPL systems in modifying consumers’ behaviours. Additionally, only very few intervention studies compared the effects of two FOPLs directly, which made it even more difficult to make multiple comparisons using conventional meta-analysis method. For these reasons, we applied the frequentist network meta-analysis (NMA) method to synthesize studies, which allowed for the simultaneous analysis of more than two interventions.

Technically, NMA generate network estimates by pooling indirect and direct comparisons between interventions. Direct comparisons refer to the comparisons between two competing interventions, which often didn’t exist or were quite limited. Indirect comparisons evaluate the effect of interventions against the same control group, and usually assessed in randomised controlled trials or quasi-experiment trials. With sufficient indirect comparisons, it is possible for NMA to make estimation the direct comparison based on the network graph. A simple example is given below:

The direct effect estimated by NMA (EAB) can be calculated using the effects of indirect comparisons (EA0, EB0): EAB = EA0 - EB0. The estimated direct effect was then combined with the observed direct effect provided by previous studies (if available), which makes full use of all information available and draws robust conclusion of what is the most (or least) beneficial intervention given all assumptions met.

The key assumptions of NMA are “transitivity” and “consistency”. The “transitivity”, in principle, ensures that we can calculate the direct effect of intervention A versus B (A vs B) via A vs C and B vs C using the above-mentioned equation. In other word, the Group C is transitive. However, the “transitivity” assumption is more of a conceptual assumption, and a statistical assessment of the “consistency” assumption is needed to provide evidence on whether direct and indirect comparisons are consistent across interventions. The inconsistency is often evaluated in two levels: global and local. The global test provides an overall inconsistency based on all comparisons across different interventions, while the local test examines the inconsistency for each comparison individually. In our study, a node-splitting method is used to evaluate the consistency in NMA models, which splits our network estimates into direct and indirect evidence using back-calculation method. The violence of “consistency” assumption may indicate that the distribution of covariates or effect modifiers is not balanced across original studies. Sensitivity analysis or subgroup analysis is usually conducted to explore the effect modifiers, and meta-regression is usually recommended for to adjust for the inconsistency. In our study, we performed a sensitivity analysis including only randomised controlled trials to examine the possible effect modification led by study design, as well as a series of subgroup analysis to explore the influence of a range of covariates, such as age group, sex, study settings, etc. Due to the limited number of studies for most of the intervention groups and outcome measures, we did not perform the NMA meta-regression which would require a large number of studies to ensure a sufficient statistical power.”

*Results*

1. *The authors have included a huge number of Tables and Figures, which makes it difficult to follow the manuscript. I recommend the authors to reduce the number of Tables and Figures to facilitate understanding.*

We have now cut down on the number of supplementary materials to 12 supplementary tables and 6 supplementary figures to make it as succinct as possible.

2. *In Table 1, I recommend providing a more detailed split of the publication year of the articles. The authors grouped 2018-2020 and 1998-2018 without a clear rationale. Regarding countries, I think that summary information by continent or region of the world will be more informative than country income. For example, Chile and Uruguay are high income countries but are very different from European countries or the USA. Regarding SES, I do not agree with how the authors grouped studies as they may be using different criteria than the ones reported in the articles. They should refrain from re-categorizing variables reported in the studies. Finally, considering that most articles do not report race, I would remove this criteria.*

As per your suggestions, we have now adopted a more detailed split of the publication year after 2010 when most relevant publications emerged (Table 2). The split of country income has now been replaced by region of the world (Europe, North America, Latin America, Oceania, Asia and Africa), and the SES split has now been replaced by education, individual/family income and occupation as what were reported in the original studies. Race has been removed from Table 2 following your advice.

3. *Results for the different outcomes should be presented following the order of a logic model, e.g. consumer perceptions and attitudes come before purchase intention.*

The order of outcomes has been changed as per the logic model:

1. Study characteristics
2. Consumers’ understanding and perceptions of color-coded and warning labels
3. Consumer’s perceptions and attitudes towards foods and drinks
4. Changes in consumers’ purchasing and consuming behaviours or intentions
4. *The authors should clarify how "less healthy" and "healthier" were defined. This is particularly relevant given that studies tend to use different criteria.*

The definitions of “healthful” and “unhealthful” in this systematic review have now been added to the “Methods” – “Measurement of outcomes”. It reads:

“The healthful and unhealthful products were grouped based on the levels of salt/sodium, sugar, saturated fat and calories indicated by the front-of-package labelling systems. Products with warning texts, symbols or colors (e.g., red in Multiple Traffic Light Label) indicating high levels of sugar, salt, saturated fat or calories content per 100g or per 100ml (per 100g/ml) relative to reference intake, or overall low healthfulness (e.g. red in Single Traffic Light, or orange and red [D & E] in Nutriscore), were defined as unhealthful.”.

5. *Results from network analysis are not clearly presented.*

We have now changed the original Table 3-5 to Figure 4-6 in the main text to help better present the network analysis. The newly added Figure 4-6 are forest plots that showed both the effects estimated from direct and indirect comparisons, as well as the combined network estimates. The number and proportion of direct evidence (observations in original studies) were also presented in the forest plots.

6. *The sections consumer's perception and attitudes and consumers' understanding are actually very important but results are not presented in detail. The authors should choose between excluding these sections or providing additional information. This is particularly relevant given that these outcomes could define decisions to implement different FOPNL schemes. In their discussion section they say that the review evaluated the impact of color-coded and warning labels on "the psychological mechanism underpinning purchasing modification, including consumers' perceptions and liking for foods and drinks, as well as the understanding and evaluation of label attributes". However, detailed information is not provided.*

To keep a balance between the word limitation of the manuscript, the information necessary for interpreting the results of consumers’ perception and attitudes towards food and drinks, and understanding of FOPLs, we added Table 3-5 in the main text. We also made a more explicit link to the supplementary tables with full details. We hope that these tables will provide information detailed enough for readers’ understanding.

Objective understanding of labels: Table 3, Table S5, and Figure S3

Subjective understanding of labels: Table S5, and Figure S3

Perceived effectiveness and credibility: Table S5 and Figure S3

Salience and visual attention: Table S5, and Figure S3

Consumer’s perceptions and attitudes towards foods and drinks: Table 4-5, Table S4, and Figure S2

*Discussion*

1. *The discussion section should be rewritten and improved to provide based on an in-depth discussion of the results reported in the manuscript. In the current version, the authors discuss results that are not presented in the manuscript, e.g. "Nutrition labels with numerical information and technical terms ... added to the difficulty in understanding nutrition information". In addition, it is not clear how the authors draw some of their conclusions based on their results, e.g. "TLS is considered to provide more sufficient information than NS, but the latter is recalled more frequently".*

We drew the conclusion "TLS is considered to provide more sufficient information than NS, but the latter is recalled more frequently" based on the results reported under the subtitle of ”salience and visual attention”. To avoid confusion, we have now added the position where the corresponding results were reported to the discussion part.

The sentence “Nutrition labels with numerical information and technical terms ... added to the difficulty in understanding nutrition information" was the evidence observed from previous studies. To avoid confusion, we have now rewritten the entire paragraph (2^nd^ paragraph in Discussion). The first 9 lines of this paragraph talked about the previous evidence on the effectiveness and improved understanding of FOPLs, while the rest of the paragraph discussed how our findings supported previous evidence. It reads:

“Despite of the heterogeneity in label types, label locations, labelling formats, position, study population, study design and experimental settings across studies [8, 14, 24-40], FOPLs were generally considered to have positive effects on guiding consumers in making more healthful food choices, especially in populations with low socio-economic status and limited knowledge of nutrition labels[8, 53]. This may due to their relative ease of comprehension, with their use of non-numerical aids (e.g., symbols and graphs) and visible design (e.g. verbal descriptor, red colors), compared to back-of-package labelling systems (e.g., NFt) which use numerical information and various of technical terms (e.g. serving size, recommended daily amounts)[25, 26, 32], FOPLs with would simply the comprehension of labels [25-27, 29, 30]. Our study suggests that TLS, NS and NW apparently all performed much better than the control group (NFt or no back of package label) in comparing/ranking, mathematical estimation, recall or classification of nutrition information, while also highlighting the function of eye-catching designs (such as color-coding used in TLS and NS, and stop signs used in most Chilean style NW) in facilitating consumers’ understanding and utility of nutrition information.”

2. *For the interpretation of results, the psychological mechanisms underlying the different schemes have not been adequately discussed. In this sense, recent studies showing how framing effects influence the effect of FOPNL on consumers' choice or the potential role of heurisitics on consumers' perception of products with warnings are not included in the review nor discussed.*

We agree that framing effects may play an important role in directing health-related behaviours, and previous evidence also indicated that gain-framed (positive-framed) messages were more likely than loss-framed messages (negative framed) to encourage prevention behaviours, especially for cancer prevention, smoking cessation and physical activities. However, in our study, we did not find any significant difference between these two framing types on their effectiveness in changing the probability of healthful products. No studies were included for other behavioural outcomes. We have now included this finding in Results (line 464-467). It reads:

“Positive HW was not observed to differ from its alternatives on the probability of choosing more healthful products. Considering that only one original study provided direct evidence on the difference between positive and negative framed HW, the framing effect on consumers’ purchasing behaviours requires further investigation.”

3. *Some of the comments are not entirely accurate, particularly for a review on FOPNL. For example, the authors state "In addition,* *ultra-processed foods and drinks, which are usually high in free sugar, total fat, saturated fat and sodium, are generally labeled with a NW for at least one nutrient, but some of which may be labeled as healthy in TLS or NS (e.g., sugar sweetened beverages may have green lights on sodium and saturated fat contents when labeled with TLS)". I don't think it is accurate to say that the TLS would label sugar sweetened beverages as healthy, as it is not the purpose of the TLS. The problem is not the label per se, but how consumers may understand the information provided by the TLS. In this sense, the authors report one study showing this effect (Reference 61).*

We have now rewritten this point in Discussion to make it more accurately portray the difference between warning labels and color-coded labels (line 541-547). It reads:

“The performance of color-coded labels and warning labels on multiple dimensions makes it necessary for policy makers to weigh pros and cons according to local context. For example, in countries with high levels of NCDs and a food system dominated by ultra-processed food and drinks, NW or HW could help lower supply-driven consumption as they are known to be easier to interpret by consumers, who can recognize that they are applied to unhealthful products high in sugar, salt and saturated fat [56]”.

4. *The discussion of how TLS or NW may be adequate for countries with different socioeconomic status is based on experimental data. The authors state that warnings "may be more suitable for countries with high levels of citizens with low socioeconomic status, who may indicate a lack of nutrition knowledge and/or health consciousness, and who may be unable to pay for expensive healthier foods". This statement does not take into account the effect of the food environment on consumers' eating patterns. For example, the share of ultra-processed products consumed is higher in the USA or UK compared to Latin American countries. Therefore, I do not think that NW would not be effective in the USA or the UK, even if consumers may have "higher nutrition knowledge or ability to purchase healthier alternatives". The authors should revised their statements based on a food systems' approach. I recommend the review by Swinburn et al. published last year on the Lancet discussing the syndemic of obesity, undernutrition and climate change.*

Thanks for your suggestion. We have now revised the statements that you pointed out and linked the type of FoPL to the food system. Please find the revised version in our last response.

-----------------------------------------------------------

*Reviewer #2: Feng He and colleagues presented here a systematic review and network meta-analysis about the impact of color-coded and warning nutrition labelling schemes. The topic is particularly relevant as it is a timely public health issue with high stakes in the current international debates on food nutrition labels.*

*The study aims to assess the impact of the color-coded and warning labels, the most studied and promising labels, on changes in both intended and actual purchasing and consumption behaviours. The study aims too to gain insight into the underlying psychological mechanism based on health communication theory to explore the heterogeneity across label types*

*It is an excellent and very useful paper. The manuscript is clear and well-written. The description of the methods and results are all good. The literature review of peer-reviewed articles on the topic (118 articles) seems to me really exhaustive. The statistical methods used are appropriate.*

*The study addressed interesting and very important research questions. This is a fascinating piece of work using a strong methodology.*

*I have few comments.*

1. *Authors claimed they followed the flow chart of the PRISMA recommendations . However it is not identical to the PRISMA flow diagram described on the website https://eur01.safelinks.protection.outlook.com/?url=http%3A%2F%2Fprisma-statement.org%2F&amp;data=04%7C01%7C%7C53324ce417cd487add8308d8b63a5891%7C569df091b01340e386eebd9cb9e25814%7C0%7C0%7C637459709129582010%7CUnknown%7CTWFpbGZsb3d8eyJWIjoiMC4wLjAwMDAiLCJQIjoiV2luMzIiLCJBTiI6Ik1haWwiLCJXVCI6Mn0%3D%7C1000&amp;sdata=3K%2BJ9%2FF095uOyezFKRknnY60TW99RLscdxV%2FBFollJ8%3D&amp;reserved=0. Some information are missing : how many unique articled authord have found (excluding duplicates), or how many articles were obtained from other sources (for example, by reviewing the bibliographic references of the identified articles).*

We have now completed the missing information in flow diagram as below

2. *In their flow chart, authors indicate that 14,785 items are excluded without giving the reason: duplicates? Do not meet the inclusion criteria? If so, what criteria?*

The 14785 items were excluded based on the PICOS criteria presented in Table 1.

| **Criteria** | **Description** |
| --- | --- |
| **Population** | General population |
| **Intervention** | **Inclusion:** |
|  | ·   All color-coded labels and warning front-of-package labels: TLS, NS, NW and HW; |
|  | ·   For each of TLS, NW and HW, different formats were also separately included into analysis: summary TLS versus nutrient-specific TLS, negative message framing versus positive message framing, textual warning versus textual+non-textual warning (e.g., picture of tooth decay, stop sign). |
|  | **Exclusion:** |
|  | ·   Other color-coded labels (e.g., Wheel of health, ENL and SENS) were not included because they had been scrapped or no longer actively developed due to political reasons or lack of public popularity. |
|  | ·   Studies which combined effects of nutrition labels of included types and excluded types (e.g., TLS+HSR). |
|  | ·   Studies investigated the combined effect of nutrition labelling and other intervention (e.g., TLS+food placement). |
|  | ·   Studies assessing only the effect of nutritional education using traffic-light label as educational tools were not included. |
|  | ·   Studies that applied color-coding schemes (Traffic-Light label score, Nutri-score) only to categorized the healthfulness of products were excluded. |
| **Comparator** | ·   Absence of nutritional information (no-label) |
|  | ·   Only NFt present |
| **Outcome** | All measured outcome were included based on the health communication model. |
| **Study design** | ·   Randomized controlled trials (RCTs), including randomized controlled crossover trials and clustered randomized trials |
|  | ·   Quasi-experiment: |
|  | o   Non-equivalent control group design |
|  | o   Interrupted time-series design |
|  | o   One-group pre-post design |
|  | ·    Choice-based conjoint studies |
|  | ·   Cross-sectional studies evaluating at least two intervention labels, or one intervention labels and control condition (no-label exposure or NFt): only eligible for the secondary outcomes. |

3. *Two independent reviewers reviewed the articles. What was the percentage of discrepancy? Please precise.*

We have now clarified percentage of discrepancy in our manuscript. It reads:

“Inclusion and exclusion, data extraction and risk of bias were first assessed by two independent reviewers (J.S, M.B). A total of 16 discrepancies (13.6%) were found and were referred to a third reviewer (M.T)”.

4. *The conclusions on the objective understanding of FOP Nutrition label are questionnable . Authors conclude on the effect of the TLS but not so much NS whereas many studies performed in France, Spain, Germany, UK, the Netherlands, Bulgaria, Switzeland, Italy, Singapore, USA, Canada,. have focused on this point and show well the superiority of the NS...it is visible in the additional figure S4 and the difference in outcome between Figure A and C is not understandable...*

We double checked the original studies included for the outcomes of “objective understanding”, and we found that NS studies all focused on the comparison or ranking of overall healthfulness of products, except for one examining the ability to mathematically manipulate (estimate) the healthfulness of products. 11 studies compared NS versus control, 9 for NS versus TLS, and 7 for NS versus NW. Both direct evidence and our network estimates indicated that NS improved understanding in the task of comparing/ranking healthfulness, but did not show superior ability to TLS or NW. We have now updated the results for “objective understanding” of FOPLs and provided more detailed information in the newly added Table 3.

For the difference between A and C in additional Figure S4, figure A showed the probability of selecting less healthy options across labels, while figure C showed the purchase intention for less healthy products. Purchase intention, as we defined in the Table S2, is the self-reported ratings of intention to purchase less healthful/more healthful products. It is estimated as a continuous outcome, and summarized as the relative mean difference in the network meta-analysis. The probability of selecting healthy/unhealthy products, however, is estimated as a dichotomous outcome measuring the actual purchasing behaviour. It is summarized as the ORs in the network meta-analysis.

5. *Sometimes discussion is not consistent with results. In some places, authors highlight the performances of TLS in the discussion while this is true also on the NS (and it is said in the results).*

We have double checked the results and discussion and made revisions to ensure that they are consistent.

In terms of the NS, the results are summarized as below:

- Objective understanding: NS increased the ability to compare/rank and mathematically estimate the overall healthfulness of products.
- Subjective understanding: NS was considered easier to understand than the control condition.
- Salience: NS was thought to stand out and easier to recall.
- Perception towards food: NS also reduce the amount of unhealthful food that people intended to consume, outperforming TLS.
- Purchasing behaviours: NS reduced energy, fat and saturated fat in purchased products. NS also improved the overall healthfulness in shopping baskets, and outperformed TLS

In the discussion, the results regarding NS were described as below:

“Our study suggests that TLS, NS and NW apparently all performed much better than the control group (NFt or no back of package label) in comparing/ranking, mathematical estimation, recall or classification of nutrition information, while also highlighting the function of eye-catching designs (such as color-coding used in TLS and NS, and stop signs used in most Chilean style NW) in facilitating consumers’ understanding and utility of nutrition information”.

“Generally, our findings suggested that different FOPL types may dominate different outcome measures. Specifically, NS performed better in nudging the purchase of more healthful products than NW.”

“According to our network meta-analysis, TLS was considered to provide more sufficient information than NS, but the latter is recalled more frequently (see results in the “Salience and visual attention” session).”.

6. *The proposed outcomes in term of nutrients include only unfavourable nutrients (I agree this is a major point) but they do not include favourable nutrients, while it is a major point of interest for NS (and other FOP nutrition labels in the world such as australian HSR) to have effects on positive components, especially +++ fiber. It would be worthwhile to point out that issue, since the effects of FOP nutrition labels must be able to take into account all nutrients which could have an effect on health.*

Thanks for pointing out this issue. We have now added this to the limitations in the discussion. It reads:

“Finally, we only considered energy and unfavourable nutrients (e.g., sugar, salt, fat and saturated fat) that are common in various FOPLs and are considered the major risk factors of NCDs burden[56], but favourable nutrients (e.g., fiber, protein) are also components of interest in many FOPLs (e.g., NS and Health Star Rating) for their beneficial health effect[58, 59]. So far, few studies have evaluated the effect of FOPLs on favourable nutrients[60], and there has been a disagreement in the inclusion of favourable nutrients in FOPLs as they might have a health halo effect to products that are high in salt, sugar or fat[61, 62]. For these reasons, we did not summarised the results on unfavourable nutrients in this systematic review, and further studies are needed to clarify the pros and cons of favourable nutrients on color-coded and warning labels.”.

7. *Authors should include in their discussion the paper on serving sizes from Egnell et al (Egnell M, Kesse-Guyot E, Galan P, Touvier M, Rayner M, Jewell J, Breda J, Hercberg S, et Julia C. Impact of Front-of-Pack Nutrition Labels on Portion Size Selection : An Experimental Study in a French Cohort. Nutrients 10, no 9 (2018) : 1268) , especially when they talk about the 'gap in knowledge' between buying and consumption.*

The results reported in the study by Egnell et al were included in the network meta-analysis for the outcome “perceived amount of consumption for less healthful products”. In the current version of Results, it reads:

“NW reduced the amount and frequency people intended to consume unhealthful food and drinks in the most effectively way, apparently outperforming by NS and TLS”.

In the discussion, we have now added this point to the limitations in the hope that your suggestion has been fully covered. It reads:

“Firstly, compared to the relatively large amount of evidence on the purchasing behaviour elicited by FOPLs, the data on food consumption was quite limited. Our findings showed that both warning labels and color-coded labels would reduce the amount or frequency of unhealthful products people intended to consume, and warning labels might outperform color-coded, which could be seen as an indirect evidence that FOPLs are able to change dietary consumption. However, the research gap between purchase and actual intake of different nutrients remains to be validated by future studies, which is crucial to inform decision making on labelling policies.”

8. *The study of Acton et al. on the NS should not be taken into considération for analysis of Nutri-Score since, as Acton et all indicated in their methodology, they used the graphic format of NS in their study, they did not used the correct algorithm for the NS (this is responsible of important modification for classification fo foods)*

We agree that, in the algorithms used by Acton’s study, they adjusted the nutrition grade scores to “match those of the health star rating for the purposes of this study (i.e., 0.5 to 1 stars = ‘E’ nutrition grade; 1.5 to 2 stars = ‘D’; 2.5 to 3 stars = ‘C’; 3.5 to 4

stars = ‘B’; 4.5 to 5 stars = ‘A’)”. However, this study was included for the assessment of calorie, salt, sugar and saturated fat content of purchased products, regardless of the healthfulness of products or food categories. In that sense, we think it important? to include their study into the meta-analysis.

9. *In tables 2 and 3, some limits of the interval are not accurate (either they are significant and should be bold, or they are not and should not be ( I think it is a rounding problem). For ex : NW vs TLS (-0.051 (-0.103,0) ; TLS vs control (-0.026 (-0.051,0))*

We have now made clearer notes on the significant estimates, marked using “**” in Table 3-5, and in bold in Figure 4-6 (Figures were newly added according to the comments of another reviewers) in the revised manuscript.

10. *Under Tables 2 to 4, the word intervention has been replaced by the word interaction in the footnote*

We have now revised all mentions of ‘intervention” to “interaction” in the footnotes.

11. *I did not understand what the N column correspond to in tables 2 to 4. I thought it was the number of studies included in the aggregation of the statistical parameter, but if it was the case, there shouldn't be zeros.. Please clarify.*

The N is the number of studies (corrected in the revised manuscript). The reason why there were zeros in some cases, has now been clarified in the footnotes. It reads:

“**Zeros in the number of comparisons mean that there were no direct evidence (original studies) included in the network meta-analysis.”.

-----------------------------------------------------------

*Reviewer #3:*

*Impact of color-coded and warning nutrition labelling schemes: a systematic review and network meta-analysis.*

*Song et al*

*This well conducted systematic review and meta-analysis aimed to analyse the impact of color-coded interpretive labels and warning labels on consumer purchasing behaviour.*

*The results are potentially important for policy makers and the wider public health community, as are the potential insights into the underlying psychological mechanisms of behavioral change.*

*ABSTRACT*

*Generally good.*

1. *Results, line 16 Here, and at various points beyond, please replace "showed" by "suggested". (Best to be a little modest and circumspect, given the limitations of the data, and analyses).*

We have now made the revision as per your suggestion.

*INTRODUCTION*

1. *Line 6 "To mitigate the healthcare burden resulting from NCDs, providing clear information about the nutritional profile of products is a recognised method to nudge consumers to healthier food and drink options 8." Please also mention that nutrition labelling also puts pressure on manufacturers to reformulate. Currently that important concept is not mentioned until late in the manuscript (Page 19). "..stimulate reformulation in the food industry53,54."*

A revision has been made as you suggested. It now reads:

“To mitigate the healthcare burden resulting from NCDs, providing clear information about the nutritional profile of products is a recognised method to nudge consumers to more healthful food and drink options and exert pressure on manufacturers to carry out reformulation to improve the nutritional profile of their products [8, 9]”.

2. *Para 1, penultimate line "systems, such as the Guideline Daily Amount, convey nutritional content, allowing consumers .", Please say "systems, such as the Guideline Daily Amount, convey nutritional content as numbers rather than graphics, allowing consumers .",*

A revision has been made as follows:

“Non-interpretive FOPL systems, such as the Guideline Daily Amount, convey nutritional content as numbers rather than graphics, symbols or colors, requiring consumers to create their own judgements on healthfulness.”.

*METHODS*

1. *The protocol of this systematic review was registered on PROSPERO (CRD42020161877). – Good*

Thanks for your comments

2. *Search Strategy. They searched four databases. Did they do any hand-searching, or consultation of topic-experts?? If not, they likely missed some studies.*

In addition to the database searching (15058 articles found), we also manually searched records by reviewing the bibliographic references of the identified articles (8 articles found in this way). This have now been noted in the “Search Strategy” as well as in the Figure 2 (Flow diagram). The search strategy and databases have been discussed and a consensus reached within the review panel. Most of the review panel members are experts in the field of nutrition policies, and many of them have abundant experience in meta-analysis. Therefore, we thought that our search strategy covered most of the related peer-reviewed studies by the time of submission.

3. *They sensibly identified one Primary Outcome: changes in consumers' purchasing and consumption behaviours, and relegated two other outcomes to Secondary status: a) consumer's perceptions and attitudes towards products, and b) consumers' understanding and perceptions of color-coded and warning labels.*

Thanks for your comments.

4. *P9. "Inclusion and exclusion, data extraction and risk of bias were first assessed by one reviewer (J.S), independently reviewed by a second reviewer (M.B)". Good.*

Thanks for your comments.

*RESULTS*

*Generally good.*

1. *Most studies were carried out in a laboratory setting (94%). Obvious issues of generalisability to real world settings. This is picked up in the Discussion. 74% had a high risk of bias. What % were industry funded?*

We have double checked the original studies and none of them were funded by industry. We have added this point in the 2nd paragraph of Results.

2. *Tables 2 & 3 are particularly informative, but complicated. Might it be possible to present the key findings as a graphic, perhaps a Forrest Plot?*

Thank you for this excellent suggestion. Tables 2 & 3 have now been replaced with Figure 4-6 (forest plots).

3. *The language throughout the manuscript needs to be toned down, and made a bit less categorical Eg Abstract "Results. A total of 135 studies nested in 120 articles were incorporated into the systematic review, of which 118 studies in 105 articles were eligible for meta-analysis. We found that the Traffic-light labelling system (TLS), Nutri-score (NS), Nutrient Warning (NW) and Health Warning (HW) all INCREASED the probability of selecting healthier products (OR: 1.71-2.83),." Given the limitations of the data, and analyses, it would be wiser to say: "We found that the Traffic-light labelling system (TLS), Nutri-score (NS), Nutrient Warning (NW) and Health Warning (HW) APPARENTLY all increased the probability of selecting healthier products (OR: 1.71-2.83),."*

*Likewise, for instance, in the main Results, Page 16, line 2: "When color-coded labels and warning labels were compared against each other, we found that NS WAS more effective than NW in promoting the probability of purchasing healthier products (OR and 95%CI: 1.64 [1.09, 2.48]),." Better to say: "When color-coded labels and warning labels were compared against each other, we found that NS APPEARED more effective than NW in promoting the probability of purchasing healthier products (OR and 95%CI: 1.64 [1.09, 2.48]),."*

*In general, almost every use of the term "showing" would be more honestly stated by using the term "suggesting". That would not detract at all from the authority or main messages of this comprehensive analysis.*

We have now toned down the language as per your suggestions above.

3. *The results sections also needs at least one sentence summarising the results of the Funnel Plot analyses.*

We have added a few sentences summarizing the results of the funnel plots. It reads:

“The comparison-adjusted funnel plots showed that there were no publication bias detected for most outcomes (Figure S7-9). For a few outcomes (e.g., probability of selecting more healthful options, energy and sugar of purchased products, objective understanding and perceived effectiveness of FOPLs) presenting funnel asymmetry, we cannot simply predict them as publication biases either as controversy remains in the test accuracy as the appearance of the plot may be affected by the coding of outcomes and choice of measures [55].”

*DISCUSSION*

*Generally good.*

1. *Page 18 states " our study found that FOPLs had a positive effect on guiding consumers in making healthier food choices, especially in populations with low socio-economic status and limited knowledge of nutrition labels 8,52." However, I cannot see any results text to support the statement about "especially in low SE status" ??*

In the original manuscript, we included the subgroup analysis results (e.g., grouped by SES) in the supplementary materials. However, following the advice by Reviewer #1, we have now removed the SES subgroup analysis. We also considered subgroup analysis by other socioeconomic factors, i.e. education, individual/familial income and occupations. However, most studies did not clarify the education, income or occupation of their study population (Table 2), thus the subgroup analyses by socioeconomic factors were not be carried out. For the above-mentioned reasons, we have now deleted the sentence you pointed out.

*Limitations para.*

*Generally good.*

1. *But it would be nice to see an acknowledgement that some some studies were probably missed, however, that would be unlikely to alter the main messages.*

We have now added your suggestion to our fourth limitation in the revised manuscript. It reads:

“Fourth, we only searched for peer-reviewed articles in four of the most commonly used databases and the bibliographic references of eligible articles to ensure that our search strategy could be easily replicated and repeated for update. We believed that most relevant studies should have been covered in this way, though some relevant studies reported in other databases or in the grey literature may have been missed.”

*Acknowledgements*

1. *It might be sensible to state how the various authors were funded (mainly to pre-empt subsequent unwarranted accusations of conflicts of interest).*

We double-checked the guideline of PLOS Med (<https://journals.plos.org/plosmedicine/s/submission-guidelines>) and found the following statement:

“Do not include funding sources in the Acknowledgments or anywhere else in the manuscript file. Funding information should only be entered in the financial disclosure section of the submission system”.

In that sense, we have provided the funding sources information in the submission system.

*Supplement*

*generally informative.*

1. *I would like to see the very useful Figure S5 promoted into the main manuscript; Perhaps split into two figures, one covering the more "objective" measures of purchasing/consumption (calories, salt, fat etc), the other the various psychological/behavioural intentions.*

We have now added the forest plots for the primary outcomes (probability of selecting more/less healthful products, purchase intention of more/less healthful products, overall healthfulness and total amount of single nutrient of purchased products (Figure 4-5),

For the key components in psychological mechanisms based on the health communication theory - objective understanding of labels, and perceived healthfulness and disease risk of more/less healthful products, we added tables in the main manuscript to help present the network estimates, as well as direct and indirect estimates (as suggested by Reviewer #1, Table 3-5).

To avoid figures too large, tables and figures for the rest outcomes remained in the supplementary materials, but we have inserted explicit link in the main text to help read. We hope that this revision will help improve the understanding of our findings reported in this manuscript.

2. *The PICOS Table (Table S1 PICOS criteria for inclusion and exclusion of studies) might also be usefully promoted into the main manuscript.*

We have now move the PICOS table to the main manuscript (Table 1).

*Nil else.*

-----------------------------------------------------------

*Reviewer #4: See attachment*

*Michael Dewey*

*This paper describes a systematic review with meta-analysis of studies of nutrition labelling.*

*I was asked for a statistical report and I interpret that to include all aspects of the design and conduct of the study.*

*Points of detail*

1. *Page 5 Perhaps state why other FOPLs were excluded? Are they already known to be ineffective?*

We have now added a PICOS criteria table (Table 1) and have explained why other FOPLs were excluded, it reads:

“Other color-coded labels (e.g., Wheel of health, ENL and SENS) were not included because they had been scrapped or no longer actively developed due to political reasons or lack of public popularity”.

For the reason why we did not include other FOPLs as reference group, we have added the explanation under the subtitle of “Inclusion and exclusion criteria, data extraction and bias assessment”. It reads:

“Studies which featured other interpretive or non-interpretive FOPLs (e.g., Guideline Daily Amount, health star rating) as the reference group were not eligible as they differed too much from the control group specified (i.e., back-of-package labels and no-label control).”

2. *Page 7 Reference 46 is to a very old version of the netmeta package. There have been a number of enhancements and bug fixes in the intervening five years so I would suggest updating it and re-running the analyses would be wise.*

Thanks for pointing out this issue. We have double checked that the netmeta package that we used is the v 1.2-1, which is updated in 2020 April, the latest version by the time we submitted our manuscript. We have added the version information of netmeta package to the main text (last sentence in Methods).

3. *Page 9 Will journalists and policy makers understand what an out-of-home sector is?*

To avoid any confusion, we have briefly defined OOH in the footnotes of Table 2. It reads:

“Out-of-home sector includes any outlet where food or drink is prepared for immediate consumption by consumers, such as restaurants, cafes, and takeaways”.

4. *Page 10 Does mixed race mean all the participants were of mixed race or does it mean di
   erent races were represented in the sample? I must say I find the idea of a representative sample of the population in the countries mentioned being exclusively white hard to credit but if that is what the primary authors claimed then I suppose we and the current authors have to believe them.*

In the original manuscript, “27 studies in white population” means that the participants were primarily white, not exclusively white. In the revised version, we have now removed the results for race/ethnicity because most studies (71%) did not stated the race of their study population, which was suggested by Reviewer #1.

5. *Figures S2 to S4 I am afraid I am baffled by the statement that "The colored polygons represent multiarm trials in the network". Does this mean that all comparisons represented by edges of a polygon were multi-arm, or just some, and if the latter which? Why are the polygons of different saturations?*

In the original network graphs, polygons of different saturation represented different multi-arm trials. Edges of a polygon represented different comparisons estimated in each trial. However, as suggested by another reviewer that too many supplementary figures and tables were provided which made it difficult to follow, we have now decided to remove the Figure S1-3 (network graphs) as it simply indicated the number of studies for each comparison, and this information was repeated in the subsequent supplementary figures and tables. We hope that this change will make it readable to you.

6. *Figures S5 to S7 Are these plots of the head to head direct comparisons or the indirect ones?*

These are network meta-analysis results that combined direct and indirect estimates (network estimates). We have now added the “network estimates” to the titles of these figures to make it more explicit to readers.

7. *Figure S8 I appreciate that there are many funnel plots so we cannot expect detailed scrutiny of each but, not for the first time, I am struck by the conflict between the Egger test results and the plots. Here for instance sub-panel E appears to show little evidence of small study effects but the Egger test has a p-value of 0.0031. There are other examples in other funnel plots.*

We have double checked the results and confirmed that the results of Egger test were correct. As suggested by Reviewer #1, we have now added a description of the funnel plot test, which took the funnel plots as a reference to predict publication bias, but not a standard. It reads:

“The comparison-adjusted funnel showed that the publication bias arising from newly developed labels with beneficial effects being more likely to be published may not present in our networks for most outcomes (Figure S7-9). For the funnel asymmetry for a few outcomes (e.g., probability of selecting more healthful options, energy and sugar of purchased products, objective understanding and perceived effectiveness of FOPLs), it was also difficult to predict the publication bias as controversy remains in the test accuracy as the appearance of the plot may be affected by the coding of outcomes and choice of measures[54]”.

8. *Table S3 The text tells us that there were two studies in Spanish but I can only see one here (reference 105).*

Another is reference 72 (Galan P, Egnell M, Salas-Salvadó J, et al. Understanding of different front-of-package labels by the Spanish population: Results of a comparative study. Endocrinol Diabetes Nutr 2019).

9. *Table S26 I find this a more helpful way of displaying the results than Table 2 in the main text as we can compare the effect of the network analysis with the naive direct approach. It would, I think, be helpful to be consistent as I assume the values here should replicate the ones in Table 2 but they seem to be reciprocals. At least 0.61, 0.51 and 0.73 are the reciprocals of the values here 1.64, 1.96 and 1.37. I did not check any of the others.*

Thank you for your suggestions and we have now replaced the Table 2 with Figure 4 in the format of forest plots. The results have also be modified to be consistent with the one s reported in the texts.

**Figure 4.** The network effects of different label types on the purchasing behaviour or self-reported purchase intention of products. RMD refers to the percentage of change comparing intervention with control group (RMD = (x2-x1)/x1; x1: mean of continuous outcome in the intervention group or after implementation of intervention, x2: mean of continuous outcome in the control group or before implementation of intervention). *The direct and indirect effects were observed significantly inconsistent (p < 0.05) and the network estimate may violate the assumption of consistency and transitivity of network meta-analysis, thus only direct evidence was used for interpretation. **Estimates with significant effect given α = 0.05 and β= 80%. Abbreviations: TLS, traffic light labelling system; NS, Nutri-score; NW, nutrient-based warning; HW, health warning; RMD, relative mean difference; OR, odds ratio; CI, confidence interval.

10. *Table S26 This does not seem to be called out in the main text so reducing its impact. In fact several of the other supplementary sections are not called out.*

As advised by you and Reviewer #1, we have cut down the number of supplementary figures and tables to make sure that they were as succinct as possible. The original Table 26 provided information basically the same as the newly added Table 3-5 and Figure 4-6, thus they have now been removed.

*Points of more substance*

*Selection of control*

1. *The studies included in the meta-analysis use two different control groups: no label and Nutrition Facts Table (NFt). The authors have merged these into one category assuming that there is no difference between them. This seems to me to be a major mistake as the effect sizes presented are now a mixture in some proportion of the difference against no label and the difference against NFt. See Barth et al. (2013, Table 3) for an example in a different subject area with three different control conditions which were analysed separately and which turned out not to be equivalent. If there are extant studies comparing no label directly with NFt then they need adding but otherwise an indirect comparison would be available.*

Thanks for your suggestion. Most of studies included used no-label as control condition (77.04%), and we have included the type of control condition (NFt or no-label control) in the subgroup analysis (Supplemetary Table S6). Results were similar between merged control (main analysis) and no-label control, suggesting that the additional presence of NFt would not lead to much changes to results. Here are several reasons for using merged control instead of no-label control in the main analysis, which have now been added to the “Method” session for explanation:

(1) Previous FOPL meta-analysis also adopted the same strategy that merging NFt with no-label control (Grummon et al, 2020. PMID: 32433660).
(2) Increase the statistical power and precision of network meta-analysis (Faltinsen et al, 2018. PMID: 29595131)
(3) In practice, back-of-package (e.g., NFt or its equivalent) is prevalent in many countries. Public interest focuses on whether the additional presence of FOPLs would further increase the effectiveness of nutrition labels in guiding dietary consumption, rather than substituting the ongoing NFt with FOPLs.

*Experiment versus real-world*

1. *Some of the language used seems to me to obscure the difference between the experiments and the real-world studies by referring to purchasing when that only occurs in real settings. This seems to me to be a limitation which should be mentioned more prominently including in the abstract. Obviously this is not the authors' fault, we can only review the studies there are, not the ones we would like to have read.*

Thanks for pointing out this issue. We have now rewritten this point in the Discussion (limitation). It reads:

“Secondly, most of the studies were laboratory experiments and thus findings mainly indicate the immediate or short-term effect of color-coded and warning label interventions. There were very few real-world studies assessing the effect of mandatory labelling policies on genuine “purchase”. However, considering the highly controlled condition in RCTs and quasi-experiments, our findings will need further demonstration by real-world evidence to build the evidence for generalization of labelling to other parts of the world. In our meta-analysis, we found that most of the existing real-world studies evaluated TLS and found it effectively increased the purchase/selection of more healthful products. For NS, NW and other HW types, more research in real-world settings is needed to confirm their effectiveness.”

In the abstract, we also added this limitation to the “Methods and Finding”. It reads:

“However, heterogeneity and inconsistency were high in the comparison across different label types. Additional research is needed given the lack of real-world studies and long-term impact assessments.”

*Relation between statistics and discussion*

1. *There seem to be sections of the discussion which lack empirical justification from the analyses presented here. For instance page 16 starting at `The performance of color-coded labels' to the end of the paragraph refers to a number of comparisons which could presumably have been analysed from the authors' database but I do not think they have been provided in the text. If the authors database is insufficient to answer them one way or the other then I think the text should be deleted.*

We have now rewritten this part to make it consistent between results and discussion. In addition, according to Reviewer #1, we also made revisions to make it more accurately portray the difference between warning labels and color-coded labels. It reads:

“The performance of color-coded labels and warning labels on multiple dimensions make it necessary for policy makers to weigh pros and cons according to local context. For example, in countries with high levels of NCDs which place a large burden on individuals and healthcare systems, and a food system dominated by ultra-processed food and drinks, NW or HW could help lower supply-driven consumption as they are known to be easier to interpret by consumers, in addition to encouraging reformulation by manufacturers to avoid having to display a warning on product packaging [56]”.

*Summary*

*Some points for clarication and some more important points about the analysis.*

*Michael Dewey*
