# Supplementary material for: Impact of color-coded and warning nutrition labelling schemes: A systematic review and network meta-analysis
Source: PLoS Med. 2021 Oct 5;18(10):e1003765. doi: 10.1371/journal.pmed.1003765 (PMC8491916; doi:10.1371/journal.pmed.1003765)
Supplement: Attachment — Submitted filename: Rebuttal letter 6.8.docx [file pmed.1003765.s024.docx]

Thank you very much for your comments. They are very helpful and we have made some further changes accordingly.

Requests from the editors:

1. Please update the search to the end of March, 2021, say.

Response: Thanks for your suggestions. We have now updated the search to the end of 24 May 2021, and 16 more studies nested in 15 articles were added to this meta-analysis. The results were similar to those in the previous version and have now been updated throughout the revised manuscript.

2. Please make that "quasi-experimental studies" in the abstract and elsewhere.

Response: Thank you. We have now replaced “quasi-experiments” with “quasi-experimental studies” throughout the manuscript as suggested.

3. Please quote the numbers of randomized and non-randomized studies included, around line 11 of the abstract.

Response: We have now quoted the number of randomised and non-randomised studies in the abstract (lines 11-12). It reads:

*“A total of 156 studies (including 101 RCTs and 55 non-RCTs) nested in 138 articles were incorporated into the systematic review”*

4. In the abstract and elsewhere in the paper, please indicate where you are quoting findings from randomized and non-randomized studies. Where data from the latter study designs are included, please adapt the language used so as not to imply causality, e.g., "... were associated with reduced probability of consumers purchasing ...".

Response: We have now toned down our findings as suggested. For example, in abstract (lines 13-15), it reads:

*“We found that the Traffic-light Labelling System (TLS), Nutrient Warning (NW) and Health Warning (HW) were all associated with an increased probability of selecting more healthful products”.*

5. Please adapt the final sentence of the "Methods and findings" subsection of your abstract so that it begins "Study limitations include ..." or similar, and quotes 2-3 of the study's main limitations.

Response: The main limitations have now been adapted as suggested. It reads (abstract lines 33-35):

*“Study limitations include high heterogeneity and inconsistency in the comparison across different label types, limited number of real-world studies and lack of long-term impact assessments”.*

6. Throughout the text, please adapt reference call-outs so that they are preceded by a space, and contain no spaces within the square brackets, e.g., "... nutrition labels [24,54].".

Response: The reference call-outs have been reformatted as suggested.

7. In the reference list, please use the journal name abbreviation "PLoS Med.".

Response: The reference list has been adapted as suggested.

8. Please break the PRISMA checklist out into a separate attached file, labelled "S1_PRISMA_Checklist" or similar and referred to as such in the text.

Response: The PRISMA checklist has been relabeled as "S1_PRISMA_Checklist", and referred to as such in the text (line 148). It reads:

*“The systematic review was conducted and reported in compliance with the Preferred Reporting Items for Systematic Reviews and Meta-analyses (PRISMA) Network Meta-analysis guideline (see S1 PRISMA Checklist)”*

9. In the checklist, please refer to individual items by section (e.g., "Methods") and paragraph number, not by line or page numbers, as these generally change in the event of publication.

Response: Thank you. The checklist has been refilled as suggested (see S1_PRISMA_Checklist).

Comments from the reviewers:

*** Reviewer #1:

The authors have largely improved their manuscript according to the reviewers' suggestions. However, there are several major issues that have not been adequately addressed and deserve further consideration.

1. As suggested, the authors have included a logic model to explain the effect of FOPL on consumer behavior. However, the model deserves revision. Attention is a pre-requisite for perception and understanding. Intention to purchase or consume is not the same as purchase or consumption. This should be clarified in the model, even if the authors grouped the two outcomes for analysis. Additional explanations should be included in the text. The authors refer to "negative perception of food products" as a requirement to modify food choice. This is not necessarily the case. FOPL can potentially modify choice by increasing healthiness perception (e.g. health logos). Something similar occurs with references to negative emotions (Line 126). I recommend the authors to more carefully examine the literature on the effects of FOPL on consumer perception and behavior. In Line 114, the authors refer to "confounding", which is not accurate. The characteristics of the schemes, product category and personal characteristics moderate the influence of FOPL on consumer behavior. This should be clearly explained in the manuscript.

Response: Thanks for your comments. On the basis of your suggestions, we have made further changes on the logic model and corresponding texts. The logic model (Fig 1) now looks like this:

Fig 1. The logic model of the impact of color-coded labels and warning labels on consumers’ behaviours based on health communication theory. Attention to labels is pre-requisite for understanding of labels and forming perception towards labels (amber box), but the mechanisms connecting changes in label understanding and perception with behavioural changes differ across FOPL types. Warning labels (e.g., NW and HW) elicit negative perception of food products (e.g., perception of severe risk, negative emotion) in motivating changes in food choice, while interpretive color-coded labels (e.g., TLS and NS) improve the perception of healthfulness for healthier food options (red box). Demographic characteristics, knowledge of labels, nutrition and health, food categories and experiment settings also modify the effects of FOPLs throughout the mechanism (blue box).

The corresponding texts to the logic model reads (line 108-131):

*“Based on health communication theory and previous similar studies[22-26] (see Fig 1), visual attention to labels are pre-requisite for the perception and understanding of FOPLs, but the mechanisms linking label interpretation and behavioural changes differ across types of FOPLs. Warning labels may elicit negative perception of unhealthful foods (e.g., perception of severe risk, lower grade of healthfulness, lower level or frequency of recommended consumption for unhealthful products) in the process of changing food choice. However, interpretative color-coded labels (e.g., TLS) tend to modify purchasing behaviour by increasing the perception of healthfulness for healthier food options. The effects of FOPLs are also modified by study population demographics, knowledge of nutrition labels, frequency of grocery shopping, familiarity with the brand, level of weight consciousness and health status, product categories, and characteristics of labels [23-28]. For example, women and people with special diet needs or higher perceived nutrition knowledge are more likely to look at FOPLs; the provision of serving size information or percentage of recommended intake may add to consumers’ difficulty in understanding FOPLs. Due to the above-mentioned reasons, experimental evidence regarding the effectiveness of FOPL in modifying consumers’ purchasing or consuming behaviours is mixed[14,23-40]. Meta-analyses showed that interpretative color-coded FOPLs (e.g., TLS) significantly increased consumers’ selection of more healthful options from a range of products, as well as decreased calorie and salt content of food purchased[31,41], but no synthesised results for warning labels were available. Experimental studies also provided some insights into the underlying mechanisms of how color-coded and warning labels change consumers’ behaviours, suggesting that warning labels effectively elicited negative emotions and raised health awareness, which lead to the ultimate modifications in food choice; on the other hand, color-coded labels (e.g., TLS) have a favourable effect on increasing consumers’ preference for healthier foods[25,27,28,31]”.*

2. Throughout the manuscript, the authors refer to "colour coded" FOPL and merge traffic-light with Nutriscore. However, these two schemes are conceptually different. What about the differences between the two schemes? The rationale for grouping these two schemes should be better explained and their differences further discussed.

Response: Thanks for your comment. In this study, we categorised Traffic Light Label (TLS) and Nutriscore (NS) into the category of “color-coded labels” when summarising results similar between TLS and NS, just like what we did for the category of warning label (including both nutrient-based warnings and health warnings). However, when doing the analysis, we separated these two types of FOPLs as they are slightly different in some aspects, and we did find some differences between these two labels. For example, “*Comparison between the two color-coded labels suggested that NS might perform better than TLS in comparing/ranking overall healthfulness task*.” (Results lines 333-332).

According to your suggestion, we have now added a few sentences in the discussion (lines 505-514) to help illustrate the reason why we included TLS and NS into the category of “color-coded label”, as well as their differences. We also explained the different findings for TLS and NS as per your comment #6. It reads:

*“…but the perception of labels might differ, especially between the two color-coded labels (TLS and NS). Although TLS and NS both used colour scales to indicate healthfulness of foods, TLS scores the level of each target nutrient while NS summarises the overall nutritional quality taking all preferable and detrimental nutrients into consideration. Therefore, according to our study, TLS was perceived to provide sufficient information, which was also reflected in the finding that TLS was associated with a better performance in complex understanding tasks (e.g., classification of sugar and saturated fat). However, too much information could also elicit confusion in consumers when reading TLS. NS, on the other hand, was perceived to be more salient and thus thought easier to understand for consumers”.*

3. Grouping of the outcomes should respond to the logic model. As I previously commented, salience and attention should not be grouped with understanding and perception. The term "recall" is used for expressing different outcomes. However, this is not clear in the manuscript. For example, Table 3 refers to recall of overall healthfulness (see Table 1) but the authors only refer to "recall"

Response: We have now changed our logic model as mentioned previously, and the outcomes of “perceived salience” and “visual attention” have been grouped into the outcome domain of “label perception”. (Fig 1)

For the problem in reference to “recall”, we have now made it clearer in tables (Table 3 and S5 Table). Thanks for pointing out this issue.

4. Some of the effects reported in the Tables were calculated based on 1 or 2 comparison. This should be acknowledged as a limitation of the analysis.

Response: We agree that the numbers of studies for some comparisons are limited, and have now added this to the limitations. It reads (line 620-625):

*“Finally, the numbers of studies are limited for some comparisons (e.g., only 1 study provided direct evidence on the comparison between NS and HW for the probability of purchasing more healthful products), especially in the analysis of outcomes concerning the perception and understanding of FOPLs, and perception and attitudes towards food options. Our findings for these secondary outcomes need to be validated by more studies in the future”.*

5. The discussion section lacks accuracy in many sections. The authors should be more careful in their description of the findings. I include below a couple of examples

Line 504: "perception of severe risk" there is no evidence to state that the labels raised associations with "perceived risk"

Response: Our results (shown in Table 5) found that NW and HW both were associated with an increased perceived risk of diseases, which support this statement (the previous line 504). To avoid any confusion, we now explicitly refer to the tables showing the corresponding results. It reads (lines 516-521):

*“NW and HW, compared with the color-coded counterparts, are more dependent on eliciting the perception of severe risk and negative emotions, which mediate the reduced selection of unhealthful products [26]. TLS and NS, on the other hand, rely more on enhancing the perception of healthfulness for more healthful options and thereby perform better at promoting purchase of healthful foods [27,28,31], which were supported by our findings (Table 4-5)”.*

Line 534: "remind consumers to eat less of unhealthful foods" what is the evidence for this?

Response: We have now added the corresponding results to lines 383-393 (results of “consumers’ perception and attitudes towards foods and drinks”). It reads,

*“The results indicate that TLS, NW and HW all reduced the perception of healthfulness for less healthful products (RMD and 95%CI: TLS, -0.077 [-0.116, -0.038]; NW, -0.224 [-0.263, -0.186]; HW, -0.126 [-0.16, -0.092]) and reduced the perceived recommended amount to consume unhealthful foods (Perceived recommended amount to consume: TLS, -0.05 [-0.054, -0.046]; NS, -0.08 [-0.082, -0.078]; NW, -0.47 [-0.509, -0.431]), but NW performed significantly better than TLS and HW (perceived healthfulness for unhealthful products: NW vs. TLS, -0.147 [-0.195, -0.1]; NW vs. NS, -0.188 [-0.301, -0.075]; Perceived recommended amount of unhealthful products to consume: NW vs. TLS, -0.42 [-0.459, -0.381]; NW vs. NS, -0.39 [-0.429, -0.351]; Perceived recommended frequency of unhealthful products to consume: NW vs. TLS, -0.41 [-0.449, -0.371]; NW vs. NS, -0.455 [-0.529, -0.381]) (Table 4, S4 Table and S1 Fig)”.*

To avoid any confusion in reading the discussion part, we now refer to the tables at lines 550-554, it reads:

“The underlying psychological mechanisms also vary across labels. Warning labels reduced the perceived healthfulness of unhealthful products and remind consumers to eat less of unhealthful foods, while color-coded labels enhanced the perception of healthfulness for more healthful products (Table 4 and S4 Table).”.

6. Lines 507-511: Evidence has shown that the traffic light system does not decrease healthfulness perception as much as warnings for products with low content of some nutrients. This should be further discussed.

Response: We have now made further explanation for this finding in the discussion paragraph (lines 516-529):

*“NW and HW, compared with the color-coded counterparts, are more dependent on eliciting the perception of severe risk and negative emotions, which mediate the reduced selection of unhealthful products [26]. TLS and NS, on the other hand, rely more on enhancing the perception of healthfulness for more healthful options and thereby perform better at promoting purchase of healthful foods [27,28,31], which were supported by our findings (Table 4-5). Our study, together with previous evidence, explained the role of perception of healthfulness in mediating the effect of color-coded labels and warning labels on consumers’ purchasing behavior. Warning labels are more often associated with “danger” as the use of symbols (e.g., octagon stopping sign), color (black and white), and cautionary texts, thus elicit negative perception and emotions towards unhealthful food products marked with warnings on the front of packages. For healthful products, warnings are not displayed, but TLS is presented with green lights on, which is indicative of the concept of “health”, “nature” and “sustainability”, thus associated with perception of better healthfulness for food products.”*

7. Differences between TFL and Nutriscore should be discussed in depth in the discussion, as they provide different types of information.

Response: Thank you for the suggestion. As mentioned in our response to your comment #2, we have added the comparison between TLS and Nutriscore in the discussion as follow (lines 505-514):

*“…but the perception of labels might differ, especially between the two color-coded labels (TLS and NS). Although TLS and NS both used colour scales to indicate healthfulness of foods, TLS scores the level of each target nutrient while NS summarises the overall nutritional quality taking all preferable and detrimental nutrients into consideration. Therefore, according to our study, TLS was perceived to provide sufficient information, which was also reflected in the finding that TLS was associated with a better performance in complex understanding tasks (e.g., classification of sugar and saturated fat). However, too much information could also elicit confusion in consumers when reading TLS. NS, on the other hand, was perceived to be more salient and thus thought easier to understand for consumers”.*

*** Reviewer #2:

1. Authors have satisfactorily taken into consideration my different comments I think this paper is relevant and particularly important and deserves to be published.

Response: Thanks for your comment.

*** Reviewer #4:

1. I still feel that combining no label and NFt is a mistake. The authors' rebuttal has not convinced me here. Their point 1 is that other people do it. That does not make it right. Their point 2 is about precision but I am concerned about bias. Their point 3 seems to suggest that they have done the wrong study. If the interest is in whether FOPLs increase the effectiveness of NFt then only those studies are relevant not the ones comparing FOPL with no label.

Response: Thanks for the comment. We agree that merging the no-label control with NFt control will increase the risk of bias in comparing intervention groups against control. Because most of the studies (80%) used no-label control, we have now added a sensitivity analysis excluding studies using NFt control setting (see S9 Table), and we also explained this in the “Method” (lines 260-262) and “Limitation” (lines 596-606) paragraphs. It reads:

Method” (line 260-262): *“Sensitivity analyses of the primary outcomes were conducted by (1) evaluating only RCTs, and (2) removing studies using NFt control.”*

Discussion (lines 602-611): *“Fifth, we did not conduct meta-regression to explore the heterogeneity and inconsistency between direct and indirect comparisons, due to the limited number of studies for most outcomes. Instead, we used a random-effect inconsistency model to accommodate the inconsistency and heterogeneity within and across comparisons. We also carried out a series of subgroup analyses by a range of effect modifiers based on previous studies, including age, sex, study setting, label location, accessibility of NFt, and product types. Another sensitivity analysis was also performed excluding studies using NFt as control setting. The results of the sensitivity analyses did not differ much from our findings in the primary analysis, which suggested the biases generated from combining two control settings (NFt control and no-label control) might be relatively small”.­*

2. I am not sure where the authors got the idea that I advocate removing material from the supplement or elsewhere. My view is that most studies are under-reported and I would usually be suggesting adding more not taking away. But that is a minor point.

Response: Sorry for the mistakes in our previous response. It was actually one of the other reviewers who recommended us to reduce the number of Tables and Figures to facilitate understanding, including those in supplementary materials, and only retained the most essential results to make the paper easier to read.
