# Supplementary material for: Impact of color-coded and warning nutrition labelling schemes: A systematic review and network meta-analysis
Source: PLoS Med. 2021 Oct 5;18(10):e1003765. doi: 10.1371/journal.pmed.1003765 (PMC8491916; doi:10.1371/journal.pmed.1003765)
Supplement: Attachment — Submitted filename: Rebuttal letter 2021.8.9.docx [file pmed.1003765.s025.docx]

Thank you very much for your comments. We have made some further changes as required.

Requests from the editors:

1. At line 9, please add a sentence, say, to state what the primary outcomes were.

Response: We have now stated the primary outcomes explicitly. It reads (lines 9-10)

*“…for the primary outcomes (measures of changes in consumers’ purchasing and consuming behaviours).”*

2. Around line 11, we ask you to note the number or proportion of laboratory studies included (alternatively, this could be quoted as a limitation).

Response: In the last sentence of the “Methods and Findings” paragraph, we added the proportion of laboratory studies to the study limitation. It reads (lines 31-33):

*“Study limitations include high heterogeneity and inconsistency in the comparison across different label types, limited number of real-world studies (95% were laboratory studies) and lack of long-term impact assessments”.*

3. At line 16, we think that "Nutri-score" (NS) needs to be spelt out at first use.

Response: NS now has been spelt out at the first use in the abstract. It reads (line 17):

*“Nutri-score (NS) and warning labels appeared effective in…”.*

4. At line 24, we suggest "... content of purchases.".

Response: The line 25 (previously line 24) has been changed as requested.

5. At line 28, we suggest "... nudging consumers towards the purchase ..." or similar.

Response: We have accepted your suggestion and made changes accordingly. It reads (line 28-29):

*“…color-coded labels perform better in nudging consumers towards the purchase of more healthful products”*

6. The sentence beginning at line 30 ("The difference could lie ...") is really a sentence of discussion, and we therefore ask you to remove it, and if you wish add a few words to this effect to the "Discussion" subsection of your abstract.

Response: We have now removed this sentence from the abstract.

7. At line 34, we ask you to substitute "impact" in place of "effectiveness", bearing in mind that not all the evidence included is from randomized studies.

Response: We have now replaced “effectiveness” with “impact” here. It reads (line 34):

*“Our systematic review provides comprehensive evidence for the impact of color-coded labels...”*

8. At line 96, please make that "...had implemented".

Response: Change has been made as required. The sentence now reads (line 96):

*“At the time of completing this manuscript, a total of 31 countries had implemented interpretive FOPL systems”.*

9. At line 158, should that be "2021" as in the abstract?

Response: Sorry for the typo. We have corrected it as “2021”. The sentence reads:

*“The search was updated on 24th May 2021 to capture the latest publications up to the submission of manuscript”.*

10. At line 492, please make that "Despite the heterogeneity ...".

Response: The sentence has been corrected as required. It reads (line 491):

*“Despite the heterogeneity in label types, label locations, labelling formats…”.*

11. Throughout the text, please add a space immediate preceding reference call-outs (e.g., "... their products [7,8].".

Response: The reference call-outs has now been reformatted as requested.

12. Please remove the quotation marks from the title for reference 21.

Response: The quotation marks have been removed.

13. Please spell out the "L" in the author of reference 45.

Response: The ref 45 has been now corrected as

*“National Heart, Lung, Blood Institute. Quality assessment tool for observational cohort and cross-sectional studies. Bethesda: National Institutes of Health, Department of Health and Human Services. 2014.”*

Comments from Reviewers:

Reviewer #1:

[supportive report received]

Reviewer #4:

The authors have addressed my second round of comments.

Michael Dewey

***

Thanks for your comments.
